# The Binding Mode of Second-Generation Sulfonamide Inhibitors of MurD: Clues for Rational Design of Potent MurD Inhibitors

**DOI:** 10.1371/journal.pone.0052817

**Published:** 2012-12-20

**Authors:** Mihael Simčič, Izidor Sosič, Milan Hodošček, Hélène Barreteau, Didier Blanot, Stanislav Gobec, Simona Golič Grdadolnik

**Affiliations:** 1 EN-FIST Centre of Excellence, Ljubljana, Slovenia; 2 Laboratory of Biomolecular Structure, National Institute of Chemistry, Ljubljana, Slovenia; 3 Faculty of Pharmacy, University of Ljubljana, Ljubljana, Slovenia; 4 Laboratory of Molecular Modeling, National Institute of Chemistry, Ljubljana, Slovenia; 5 Laboratoire des Enveloppes Bactériennes et Antibiotiques, Université Paris-Sud, Orsay, France; 6 Centre National de la Recherche Scientifique, Orsay, France; Spanish National Cancer Center, Spain

## Abstract

A series of optimized sulfonamide derivatives was recently reported as novel inhibitors of UDP-*N*-acetylmuramoyl-L-alanine:D-glutamate ligase (MurD). These are based on naphthalene-*N*-sulfonyl-D-glutamic acid and have the D-glutamic acid replaced with rigidified mimetics. Here we have defined the binding site of these novel ligands to MurD using ^1^H/^13^C heteronuclear single quantum correlation. The MurD protein was selectively ^13^C-labeled on the methyl groups of Ile (δ1 only), Leu and Val, and was isolated and purified. Crucial Ile, Leu and Val methyl groups in the vicinity of the ligand binding site were identified by comparison of chemical shift perturbation patterns among the ligands with various structural elements and known binding modes. The conformational and dynamic properties of the bound ligands and their binding interactions were examined using the transferred nuclear Overhauser effect and saturation transfer difference. In addition, the binding mode of these novel inhibitors was thoroughly examined using unrestrained molecular dynamics simulations. Our results reveal the complex dynamic behavior of ligand–MurD complexes and its influence on ligand–enzyme contacts. We further present important findings for the rational design of potent Mur ligase inhibitors.

## Introduction

The increasing rate of bacterial resistance against available antibacterial agents is becoming a serious threat to our society. Therefore, the development of new antimicrobial agents that act through new targets is an important task [1]. Peptidoglycan is one of the main components of the bacterial cell wall, and it represents one of the most frequently used targets for antibacterial agents. However, the intracellular steps of peptidoglycan synthesis have been greatly under-exploited. Only two such antibacterial agents are in clinical use: fosfomycin and D-cycloserine [2].

The Mur ligases are essential intracellular bacterial enzymes that are involved in the biosynthesis of peptidoglycan precursors and thus represent attractive targets for the development of novel antibiotics. The Mur ligase family comprises enzymes UDP-*N*-acetylmuramate:L-alanine ligase (MurC), UDP-*N*-acetylmuramoyl-L-alanine:D-glutamate ligase (MurD), UDP-*N*-acetylmuramoyl-L-alanyl-D-glutamate:*meso*-diaminopimelate ligase (MurE), and UDP-*N*-acetylmuramoyl-L-alanyl-γ-D-glutamyl-*meso*-diaminopimelate:D-alanyl-D-alanine ligase (MurF). These enzymes consecutively add L-Ala (MurC), D-Glu (MurD), *meso*-2,6-diaminopimelic acid (or L-Lys in most Gram-positive bacteria) (MurE), and D-Ala- D-Ala (MurF) to the nucleotide precursor uridine 5′-diphosphate-*N*-acetylmuramic acid (UDP-MurNAc) [3]. They also share a common reaction mechanism. In the first stage, the substrate is phosphorylated by ATP. The resulting acyl-phosphate intermediate is then attacked by the amino group of the incoming amino acid (or dipeptide). A high-energy, tetrahedral intermediate is produced that finally yields the nucleotide products, ADP and inorganic phosphate [3].

MurD from *Escherichia coli* is one of the most extensively studied enzymes of the Mur ligase family. Crystal structures of the apoenzyme and of complexes of the enzyme with bound inhibitors, natural substrates, and nucleotide product have been deposited in the Protein Data Bank (PDB) [4]-[11]. MurD ligase is composed of three globular domains: the *N*-terminal domain (residues 1 to 93) is involved in the binding of the UDP moiety of the UDP-*N*-acetylmuramoyl-L-alanine (UMA) substrate; the central domain (residues 94 to 298) binds ATP; and the *C*-terminal domain (residues 299 to 437) binds D-Glu [4]. The UMA substrate binds to MurD in a cleft formed between the *N*-terminal and the central domains. Crystal structures of MurD ligase have revealed two different conformations: ‘closed’ and ‘open’, which differ in the entirely distinct positions of the *C*-terminal domain. Two open structures of the MurD enzyme in the absence and presence of the UMA substrate are deposited in the PDB [6]. It is believed that ATP binding induces enzyme closure to the active conformation, followed by the binding of UMA and then of D-Glu, which binds last [6].

Several attempts have been made to design potent inhibitors of MurD. The first effective inhibitors were phosphinate derivatives [12], which act as analogs of the tetrahedral intermediate. There were a few other phosphinate inhibitors designed [13], [14], although none of these have antibacterial activity [15].

**Figure 1:**
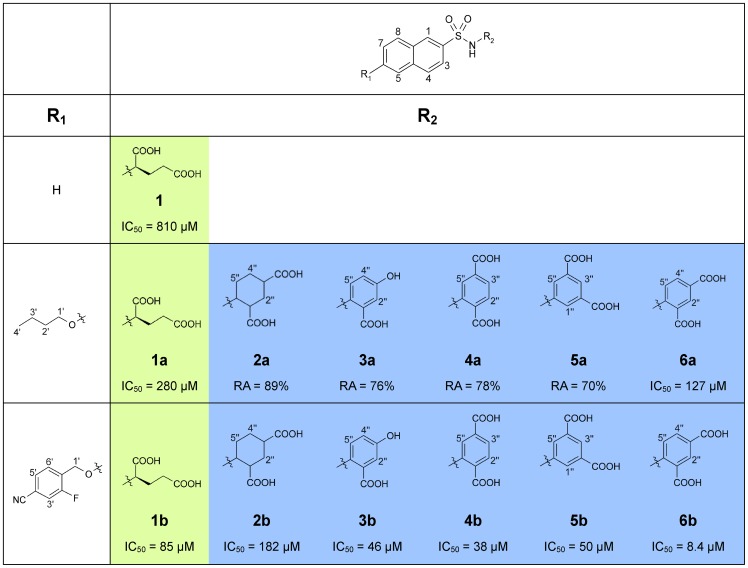
Structures of the investigated compounds from the first-generation and second-generation of MurD sulfonamide inhibitors. The MurD inhibitory activities and residual activities (RA) of these compounds were published previously [8], [11]. The first and the second generation sulfonamide inhibitors are marked in green and blue respectively.

Alternative functional groups that mimic the tetrahedral intermediate have been tested for their MurD inhibitory activity. A series of substituted naphthalene-*N*-sulfonyl-D-glutamic acid MurD inhibitors was synthesized [7], [8], where the most potent inhibitor was a C6-arylalkyloxy-substituted derivative, *N*-(6-(4-cyano-2-fluorobenzyloxy)naphthalene-2-sulfonamido)-D-glutamic acid **1b** (Figure 1) with an IC_50_ of 85 μM. 6-Butoxynaphthalene-2-sulfonamide derivatives containing D-glutamic acid (**1a**) and L-glutamic acid were the first two inhibitors for which the crystal structures in complex with the MurD protein were published [7]. Although MurD is highly stereospecific for D-glutamic acid [16], only small variations can be observed in the binding modes of D- and L-glutamic-acid-containing inhibitors, as determined by X-ray diffraction.

We recently performed extensive nuclear magnetic resonance (NMR) and molecular dynamics (MD) studies of the MurD binding modes of several naphthalene-*N*-sulfonyl-D-glutamic acid derivatives [17]. These data provided insight into the dynamic events in these ligand–enzyme complexes that cannot be observed in the crystal structures. Transferred nuclear Overhauser effect (NOE) investigations and MD trajectories revealed varying degrees of conformational flexibility of these bound ligands, which can be related to the variations in their activities. For example, mutually exclusive NOEs indicated naphthalene ring rotations that are much more pronounced in the less-active L-Glu derivative. Conformational flexibility can affect the adaptability of the ligand-binding site, and this is probably one of the important reasons for the only moderate activities of these naphthalene-*N*-sulfonyl-D-glutamic acid derivatives.

More recently, a second generation of sulfonamide inhibitors were synthesized that contain rigid mimetics of D-glutamic acid (Figure 1, **2a** **–** **6a, 2b** **–** **6b**); these were also evaluated for MurD inhibition [11]. The main idea here was to improve the binding properties of the naphthalene-*N*-sulfonyl-D-glutamic acid derivatives by substitution of the flexible D-glutamic acid with rigid analogs based on benzene or cyclohexyl dicarboxylic acids. These compounds showed significantly improved inhibitory activities compared to the first generation of sulfonamide inhibitors. The most promising compound has an IC_50_ of 8.4 µM (Figure 1, **6b**). As was presented in our previous study [11] and is also in this study (Figure 1), only two R_1_ substituents were considered. The main reason for this is the fact that the co-crystal structures of inhibitors **1a** and **1b** with those R_1_ substituents were available [7], [8]; therefore, these structures enabled the structure-based design of new inhibitors. The X-ray data also enabled us to understand the higher potency of inhibitor **1b** with the *p*-cyano-2-fluorobenzyloxy group at position C6. The cyano group of this substituent forms additional hydrogen bonds, and its phenyl ring forms the π–π interactions and cation-π interaction with the MurD active site.

Comparisons of the dynamic properties of ligand–MurD complexes of these first and second generations of sulfonamide inhibitors, which have fragments with varying intrinsic flexibilities, will offer excellent opportunities for the upgrading of our knowledge regarding the dynamic events in these complexes. This will also be important for further rational design of more potent derivatives. Therefore, we performed extended solution-NMR and unrestrained-MD studies of these second generation sulfonamide inhibitors in complex with MurD.

Here we report the MurD binding modes arising from these NMR and MD studies of several of these novel inhibitors, including the six most active ones (Figure 1). These ligand–enzyme contacts were experimentally explored through maps of ^1^H/^13^C chemical-shift perturbations (CSPs) upon binding of novel and known ligands to MurD that was selectively labeled with ^13^C at the methyl groups of Ile (δ1), Val, and Leu and through ligand epitope maps obtained using saturation transfer difference (STD). The conformational and dynamic properties of the bound ligands were studied using transferred NOE correlation spectroscopy (NOESY). The influence of the conformational flexibility on the stability of the ligand–enzyme contacts was explored using unrestrained MD simulations. The effects of various D-Glu mimetics on the conformational and dynamic properties of these ligand–MurD complexes are presented here and related to the variations in the ligand inhibitory activities.

## Results and Discussion

### The ligand binding sites

**Figure 2:**
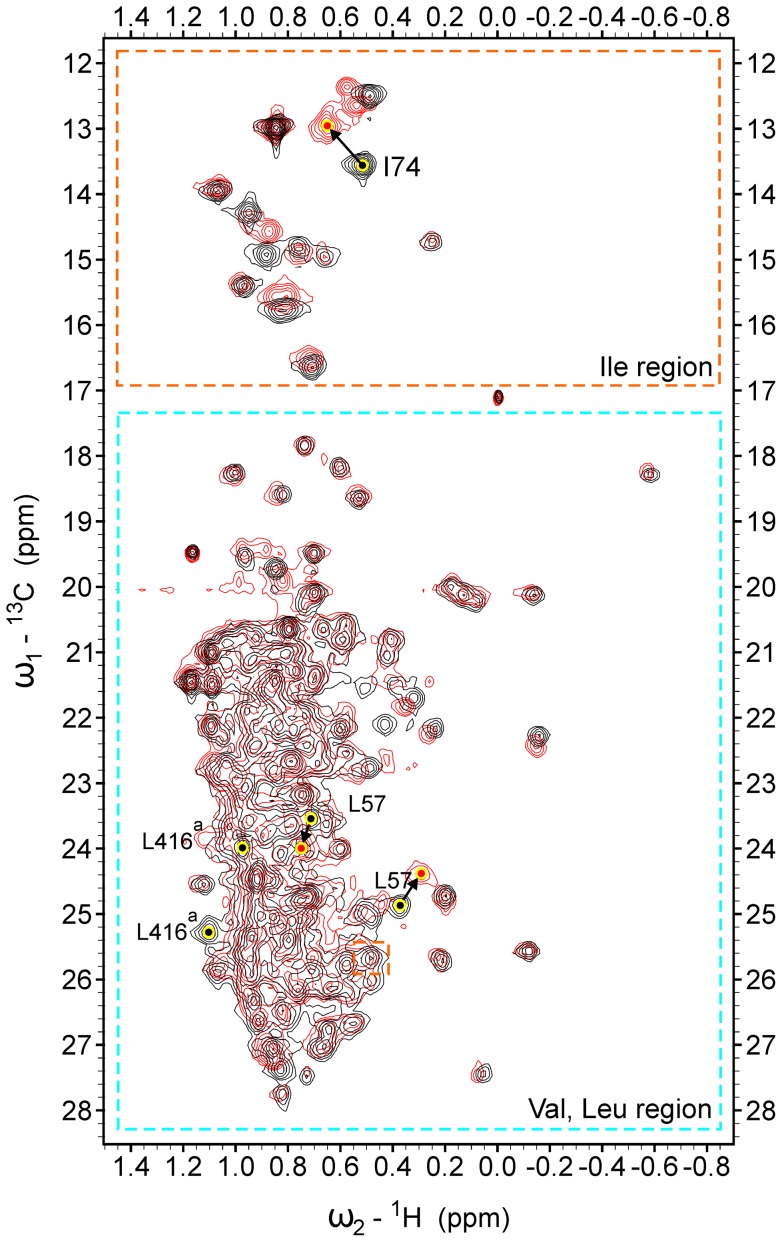
Overlay of ^1^H/^13^C HSQC NMR spectra in absence (black) and presence (red) of compound 6b. The ligand/protein ratio is 10∶1. The proposed assignment of crucial methyl groups is presented. ^a^ The signals of Leu416 methyl groups disappear at a ligand/protein ratio of 0.5∶1 The new position of these signals cannot be identified because of the signal overlap. In such cases, the minimum possible CSPs are calculated. The Ile and Val/Leu regions are marked with orange and light blue boxes respectively. ^b^ One Ile signal was deliberately folded into the Leu region.

**Figure 3:**
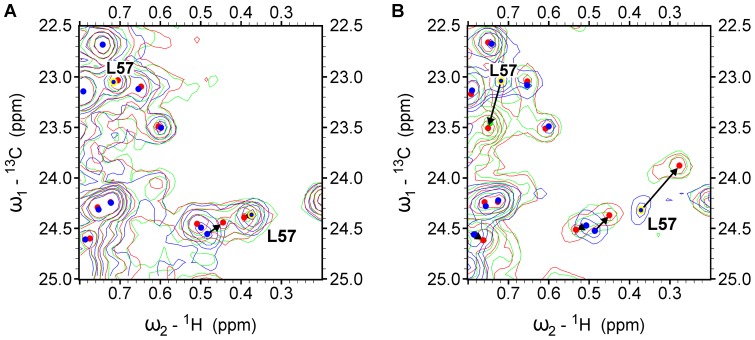
Selected expansions of ^1^H/^13^C HSQC spectra. Overlays of the three ^1^H/^13^C HSQC spectra of the MurD protein with increasing concentrations of selected ligands are used. The ligand/protein molar ratios are 0∶1 (blue), 1∶1 (green), 2∶1 (red). The positions of signal peaks at ligand/protein ratio 0∶1 and 2∶1 are marked with blue and red circles respectively. (**A**) Expansion of ^1^H/^13^C HSQC spectrum, indicating small or negligible CSP of Leu57 methyl groups, induced by the binding of compound **6a**. (**B**) Expansion of ^1^H/^13^C HSQC spectrum, indicating large CSP (intermediate exchange regime) of Leu57 methyl groups, induced by the binding of compound **6b**.

Sensitivity-enhanced ^1^H/^13^C heteronuclear single quantum correlation (HSQC) was used to determine the locations of novel ligands at the MurD binding site. MurD was selectively ^13^C-labeled at the Ile (δ1), Val, and Leu methyl groups. The numbers of these methyl groups in individual MurD domains are listed in Table 1. In the HSQC spectrum, the signals of all of the Ile (δ1) and 62% of the Val and Leu methyl groups are well resolved (Figure 2, Figure S1, Dataset S1, and Table S1). MurD was titrated with eleven naphthalene-*N*-sulfonyl derivatives (**1**, **1a**, **2a**, **5a**, **6a**, and **1b**–**6b**). In addition, a separate titration with β,γ-methyleneadenosine 5′-triphosphate (AMPPCP) was performed. By monitoring the changes in HSQC spectra during titration, we identified two types of exchange regime for the ligand–MurD complexes regarding the on/off rate of the ligand in comparison with the chemical shift differences of uncomplexed and complexed MurD signals [18]. For some resonances, we observed continuous chemical shift changes (fast exchange regime), while the resonance with the most pronounced CSPs broadens, disappears and reappears at various locations (intermediate exchange regime). The new positions of the signals in the case of the intermediate regime were not proposed just on the basis of one spectrum, but typically the spectra at each titration step of each ligand were carefully examined. Comparisons between influences of ligands with various structural elements on a particular signal were performed to identify the new position of shifted signals. An example is presented in Figure 3.

A complete assignment of the methyl resonances was not preformed because of the very low yields for the expression of the deuterated protein required for NMR assignment of the 47.7 kDa MurD. In addition, MurD is not stable at room temperature over several days. Only the crucial methyl resonances in the active site of MurD were identified using comparisons of the MurD CSP patterns induced by binding these novel and known ligands, published binding modes of various types of MurD ligands (as determined by X-ray [5], [7]–[11] and NMR studies [17], [19]), and their theoretically predicted proton chemical shifts in the program SHIFTS (Version 4.4.1) [20]. Regarding the MurD ligands investigated in this study using the HSQC method, the co-crystal structures of compounds **1a**, **1b**, and **2b** are available (PDB entries 2JFF [7], 2VTD [8], and 2XPC [11] respectively).

**Table 1:** Expected Ile (δ1), Val, and Leu methyl signals in the MurD protein.

Methyl group	overall	*N*-terminal domain	Central domain	*C*-terminal domain
Ile (δ1)	14	6	7	1
Val	68	16	34	18
Leu	112	22	50	40

**Figure 4:**
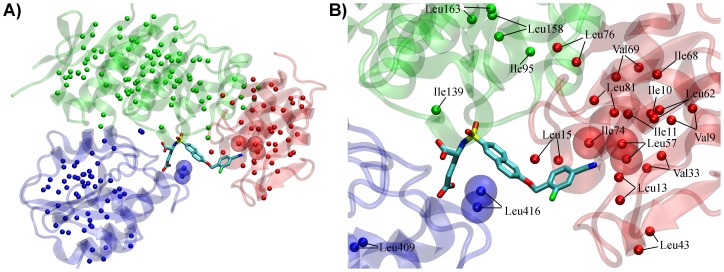
Ile (δ1), Val, and Leu methyl groups in MurD protein. (**A**) MurD protein in complex with compound **1b** (PDB entry 2VTD) [8]. Ile (δ1), Val, and Leu methyl groups are represented as spheres. The *N*-terminal domain, central domain, and *C*-terminal domains are colored in red, green, and blue respectively. (**B**) Close-up view of MurD binding site (PDB entry 2VTD) [8] with bound compound **1b**. Only the methyl groups within 12 Å of the ligand are shown. Methyl groups in the range of 5 Å are marked as transparent Van der Waals spheres.

From the known crystal structures of naphthalene-*N*-sulfonyl derivatives in complex with MurD [7], [8], [11], it is evident that the methyl groups of three selectively labeled residues are significantly closer to the ligands compared to the other labeled methyl groups. These are Leu416 in the *C*-terminal domain and Leu57 and Ile74 in the *N*-terminal domain (Figure 4). They are in the range of 5 Å to the ligand. Note that the methyl groups of Leu13, Leu15, and Ile139, which seem close to the ligand in the 2D presentation (Figure 4), are in the range of 10 Å, 11 Å and 12 Å to the ligand respectively. To clarify this point, the stereo plots (Figure S2) and chart of distances from all labeled methyl groups to the bound ligands **1a**, **1b**, and **2b** as measured from the co-crystal structures (Figure S4) are provided in the Supporting Information.

The Leu416 methyl groups are in the range of 4 Å relative to the naphthalene and D-Glu moiety of naphthalene-*N*-sulfonyl-D-Glu derivatives (Figure 4). In addition, Leu416 is the neighboring residue of Ser415 that forms hydrogen bonds with the D-Glu moiety [7], [8]. Any other ^13^C labeled methyl group of the *C*-terminal domain is more than 10 Å away. Moreover, the ^13^C labeled methyl groups in the central domain and *N*-terminal domain are also more than 9 Å away.

The methyl groups of Ile74 and Leu57 are in the range of 4 Å and 5 Å relative to the C6-substitents of the naphthalene*-N-*sulfonyl derivatives. Ile11 methyl group is further away (6.5 Å), while any other Ile methyl group of the *N*-terminal domain is more than 11 Å away. The next closest Val and Leu methyl groups, Leu81 and Val33, are in the range of 7 Å and 8 Å respectively. Any other Val/Leu methyl group is more than 9 Å away. In the *C*-terminal domain, Leu416 is in the range of 7 Å to the aromatic moiety of C6 substituent, while any other ^13^C labeled methyl group in the central domain or *C*-terminal domain is more than 16 Å away.

**Figure 5:**
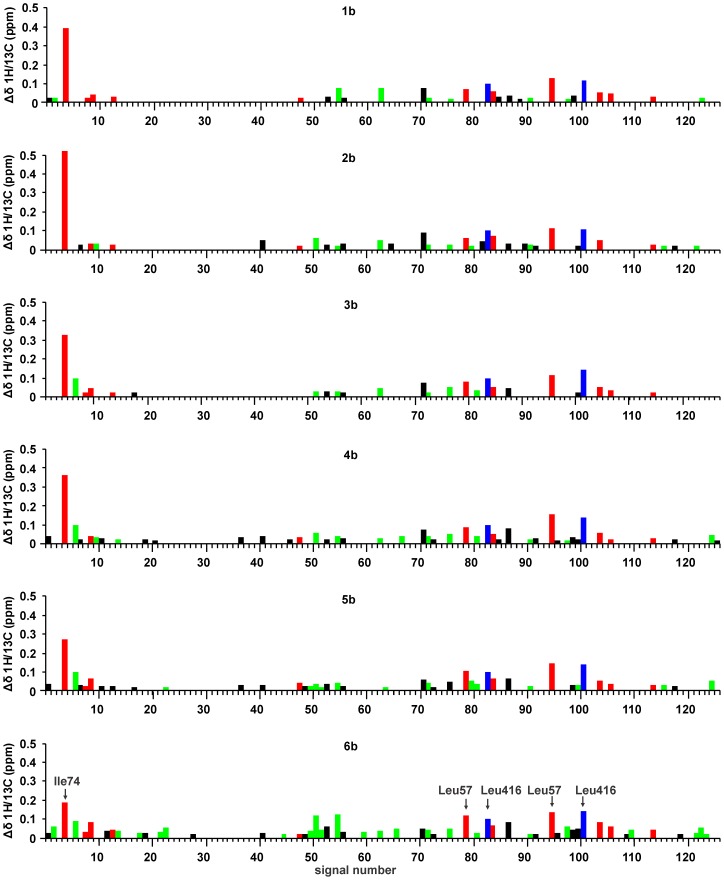
The CSP patterns of the ^13^C labeled methyl groups upon binding of C6-arylalkyloxy-naphthalene-*N*-sulfonamide derivatives. The CSPs in red, blue, and green correspond to the methyl groups near to the uracil binding site, the D-Glu binding site, and the cleft-forming region of the central domain respectively. Note that the numbering of CSPs does not correspond to the MurD residue numbers. The CSPs are numbered according to the positions of the signals in the ^13^C dimension of the ^1^H/^13^C HSQC spectrum, starting from the most up-field position. Only the values above the threshold of 0.02 ppm are shown to neglect the effects of the 2% variation in the dimethyl sulfoxide (DMSO)-*d*_6_ concentration at ligand titration.

**Figure 6:**
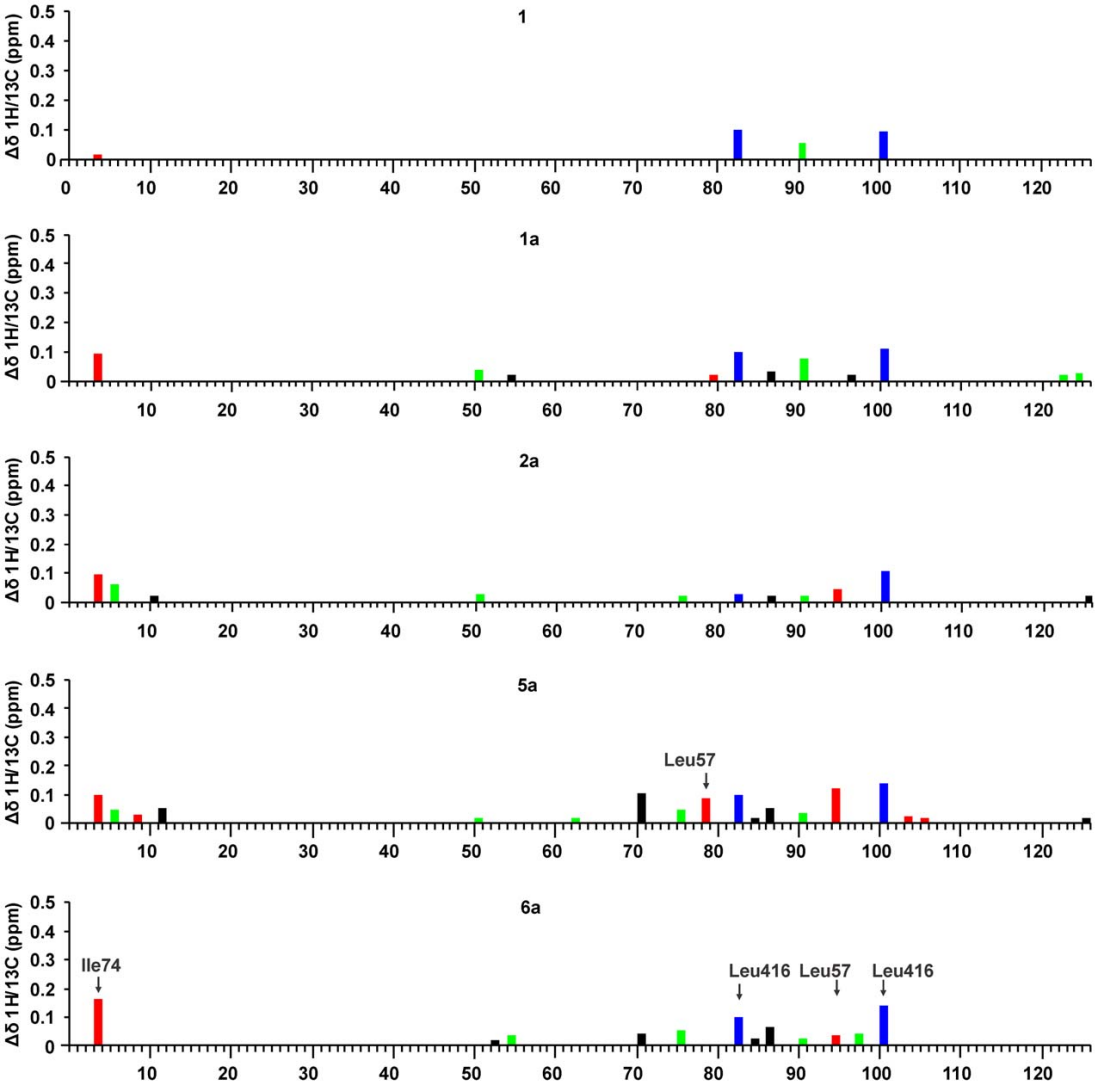
The CSP patterns of the ^13^C labeled methyl groups upon binding of C6-alkyloxy-naphthalene-*N*-sulfonamide derivatives. The CSPs in red, blue and green correspond to the methyl groups near to the uracil binding site, the D-Glu binding site, and the cleft-forming region of the central domain respectively. Note that the numbering of CSPs does not correspond to the MurD residue numbers. The CSPs are numbered according to the positions of the signals in the ^13^C dimension of the ^1^H/^13^C HSQC spectrum, starting from the most up-field position. Only the values above the threshold of 0.02 ppm are shown to neglect the effects of the 2% variation in the DMSO-*d*_6_ concentration at ligand titration.

The fact that five methyl groups of Leu57, Ile74, and Leu416 differ significantly from the rest of the labeled methyl groups regarding the spatial proximity to the specific structural elements of the bound ligands is used for the identification of corresponding signals in the HSQC spectra. The signals of these groups are expected to be significantly affected at binding of naphthalene-*N*-sulfonyl derivatives because of the ring current effects of naphthalene ring moiety or C6 arylalkyloxy substituents. The comparison of the CSPs' patterns upon binding of eleven ligands (Figures 5 and 6) reveals that only five signals have significantly larger CSPs at binding of one or the other ligand with a particular structural element.

Only one of these signals is located in the Ile region of the HSQC spectrum and can be assigned to Ile74 (Figure 2), which is confirmed by its significantly larger CSP at binding of C6 arylalkyloxy derivatives (**1b–6b**) (Figure 5) than at binding of the C6 alkyloxy derivatives (**1a–6a**) (Figure 6). The other four signals are located in the Leu region of the HSQC spectrum (Figure 2). Only two of these four signals are affected at binding of unsubstituted derivative **1** (Figure 6) and are assigned to Leu416. Their large CSP at binding of derivative **1** and all other naphthalene-*N*-sulfonyl derivatives (Figures 5 and 6) can be attributed to ring current effects of naphthalene moiety, which is common to all eleven ligands. In addition, the signals assigned to Leu416 are the only signals that are affected at binding of the D-Glu amino acid [11] but to a significantly lower extent. The remaining two signals can be assigned to Leu57, because they are affected only at binding of the C6 substituted derivatives and generally possess significantly larger CSPs at binding of C6 arylalkyloxy derivatives (**1b–6b**) than at binding of C6 alkyloxy derivatives (**1a–6a**) (Figures 3, 5, and 6). In addition, the pronounced variations in experimental ^1^H chemical shifts between signals assigned to the Leu416 and Leu57 methyl groups (Figure S1) are in agreement with the theoretically predicted values using crystal structures of the MurD complexes with the compounds **1a**, **1b**, and **2b** (Dataset S2).

In the above assignment strategy of the five closest labeled methyl groups, the remote conformational effects are neglected. Such an approach can be justified by the comparison of MurD crystal structures from complexes with various naphthalene-*N*-sulfonyl derivatives. The pronounced MurD conformational changes are not observed. The root mean square deviation (RMSD) for all heavy atoms between the MurD structures in complex with the compounds **1a**, **1b**, and **2b** are below 0.8 Å. The theoretically predicted ^1^H chemical shifts using the MurD crystal structures from these three complexes are also very similar (Dataset S2).

Other signals with lower CSPs cannot be assigned to a particular labeled residue. However, they are also informative for ligand-binding studies, because many of them can be grouped according to the positions of the residues with regard to the binding sites, such as: (i) the uracil-binding region in the *N*-terminal domain, which has significantly larger CSPs with the binding of the C6 arylalkyloxy derivatives than the C6 alkyloxy derivatives (Figures 5 and 6, CSPs in red); (ii) the D-Glu-binding region in the *C*-terminal domain (Figures 5 and 6, CSPs in blue) that is composed only of the signals assigned to Leu416, as the other selectively labeled methyl groups in the *C*-terminal domain are far from the binding sites; and (iii) the cleft-forming region in the central domain that is affected upon binding of sulfonamide derivatives and AMPPCP (Figures 5 and 6, CSPs in green). For the identification of the cleft-forming region, the fact that ATP binds to the central domain [5] as well as the determined CSP pattern during binding of AMPPCP (Figure S3, Dataset S1) are considered.

A general observation is that the CSPs of these investigated ligands are similar to the CSPs of their D-Glu derivatives **1a** and **1b** (Figures 5 and 6) for which the X-ray structures in complex with MurD are known [7], [8]. This indicates that these novel ligands bind to the same binding site, with the C6 substituent located in the uracil-binding pocket, the naphthalene ring positioned in the cleft between all three domains, and the rigid mimetic of D-glutamic acid located in the D-Glu-binding site.

The alkyloxy-substituted compounds have a much smaller effect on the CSPs in the uracil-binding pocket compared to the pronounced effects of arylalkyloxy-substituted compounds (Figures 3, 5, and 6). The fact that the overall effect of arylalkyloxy-substituted compounds on the CSPs is also larger indicates the importance of firm interactions in the uracil binding site for the stable binding interactions of all ligand segments. The most potent compound, **6b**, affects the largest number of signals and especially those belonging to the central domain residues (Figure 5), indicating the existence of additional interactions of **6b** with the central domain residues.

### Ligand conformation

The conformational properties of the bound ligands were studied using the application of transferred NOE experiments. The sign change of the ligand NOE cross peaks was observed upon addition of the enzyme. In NOESY spectra recorded in the absence of the enzyme, only a few very weak positive NOE cross-peaks were observed. Thus the contribution of the NOE contacts of the free ligand to the transferred NOE cross-peaks is negligible.

**Figure 7:**
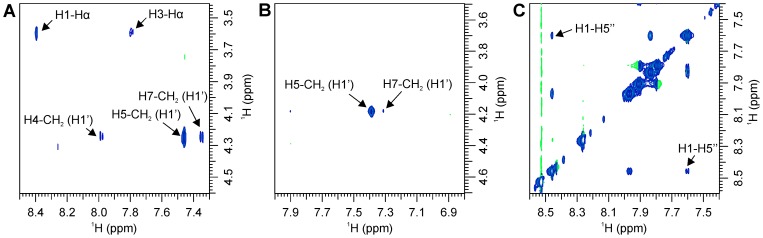
Selected expansions from transferred NOESY spectra. (**A**) The mutually exclusive H5–CH_2_(1′) and H7–CH_2_(1′) and the mutually exclusive H1–H^α^ and H3–H^α^ NOEs of compound **1a**. (**B**) The mutually exclusive H5–CH_2_(1′) and H7–CH_2_(1′) of compound **6a**. (**C**) The H1–H5′′ NOE of compound **6a**. Its H3–H5′′ NOE is detectable only at a lower contour level.

The nontrivial NOEs observed in the transferred NOESY spectra (Figure 7) and the calculated distances are listed in Table 2 and Table 3. Similar patterns of mutually exclusive NOEs, which indicate the conformational dynamics of the ligands at the receptor binding site, are seen as for the naphthalene-*N*-sulfonyl-D-Glu derivatives [17]. The simultaneous appearance of H1–H5′′ and H3–H5′′ NOEs might be a consequence of naphthalene ring rotations or rotations of the rigid D-Glu surrogates. The mutually exclusive H5–CH_2_(1′) and H7–CH_2_(1′) indicate the naphthalene ring rotations or various orientations of the C6 substituent.

In the C6 alkyloxy series (**1a–6a**), the replacement of the D-Glu moiety affects the variations in intensity between the H1–H5′′ and H3–H5′′ NOEs. These variations are significantly larger for derivatives with a substituted phenyl ring than the corresponding difference in intensity between the H1–H^α^ and H3–H^α^ NOEs for the D-Glu derivative **1a** because of the reduced intensity of the H3–H5′′ NOE (Figure 7). The variations in the distance values might be a consequence of the various population distributions between the exchanging conformers. Therefore, the reduced intensities of the H3–H5′′ NOEs can be attributed to the reduced populations of the corresponding conformers, which might be related to the reduced flexibilities of the constrained glutamic acid analogs of **1a** at the receptor binding site. This effect is not observed in the arylalkyloxy series.

**Table 2:** Nontrivial NOE connectivities and corresponding distances (Å) calculated from the transferred NOESY spectra for alkyloxy derivatives.

	1a	2a	3a	4a	5a	6a
**H1–H^α^**	3.2 (4.7)^a^					
**H3–H^α^**	3.1 (2.5)^a^					
**H5–H1**′	2.4 (2.4)^a^	2.5	2.3	2.4	2.5	2.6
**H7–H1**′	3.8 (4.3)^a^	weak^b^	2.9	weak^b^	weak^b^	4.3
**H1–H6**′′		3.4				
**H3–H6**′′		3.5				
**H1–H5**′′		4.3	3.2	3.1	^c^	3.0
**H3–H5**′′		weak^b^	4.0	4.2	^c^	weak^b^

For the sake of clarity, the atom labels do not strictly follow IUPAC rules for all compounds.

a Distances from X-ray structure, PDB code 2JFF [7]. ^b^ Observed in 1D trace from 2D spectrum.^ c^ Medium NOE cross-peak that can belong either to H1” or H5” due to signal overlap.

**Table 3:** Nontrivial NOE connectivities and corresponding distances (Å) calculated from the transferred NOESY spectra for arylalkyloxy derivatives.

	1b	2b	3b	4b	5b	6b
**H1–H^α^**	3.1 (3.5)^a^					
**H3–H^α^**	3.5 (3.9)^a^					
**H6**′**–H1**′	3.5 (3.5)^a^	3.5 (3.5)^b^	3.3	3.4	3.0	3.5
**H5– H1**′	2.3 (4.4)^a^	2.4 (2.0)^b^	2.4	2.4	2.4	2.6
**H7– H1**′	weak^d^ (2.9)^a^	4.0 (4.5)^b^	3.4	3.3	3.4	3.3
**H1–H6**′′		3.3 (3.2)^b^				
**H3–H6**′′		3.6 (4.6)^b^				
**H1–H5**′′		4.3 (3.7)^b^	3.1	2.8	d	3.1
**H3–H5**′′		4.0 (4.7)^b^	3.2	3.2	d	c

For the sake of clarity, the atom labels do not strictly follow IUPAC rules for all compounds.

a Distances from X-ray structure, PDB code 2VTD [8].

b Distances from X-ray structure, PDB code 2XPC [11].

c Overlapped.

d Medium NOE cross-peak that can belong either to H1” or H5” due to signal overlap.

The variations in the intensities of mutually exclusive NOEs between the various dicarboxyl substitution patterns are too insignificant to come to any conclusions about the influence of the phenyl ring substituent position on the flexibilities of the bound derivatives. We can speculate that the *ortho, para* positions with regard to the sulfonamide moiety reduce the flexibility because of the weakest H3–H5′′ NOE of compound **6a** that can be observed only in the 1D trace. Due to signal overlap, we cannot estimate this NOE for compound **6b**.

### Ligand epitope mapping

**Figure 8:**
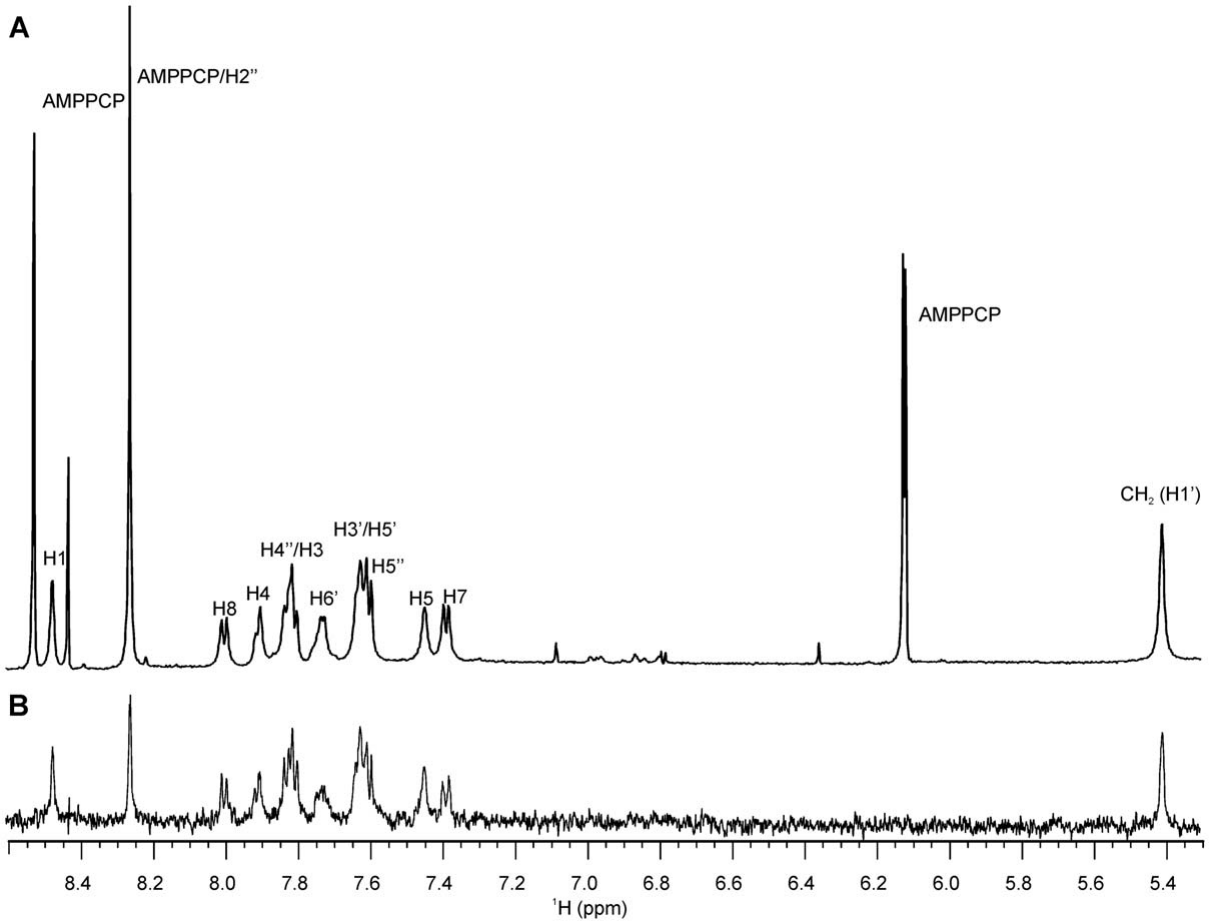
STD NMR spectrum of the compound 6b. (**A**) Reference spectrum. (**B**) STD spectrum. Note: The spectra intensities are not to scale.

Ligand epitope maps were obtained using STD NMR (Figure 8). Due to the non-uniform relaxation properties of the investigated ligands, a short saturation delay of 350 ms was used to avoid the effects of *T*_1_ relaxation times on the signal intensities. The *T*_1_ relaxation times range from 0.8 s in the alkyloxy chains to 7.9 s in the rigidified mimetics of D-Glu. Under short saturation conditions, the signal-to-noise ratio of the multiplet signals of cyclohexane ring protons and D-Glu β-protons is too low for any reliable determination of their STD effects. Therefore, for compounds **2a** and **2b** the STD effects of one entire proton-rich molecular segment are missing, and so the epitope maps of these two compounds have no significance.

**Figure 9:**
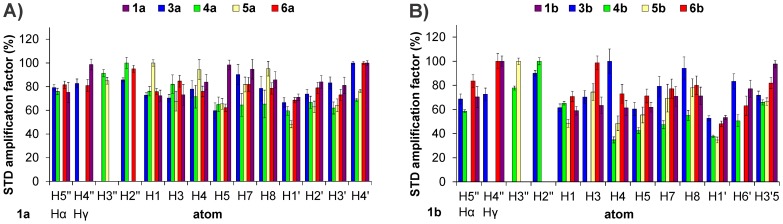
The relative STD amplification factors of individual protons. (**A**) The alkyloxy-substituted compounds and (**B**) the arylalkyloxy-substituted compounds (right). Some proton signals are missing due to overlap: the H3 proton overlaps with the phenyl mimetic ring proton in compound **4b**, H5” and H1” overlap in compounds **5a** and **5b**, the H6′ proton overlaps with H5” and H1” in compound **5b**, and the H2” proton overlaps with the AMPPCP signal in compounds **6a** and **6b**. The values are normalized to the intensities of the signals with the largest STD effects for each molecule. The Hα and Hβ at 1a and 1b indicate the protons of the D-Glu moiety.

These STD effects demonstrate that all ligand segments are involved in binding to MurD (Figure 9). In the C6 alkyloxy series (**1a–6a**) and in the C6 arylalkyloxy series (**1b–6b**), more uniform STD effects across the molecule are observed for the D-Glu derivatives (**1a**, **1b**) and for the *ortho, para*-substituted derivatives (**3a,** **3b**, **6a**, **6b**). For the other dicarboxyl-substituted derivatives, the relative strengths of the interactions of the C6 substituents are lower with regard to the rest of the molecule. In addition, for the derivatives **4b** and **5b**, the relative strengths of the naphthalene ring interactions are also significantly reduced. Obviously, the position of the dicarboxyl substitution patterns on the phenyl rings affects the binding interactions of all of the ligand segments.

The NMR data obtained by application of the STD, transferred NOESY and ^1^H/^13^C HSQC methods gave the following important findings about conformation, dynamics, binding site location, and binding interactions of the second-generation sulfonamide MurD inhibitors. All ligand segments are involved in binding to MurD, the ligand conformational dynamics is present despite the replacement of the D-Glu moiety with rigid mimetics, ligands are interacting with all three MurD domains, and they occupy the same binding site as the first-generation sulfonamide MurD inhibitors. The firm interactions in the uracil-binding site contribute significantly to the ligand potency. The notably increased inhibitory activity of **6b** in the more potent C6 arylalkyloxy series (**1b–6b**) can be mainly attributed to the better interactions with the central domain residues.

### Molecular dynamics at the receptor level

**Figure 10:**
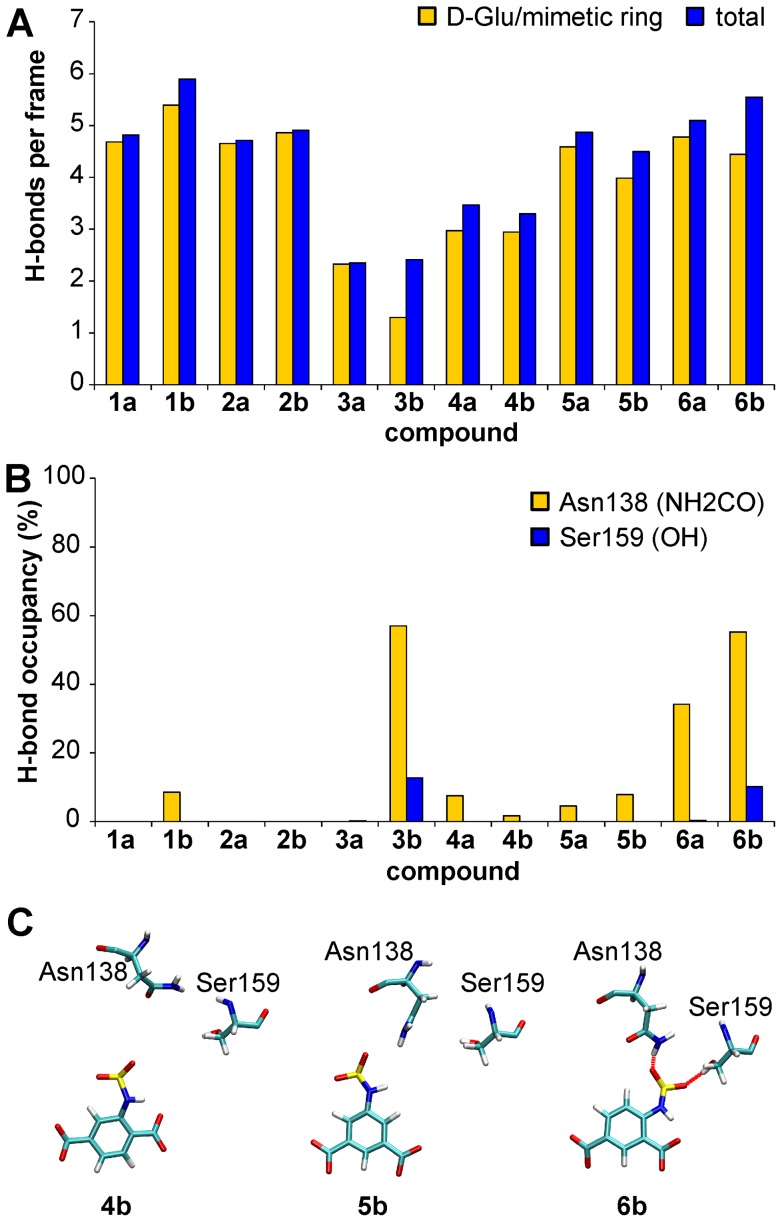
Intermolecular hydrogen bonds during the MD simulation. (**A**) Average number of hydrogen bonds per MD trajectory frame. (**B**) Occupancy of hydrogen bonds formed with the sulfonyl group of the inhibitors. (**C**) Representative snapshots from the MD trajectories of compounds **4b**, **5b**, and **6b** in complex with MurD, which show the favorable position of the sulfonamide group of **6b** for the formation of electrostatic interactions with Asn138 and Ser159 of MurD. For the sake of clarity, only the mimetic rings and the sulfonamide groups of the inhibitors are shown.

The complex relationships of ligand–MurD interactions revealed by the NMR data were further examined by unrestrained MD simulations of the inhibitor–MurD complexes. The rigidified inhibitors bearing carboxyl groups at *ortho, para* (**2a,** **2b**, **6a**, **6b**) and *meta, meta* (**5a**, **5b**) positions with regard to the sulfonamide moiety have the best hydrogen bonding networks with MurD (Figure 10A). They are comparable to those of their D-Glu analogs. The *ortho, meta*-substituted compounds (**4a** and **4b**) have significantly lower occupancies of hydrogen bonds in comparison with other ligands with dicarboxylic phenyl rings. A carboxyl group at the *para* position is clearly superior to a hydroxyl group (compounds **3a** and **3b**). The first carboxyl group at the *ortho* or *meta* positions with regard to the sulfonamide forms hydrogen bonds to the amine group of Lys348 and in some cases also to the hydroxyl group of Thr321. The second carboxyl group at the *para* or *meta* positions forms hydrogen bonds to the hydroxyl and amide groups of Ser415 and to some extent also to the amide group of Phe422 (Table S2, Dataset S3).

Ligands where their aromatic mimetic ring has a carboxyl group at the *ortho* position with regard to the sulfonamide moiety have a stable intramolecular hydrogen bond that forms a pseudo six-membered ring (Figure S5). However, the formation of this intramolecular hydrogen bond is not crucial for the overall ligand binding and conformational flexibility. Indeed, the position of the hydrogen-bond-forming substituent on the mimetic ring is more important. For example, compounds **5a** and **5b**, which lack internal hydrogen bonds, have significantly greater occupancies of the intermolecular hydrogen bonds than compounds **4a** and **4b**. The possible rotation of the phenyl ring mimetics of compounds **5a** and **5b** around the C6”–C3” axis is prevented by the stable hydrogen bonds of the symmetrically positioned dicarboxyl substituents (Figure S5).

The sulfonyl oxygens of compounds **6a**, **3b**, and **6b** form hydrogen bonds with the carboxamide group of Asn138 (Figure 10B and 10C). Occasionally, the sulfonyl oxygens of compounds **3b** and **6b** also form hydrogen bonds with the hydroxyl group of Ser159 (Figure 10B and 10C). The favorable position of the sulfonyl group for formation of electrostatic interactions with Asn138 and Ser159 depends on the position of the phenyl ring substituents (Figure 10B and 10C). The interactions of the *ortho*, *para*-substituted phenyl ring (compound **6b**) generate the most favorable position for the sulfonyl group relative to Asn138 and Ser159 (Figure 10C). These MD results confirm the importance of interactions with the central domain residues for the potency of investigated sulfonamide MurD inhibitors. Although compound **3b** has a significantly reduced hydrogen bonding network with the D-Glu-binding site (Figure 10A), it has comparable potency to compounds **4b** and **5b**, with compound **6b** as the most active. The activity of compound **2b** with *ortho, para*-substituted cyclohexane rings, which have an unfavorable configuration for formation of ligand interactions with Asn138 and Ser159, is also reduced.

The interactions of compounds **6a,** **3b**, and **6b** with the central domain residues Asn138 stabilize the position of their naphthalene rings with regard to the Phe161 ring. These rings are close enough to form π–π interactions. This is not observed for the other derivatives. The hydrophobic interactions between the naphthalene rings and Gly73 are observed in all cases here.

**Figure 11:**
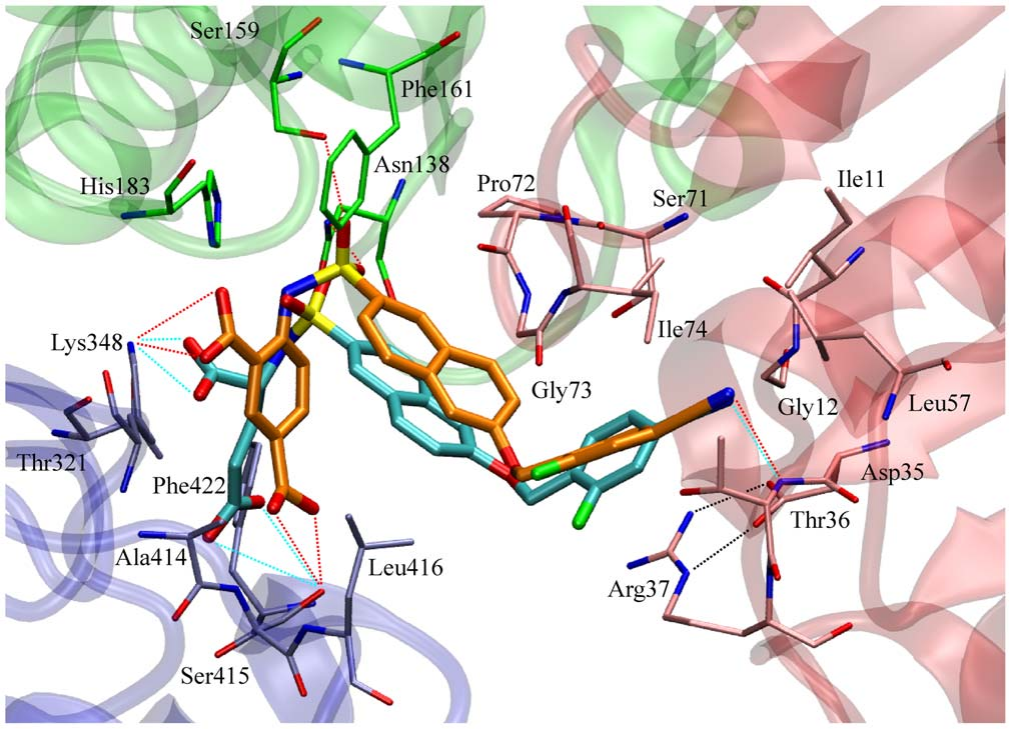
Comparison of the binding mode of the first and second generation sulfonamide inhibitors. The snapshot from the MD trajectory shows the binding mode of the compound **6b** (in orange). The compound **1b** (in cyan) is superimposed (crystal structure PDB code 2VTD [8]). The *N*-terminal domain, central domain, and *C*-terminal domains are colored in red, green, and blue respectively. The MurD structures were aligned using Visual Molecular Dynamics (VMD) program [42].

Most of the ligands with the *p*-cyano-2-fluorobenzyloxy substituent (**1b**, **3b–6b**) form very stable hydrogen bonds with the amide group of Thr36 in the uracil binding pocket and have π–π interactions with the Asp35–Arg37 salt bridge. Additional stabilization is achieved through stable cation-π interactions between their phenyl rings and the positively charged guanidino group of Arg37. The same interactions are also observed in the co-crystal structure of compound **1b** (Figure 11).

The introduction of substituted benzoic acid derivatives as glutamic acid mimetics in the second-generation sulfonamide inhibitors allows π–π stacking interactions with the Phe422 phenyl ring (Figure 11). This might contribute to increased binding affinities compared to the D-Glu-containing compounds. Another important difference between the binding modes of the most potent compound from the first-generation **1b** and of the most potent compound from the second-generation **6b** that could contribute to the 10-fold difference in their inhibitory activities lies in interactions with the central domain residues (Figure 11). Only indirect interactions of the ligand sulfonyl group across the water molecule with the residues Asn138 and Ser159 are observed in the crystal structure of the **1b**–MurD complex [8]. The MD simulations show that the direct hydrogen bond of compound **1b** with Asn138 is formed much less frequently compared to the case of compound **6b** (Figure 10B). These observations are supported by NMR data. The CSPs' patterns reveal a significantly increased effect of compound **6b** on the central domain signals with regard to the compound **1b** (Figure 5).

**Figure 12:**
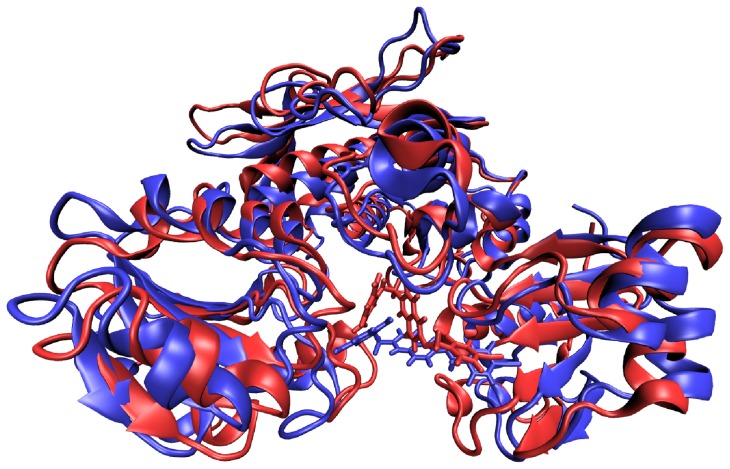
MurD domain flexibility. Snapshot of the most “closed” (red) and the most “open” (blue) conformations during the simulation of the **4b**–MurD complex. The trajectory frames were aligned using the root mean square deviation trajectory tool extension in VMD [42].

The MD data reveal complex dynamic behaviors of these ligand–MurD complexes and show that these influence the ligand–enzyme contacts. As well as the rotation of ligand segments at the MurD binding site, as revealed by transferred NOESY, slight opening/closing movements of the protein domains are seen in MD trajectories (Figure 12).

Movements of protein domains can adversely affect ligand binding through effects on the conformation and flexibility of the bound ligand, the stability of the ligand–enzyme interactions, and the binding-site adaptability. These movements should not be confused with the open and closed conformations of the MurD protein that have been reported in the literature, where the *C*-terminal domain has a drastically different position [6]. The most pronounced fluctuations are evident from the distances between the geometric centers of the *C-*terminal and *N-*terminal domains. The variations between the minimal and maximal distances can be up to 5.5 Å (Figure S6). An additional, longer (15 ns) MD run of selected ligands **6a** and **6b** clearly indicate that the fluctuations of distance between these geometric centers are less pronounced when the most potent inhibitor **6b** is bound to MurD (Figure S7). This might be the consequence of better binding interactions that tend to hold the domains together.

Visual inspection of the trajectories reveals that the movements of the *C*-terminal and *N*-terminal domains have important roles in ligand binding. Sulfonamide inhibitors span from the *N-*terminal domain to the *C-*terminal domain. Opening movements tend to weaken the interactions either with the uracil binding pocket or with the D-Glu binding site. The C6-alkyloxy-substituted compounds (**1a–6a**) generally have weaker interactions with the uracil binding pocket compared to the C6-arylalkyloxy-substituted compounds (**1b–6b**). Consequently, the alkyl chains can even be pulled out of the uracil binding pocket during domain movement. The C6-alkyloxy-substituted compounds are also shorter than the C6-arylalkyloxy-substituted compounds. Therefore, the alkyl chain has more freedom to move in the uracil binding pocket. This is accompanied by conformational changes of the ligand. During domain movement, a rotation of the mimetic ring for compounds **3a**, **4a**, and **5a** is observed around the hinges formed by the carboxyl groups that switches their conformation between extended and bent form (Figure S8).

In general, rotation of the D-Glu mimetic around the C3”–C6” axis is not observed. Therefore, the mutually exclusive NOEs between H1–H5′′ and H3-H5′′ are a consequence of the naphthalene ring rotations, just as for the D-Glu analogs. For several derivatives, naphthalene ring rotations around its C2–C6 axis are observed during MD simulations. During pronounced reorientations of the naphthalene ring, notable changes in H1–H5′′ and H3–H5′′ distances appear (Dataset S3). Generally, a particular orientation of this ring corresponds to a proximity of H1–H5′′ or of H3–H5′′ protons (Dataset S3).

The specific determination of binding interactions of the sulfonamide MurD inhibitors and the observed dynamic behavior of ligand-MurD complexes are in agreement with the crucial NMR experimental findings about the binding mode of these inhibitors. (i) The rigid D-Glu mimetics of second generation sulfonamide inhibitors form stable electrostatic interactions with the D-Glu-binding site, which is supported by their large effects on the CPSs of methyl groups near the D-Glu-binding site. (ii) The C6 arylalkyloxy substituents are stabilized in the uracil-binding pocket with a number of stable electrostatic and hydrophobic interactions. This is in agreement with their pronounced effects on the CSPs of methyl groups near the uracil binding site. (iii) The C6 alkyloxy substituents are flexible in the uracil-binding site, forming weaker hydrophobic interactions; the CSPs of methyl groups near the uracil binding site are significantly lower. (iv) The naphthalene ring rotations are supported by the NOE patterns of bound ligands. (v) The type of substitution of rigid D-Glu mimetic significantly effects the electrostatic interactions of the sulfonamide group with the central domain. This is supported by the pronounced effects of **6b** on the CPSs belonging to the central domain residues.

## Conclusions

The ^1^H/^13^C HSQC on the selectively labeled MurD protein provides an effective tool for determination of the binding sites of novel inhibitors. These second-generation sulfonamide inhibitors have similar binding modes as those of the first generation [7], [8]. The NMR data clearly show better binding of the C6-arylalkyloxy-substituted compounds (**1b,** **2b–6b**) compared to the C6-alkyloxy-substituted compounds (**1a**, **2a–6a**) and indicate the importance of stable interactions with the uracil binding site.

The introduction of rigid mimetics of D-Glu resulted in increased potencies for these inhibitors. The *ortho, para*-carboxyl-disubstituted phenyl ring (**6a, 6b**) is the most suitable moiety to mimic D-Glu. MD simulations show that this is due to the more favorable position of the sulfonyl group with regard to Asn138 and Ser159. NMR CSP data also show that the most active *ortho, para*-disubstituted C6-arylalkyloxy derivative (**6b)** has the best contact with the central domain residues. The *meta, meta* substitutions (**5a, 5b**) result in reduced average numbers of ligand-enzyme hydrogen bonds, while the *ortho, meta*-carboxyl-disubstituted phenyl rings (**4a**, **4b)** are the least suitable D-Glu mimetics for the overall occupancies of the hydrogen bonds. The replacement of the carboxyl groups with hydroxyl groups at the *para* position (**3a**, **3b)** significantly reduces the number of hydrogen bonds, while the replacement of the phenyl rings with cyclohexane rings (**2a**, **2b**) prevents the formation of electrostatic interactions with Asn138 and Ser159 and π–π interactions with Phe422.

MurD conformational changes have to date been given insufficient attention in the process of MurD inhibitor optimization. MD simulations show the complex dynamic behavior of these MurD–inhibitor complexes, where the interactions are affected both by movements of the protein domains and by the flexibility of the ligand. The differing degrees of conformational flexibility of the ligands were also predicted on the basis of the NOE patterns. The sulfonamide inhibitors studied span from the *C-*terminal domain to the *N-*terminal domain and also interact with the central domain. The distances between the *C-*terminal and *N-*terminal domains fluctuate. Therefore, the bound ligands are exposed to stretching forces that tend to pull either the D-Glu mimetic part or the C6 substituent out of the binding site. Stronger interactions in one domain tend to weaken the interactions in the other domains. This needs to be considered in the optimization of these sulfonamide inhibitors through the design of new compounds that have improved interactions not only with one but with both the *C*-terminal and *N*-terminal domains of MurD. Our data also suggest that inhibitors that can span from the *C*-terminal domain to the *N*-terminal domain should not be highly rigid to allow them to adapt to the conformational changes of the MurD protein. Such compounds might also benefit from having slightly longer linkers between the naphthalene rings and the aromatic rings of the C6 substituent. These data represent upgraded knowledge that will now be useful for the rational structure-based design of new improved MurD inhibitors.

## Materials and Methods

### Isolation and purification of the ^13^C selectively labeled MurD enzyme from *E. coli*

The ^13^C selectively labeled MurD protein was overexpressed according to a modified procedure that has been previously described [21] and purified according to a procedure previously used in our laboratory for the unlabeled MurD protein [17]. *E. coli* BL21(DE3)pLysS cells that were freshly transformed with the pABD16 plasmid [22] were grown overnight at 37°C in 10 mL Luria-Bertani rich growth medium containing ampicillin (100 mg/L). The cells were centrifuged down and resuspended in 50 mL M9 minimal medium containing 6.5 g/L Na_2_HPO_4_, 3 g/L KH_2_PO_4_, 0.5 g/L NaCl, 1 g/L NH_4_Cl, 3 g/L D-glucose, 120 mg/L MgSO_4_, 11 mg/L CaCl_2_, 10 mg/L thiamine, 10 mg/L biotin, and 100 mg/L ampicillin. Following being grown to an A_600nm_ of 0.1, the cells were centrifuged down again and resuspended in 200 mL ^15^N-labeled M9 medium. At an A_600nm_ of about 0.5, the cells were divided into two flasks containing 400 mL ^15^N-labeled M9 medium. At an A_600nm_ of 0.25, α-ketobutyrate (99% methyl ^13^C) and α-ketoisovalerate (99% dimethyl ^13^C_2_) solutions were added, making final concentrations of 70 mg/L and 120 mg/L respectively. Cell growth was then continued for 1 h. Expression was induced by the addition of β-D-thiogalactopyranoside, to a final concentration of 1 mM. Cell growth was continued for 8 h. The cells were then harvested and resuspended in 20 mM potassium phosphate buffer, pH 7.2, containing 1 mM dithiothreitol (DTT). The cells were disrupted by sonication using a Cole Parmer ultrasonic processor. The suspension was centrifuged, and the pellet was discarded. Pre-equilibrated Ni^2+^-nitrilotriacetate-agarose polymer (Ni^2+^-NTA) was added to the supernatant, followed by an incubation on a multi-function tube rotator for 1 h. The suspension was centrifuged to recover the polymer. The protein was eluted from the polymer with increasing concentrations of imidazole (20, 40, 100 mM) in 50 mM potassium phosphate buffer, pH 8.0, containing 200 mM KCl and 1 mM DTT. The fractions containing the MurD protein were collected and checked by SDS-PAGE. The fractions with a satisfactory degree of purity of the MurD protein were combined and concentrated using an Amicon Ultra 10K NMWL concentrator. The samples were then dialyzed against a 20 mM HEPES buffer, pH 7.2, containing 7 mM (NH_4_)_2_SO_4_, 3.5 mM MgCl_2_, and 0.3 mM DTT. Protein concentrations were determined by measuring the A_280_ on a Nanodrop ND-1000 spectrophotometer. The yield was about 20 mg of MurD protein per 1 L of growth medium. Glycerol was added to the MurD protein solution (10%, v/v), which was then frozen at −24°C. The MurD activity was checked using a Biomol green assay [23].

### NMR spectroscopy

NMR spectra were recorded at 25°C on a Varian DirectDrive 800 MHz spectrometer equipped with a cryoprobe. The pulse sequences provided by the Varian BioPack library were used.

The NMR samples for STD and transferred NOESY were prepared in a buffer containing D_2_O, 20 mM Tris-*d*_11_, 7 mM (ND_4_)_2_SO_4_-*d*_8_, 3.5 mM MgCl_2_, 0.3 mM DTT-*d*_10_, and 0.4 mM AMPPCP, pD 7.8. Dimethyl sulfoxide (DMSO)-*d*_6_ (12%, v/v) was added to enhance the solubility of the ligands. Sodium 2,2-dimethyl-2-silapentane-5-sulfonate (0.1 mM) was used as the internal standard. The protein concentration used was 0.006 mM, and the ligand concentrations were 0.3 mM and 0.6 mM for transferred NOESY and STD respectively. The ligands were prepared according to Sosič et al. [11] and Humljan et al. [8]. Elemental analysis was used to determine the purities of the ligands, which were always ≥95%.

The NMR samples for the ^1^H/^13^C-HSQC were prepared in a buffer containing H_2_O, 20 mM HEPES, 7 mM (NH_4_)_2_SO_4_, 3.5 mM MgCl_2_, 0.3 mM DTT, and 2 mM AMPPCP, pH 7.2. DMSO-*d*_6_ (10%, v/v) was added to enhance the solubility of the ligands. The concentration of MurD selectively labeled with ^13^C at the methyl groups of Ile (δ1 only), Val, and Leu was 0.07 mM. The MurD protein was titrated against the ligands with the ligand/MurD molar ratios of 0.5, 1, 2, 5, and 10. The concentrations of DMSO-*d*_6_ in the NMR tube varied from 10% (v/v) to 12% (v/v) due to the addition of the ligands in DMSO-*d*_6_ solution. The effect of DMSO-*d*_6_ on the protein was investigated at 5%, 10% and 12% (v/v) of DMSO-*d*_6._ The CSPs of signals due to the addition of DMSO-*d*_6_ are significantly lower in comparison to the ligand binding effect, and the overall appearance of HSQC spectra does not change, indicating that no significant changes in the protein structure appear (Dataset S4). The 5% dimethyl sulfoxide is generally used during MurD activity tests [24]. The variation of DMSO-*d*_6_ from 5% to 10% (v/v) affects the CSPs below 0.04 ppm (Dataset S4), while the variation of DMSO-*d*_6_ from 10% to 12% (v/v) affects the CSPs below 0.02 ppm (Dataset S4).

STD ligand epitope mapping [25], [26] was performed with an 8389 Hz spectral width, with 16384 data points, a saturation time of 350 ms, a relaxation delay of 11.35s, and 3000 to 8000 scans. The spectra were recorded at a protein/ligand ratio of 1∶100. Selective saturation was achieved by a train of 50 ms long Gauss-shaped pulses, separated by 1 ms delays. Water was suppressed via excitation sculpting [27], [28]. The on-resonance selective saturation of MurD was applied at 0.21 ppm. The off-resonance irradiation was applied at 30 ppm for the reference spectrum. Subtraction of the on-resonance and off-resonance spectra was performed internally via phase cycling. The spectra were zero-filled twice and apodized by an exponential line-broadening function of 1 Hz. Errors in the STD amplification factor were estimated according to the formula: STD amplification factor absolute error  =  STD amplification factor × ((N_STD_/I_STD_^ 2^ + N_REF_/I_REF_^ 2^)^1/2^. N_STD_ and N_REF_ are noise levels in STD and reference spectra; I_STD_ and I_REF_ are signal intensities in STD and reference spectra [29].

The transferred NOESY [30], [31] spectra were acquired at a protein/ligand ratio of 1∶45, with an 8389 Hz spectral width, with 4096 data points in t_2_, 32–48 scans, 256–356 complex points in t_1_, a mixing time of 250 ms, and a relaxation delay of 1.5 s. The residual water signal was suppressed using excitation sculpting [27], [28], and adiabatic pulses [32] were applied for suppression of the zero quantum artifacts during the mixing time. A T_1ρ_ filter of 30 ms was used to eliminate the background protein resonance. Spectra were processed and analyzed with the FELIX 2007 software package from Felix NMR Inc. The spectra were zero-filled twice and apodized with a squared sine bell function shifted by π/2 in both dimensions.

The ^1^H/^13^C-HSQC [33] spectra were acquired with 1024 data points in t_2_, 32 scans, 64 complex points in t_1_, and a relaxation delay of 1 s. The ^1^H and ^13^C sweep widths were at 9470 Hz and 3340 Hz respectively. The spectra were processed using the NMRPipe software [34] and analyzed using the Sparky software [35]. The spectra were zero-filled and apodized with a squared sine bell function shifted by π/2 in both dimensions, using linear prediction of the data in the incremented dimension. The combined chemical shift perturbations Δδ were calculated from the ^1^H and ^13^C chemical shift changes using the equation: Δδ  =  ((Δδ^1^H)^2^ + (0.252 × Δδ^13^C)^2^)^1/2^ [18]. ^1^H chemical shifts for the MurD protein were predicted using the software SHIFTS 4.1.1, which is available online (http://casegroup.rutgers.edu/qshifts/qshifts.htm). PDB entries 2JFF [7], 2VTD [8], and 2XPC [11] were used as input structures.

### Molecular modeling

The calculations were performed on Beowulf-type CROW clusters [36] at the National Institute of Chemistry in Ljubljana, Slovenia, using the CHARMM molecular modeling suite [37] (developmental version 36a1). The MurD protein coordinates were obtained from the PDB entry 2VTD [8]. Force-field parameters for MurD were obtained from the CHARMM parameter and topology files (version 27) for proteins [38],[39]. The parameters for the ligands were obtained from the CHARMM General Force Field parameter and topology files for drug-like molecules [40]. Autodock4 [41] was used to determine the initial structures. Lamarckian genetic algorithms with default values were used for the docking. The structures with the most favorable interactions were chosen for the initial positions in the MD and minimized again using CHARMM. The compounds alone were subjected to energy minimization using the adopted basis Newton–Raphson method, with 1000 steps. The structures were then hydrated by immersion in a cubic box (86 Å ×86 Å ×86 Å) of water molecules. Deletion of the water molecules that overlapped with the MurD protein resulted in a system with approximately 20000 TIP3 water molecules. The entire system was again subjected to energy minimization using the adopted basis Newton–Raphson method, with 1000 steps. The MD simulations were run at 1 fs time steps for 2 ns. The constant pressure and temperature (CPT) ensemble was used in all of the calculations, with 1 bar pressure and 300 K temperature. The electrostatic interactions were computed using the particle-mesh Ewald method. The systems were equilibrated after 50–100 ps of MD simulation; therefore, the first 100 ps of the MD trajectories were not considered in the analysis. Additionally, two 15 ns long MD simulations were run for compounds **6a** and **6b**.

## Supporting Information

Figure S1 **Representative ^1^H/^13^C HSQC NMR spectrum of methyl resonances (Ile δ1, Val, Leu) of MurD.**
(DOC)

Figure S2 **Stereograms of Ile (δ1), Val, and Leu methyl groups in MurD protein.**
(DOC)

Figure S3 **The CSP patterns of the ^13^C labeled methyl groups upon binding AMPPCP.**
(DOC)

Figure S4 **Distances of Ile (δ1), Val, and Leu groups to the nearest ligand atom.**
(DOC)

Figure S5 **Intramolecular hydrogen bond and mimetic ring rotation.**
(DOC)

Figure S6 **Distances between geometric centers of the** ***C*** **-terminal and** ***N*** **-terminal domains during 2 ns MD simulations.**
(DOC)

Figure S7 **Distances between geometric centers of the** ***C*** **-terminal and** ***N*** **-terminal domains during 15 ns MD simulations.**
(DOC)

Figure S8 **Representation of the mimetic ring rotation around the hinges formed by the carboxyl groups.**
(DOC)

Table S1 **^1^H/^13^C HSQC chemical shifts of the MurD enzyme in the presence of AMPPCP (protein/AMPPCP ratio 1∶20).**
(DOC)

Table S2 **Hydrogen bonds formed on the D-Glu mimetic ring during MD simulations.**
(DOC)

Dataset S1 **Overlays of ^1^H/^13^C HSQC NMR spectra in absence and presence of the ligands.**
(DOC)

Dataset S2 **Chemical shifts predicted in program SHIFTS 4.1.1.**
(DOC)

Dataset S3 **MD analysis data.**
(DOC)

Dataset S4 **DMSO-** ***d*** **_6_ effect on the protein.**
(DOC)
