# Supplementary material for: The Binding Mode of Second-Generation Sulfonamide Inhibitors of MurD: Clues for Rational Design of Potent MurD Inhibitors
Source: PLoS One. 2012 Dec 20;7(12):e52817. doi: 10.1371/journal.pone.0052817 (PMC3527612; doi:10.1371/journal.pone.0052817)
Supplement: Figure S1 — Representative 1H/13C HSQC NMR spectrum of methyl resonances (Ile δ1, Val, Leu) of MurD. (DOC) [file pone.0052817.s001.doc]

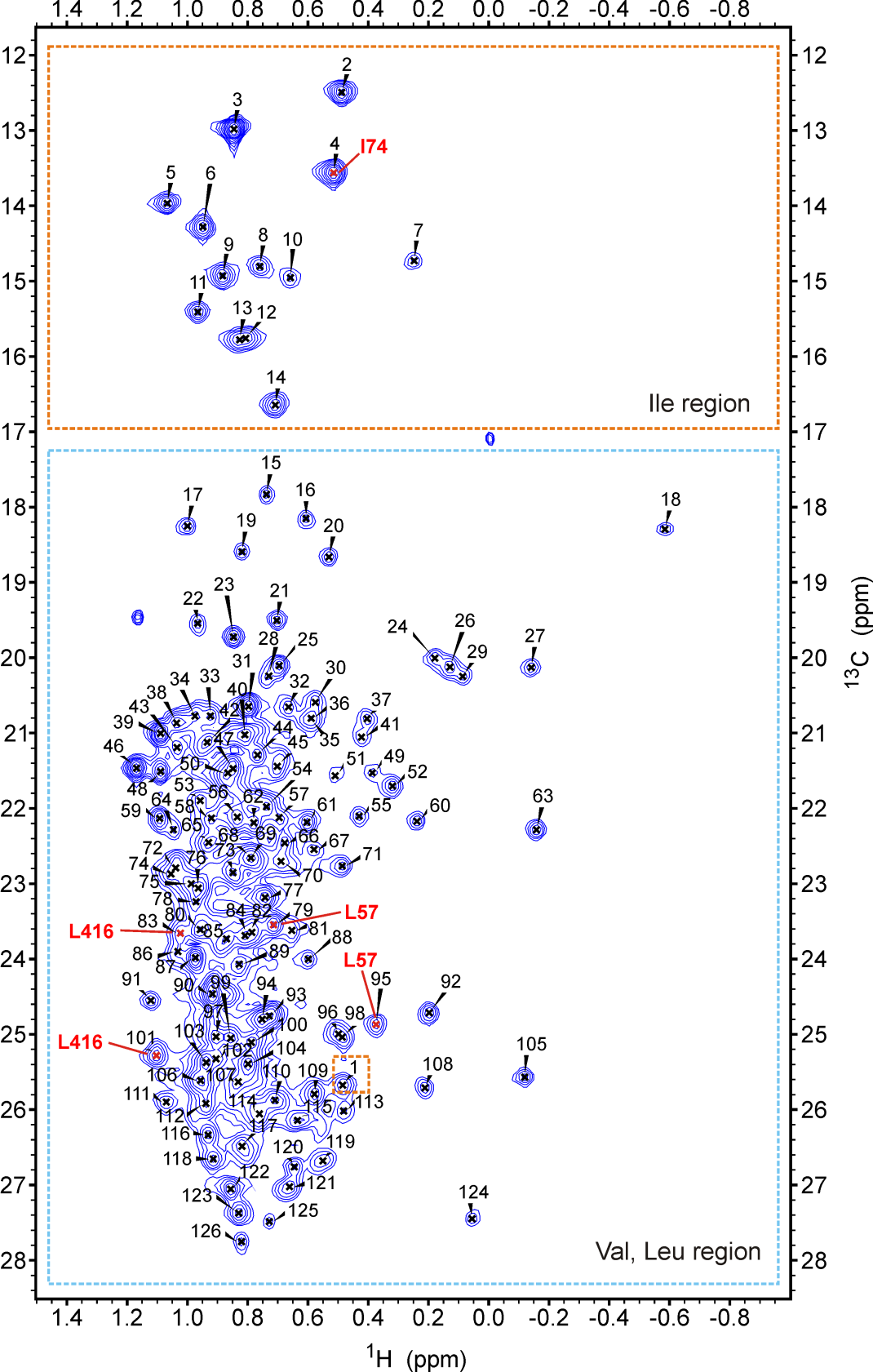


Figure S1. Representative ^1^H/^13^C HSQC NMR spectrum of methyl resonances (Ile δ1, Val, Leu) of MurD. The proposed Ile and Val/Leu regions are marked with orange and light blue boxes respectively. The proposed assignments of the crucial methyl groups are marked in red. One Ile signal was deliberately folded into the Leu region. Note that the numbering of resonances does not correspond to the MurD residue numbers. The resonances are numbered according to the positions of the signals in the ^13^C dimension of the ^1^H/^13^C HSQC spectrum, starting from the most up-field position.
