# Supplementary material for: The Binding Mode of Second-Generation Sulfonamide Inhibitors of MurD: Clues for Rational Design of Potent MurD Inhibitors
Source: PLoS One. 2012 Dec 20;7(12):e52817. doi: 10.1371/journal.pone.0052817 (PMC3527612; doi:10.1371/journal.pone.0052817)
Supplement: Figure S2 — Stereograms of Ile (δ1), Val, and Leu methyl groups in MurD protein. (DOC) [file pone.0052817.s002.doc]

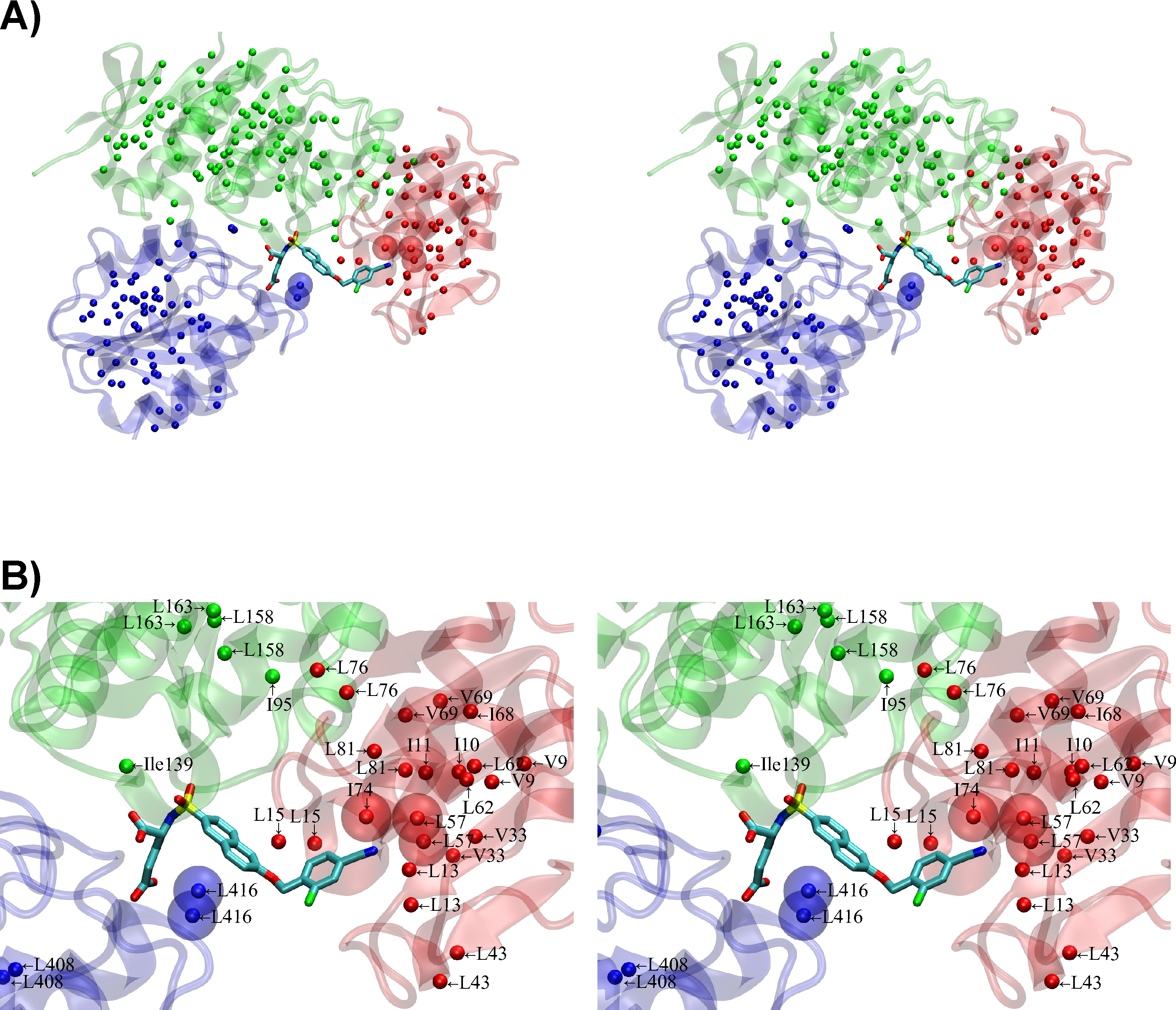


**Figure S2:** **Stereograms of Ile (δ1), Val, and Leu methyl groups in MurD protein. (A)** MurD protein with bound *N*-(6-(4-cyano-2-fluorobenzyloxy)naphthalene-2-sulfonamido)-d-glutamic acid (PDB entry 2VTD [8]). Ile (δ1), Val, and Leu methyl groups are represented as spheres. N-terminal domain is colored in red, central domain is colored in green, and C-terminal domain is colored in blue. **(B)** Close-up view of MurD binding site (PDB entry 2VTD [8]) with bound *N*-(6-(4-cyano-2-fluorobenzyloxy)naphthalene-2-sulfonamido)-d-glutamic acid. Only the methyl groups within 12 Å of the ligand are shown. Methyl groups in the range of 5 Å are marked as transparent Van der Waals spheres.
