# Supplementary material for: The Binding Mode of Second-Generation Sulfonamide Inhibitors of MurD: Clues for Rational Design of Potent MurD Inhibitors
Source: PLoS One. 2012 Dec 20;7(12):e52817. doi: 10.1371/journal.pone.0052817 (PMC3527612; doi:10.1371/journal.pone.0052817)
Supplement: Figure S3 — The CSP patterns of the 13C labeled methyl groups upon binding AMPPCP. (DOC) [file pone.0052817.s003.doc]

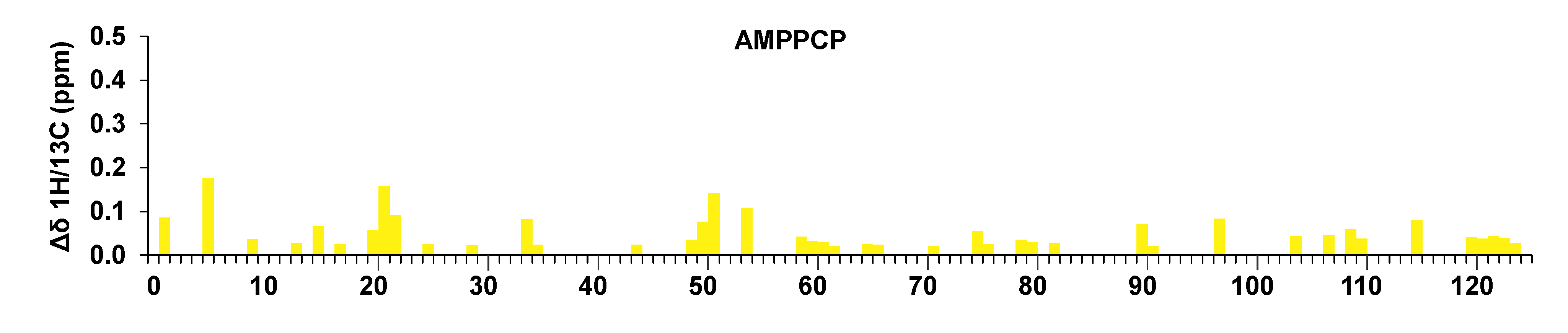


Figure S3. The CSP patterns of the ^13^C labeled methyl groups upon binding of AMPPCP. Note that the numbering of CSPs does not correspond to the MurD residue numbers. The CSPs are numbered according to the positions of the signals in the ^13^C dimension of the ^1^H/^13^C HSQC spectrum, starting from the most up-field position. Only the values above the threshold of 0.02 ppm are shown, to neglect the effects of the 2% variation in the DMSO-*d*_6_ concentration at ligand titration.
