# Supplementary material for: The Binding Mode of Second-Generation Sulfonamide Inhibitors of MurD: Clues for Rational Design of Potent MurD Inhibitors
Source: PLoS One. 2012 Dec 20;7(12):e52817. doi: 10.1371/journal.pone.0052817 (PMC3527612; doi:10.1371/journal.pone.0052817)
Supplement: Figure S4 — Distances of Ile (δ1), Val, and Leu groups to the nearest ligand atom. (DOC) [file pone.0052817.s004.doc]

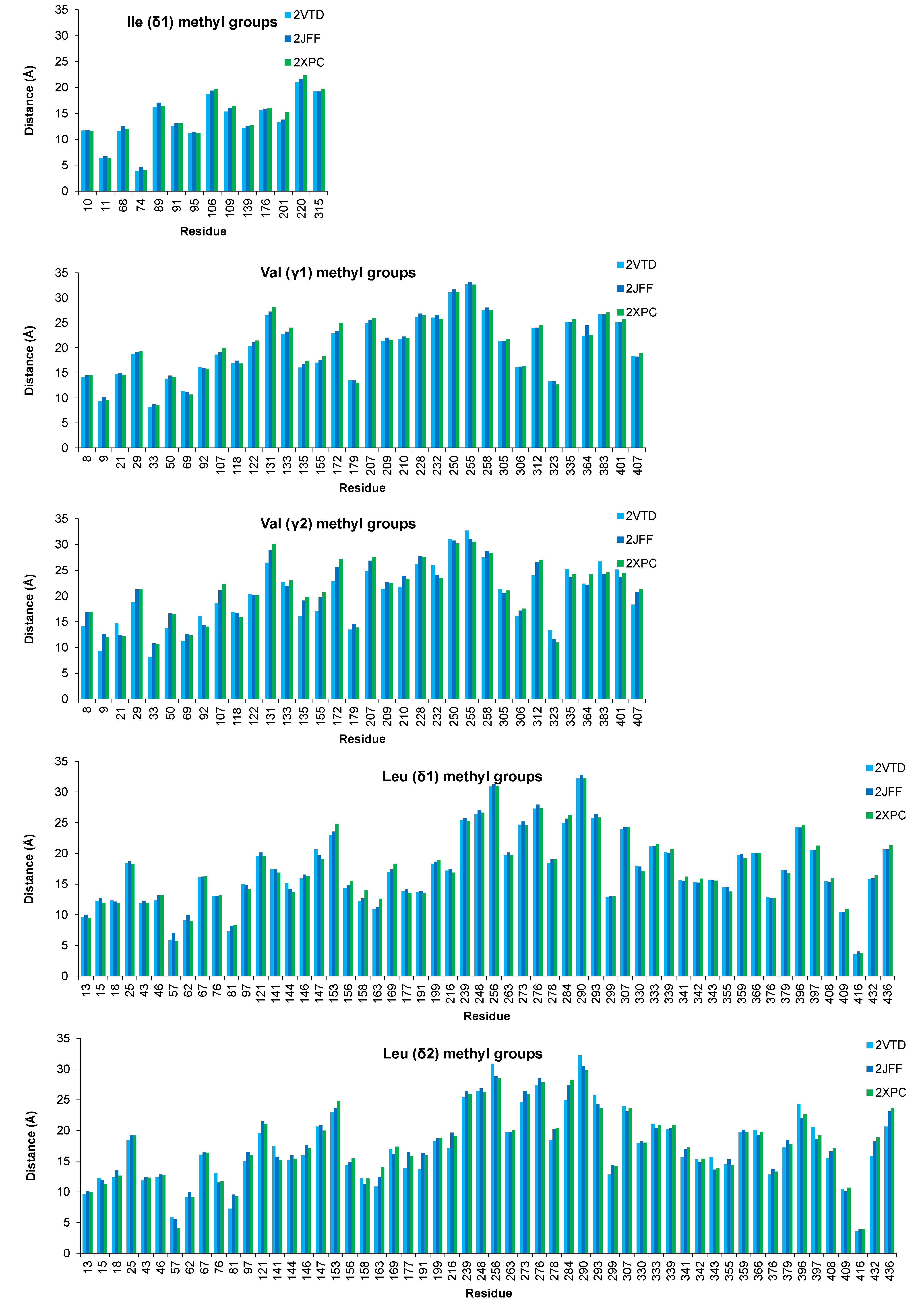


**Figure S4. Distances of Ile (δ1), Val, and Leu groups to the nearest ligand atom.** Crystal structures 2VTD [8], 2JFF [7] and 2XPC [11] were used. Distances were measured using the VMD program [42].
