# Supplementary material for: The Binding Mode of Second-Generation Sulfonamide Inhibitors of MurD: Clues for Rational Design of Potent MurD Inhibitors
Source: PLoS One. 2012 Dec 20;7(12):e52817. doi: 10.1371/journal.pone.0052817 (PMC3527612; doi:10.1371/journal.pone.0052817)
Supplement: Figure S5 — Intramolecular hydrogen bond and mimetic ring rotation. (DOC) [file pone.0052817.s005.doc]

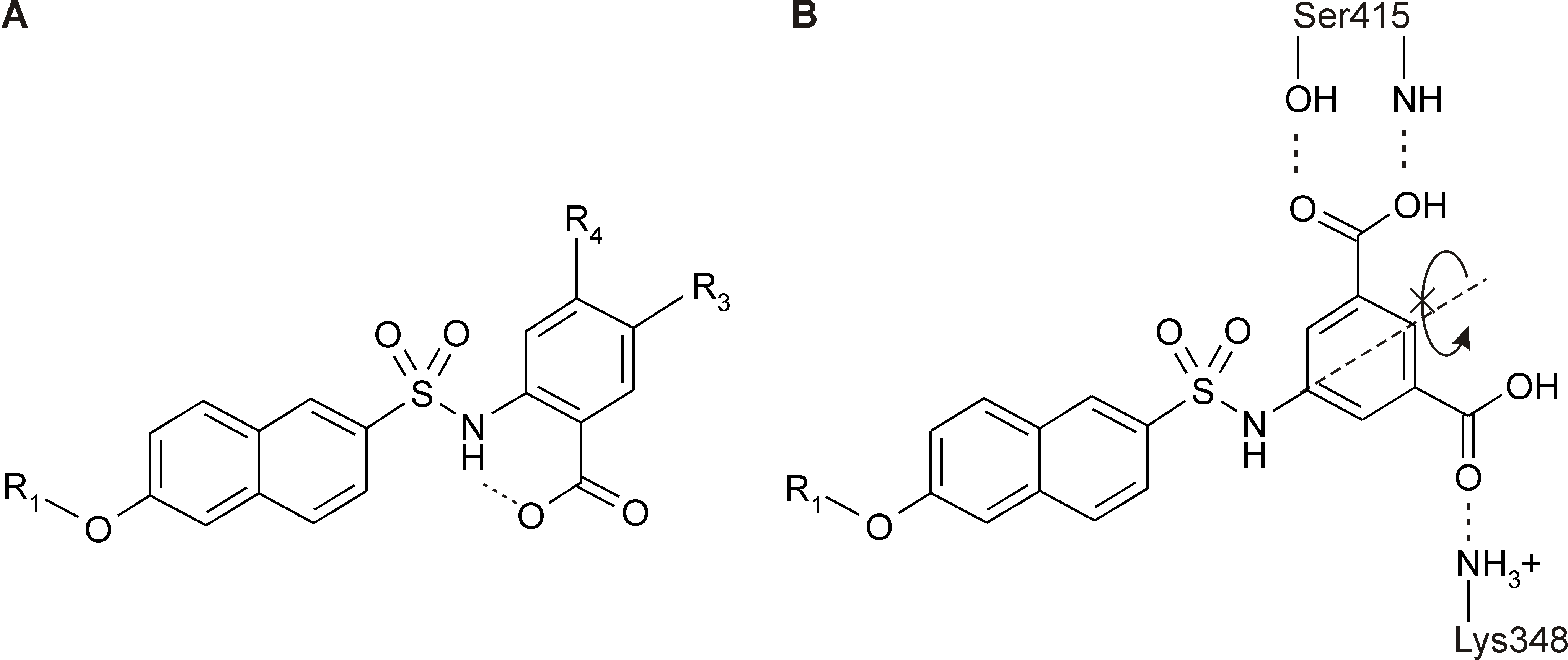


Figure S5. Intramolecular hydrogen bond and mimetic ring rotation. (A) Schematic presentation of the intramolecular hydrogen bond in the *ortho-*substituted compounds. (B) Schematic presentation of the *meta*, *meta*-substituted phenyl ring, and hindered rotation around the C6”-C3” axis. R_1_ indicates the C6 alkyloxy or arylalkyloxy substituents, and R_3_ and R_4_ indicate the carboxyl or hydroxyl phenyl ring substituents.
