# Supplementary material for: The Binding Mode of Second-Generation Sulfonamide Inhibitors of MurD: Clues for Rational Design of Potent MurD Inhibitors
Source: PLoS One. 2012 Dec 20;7(12):e52817. doi: 10.1371/journal.pone.0052817 (PMC3527612; doi:10.1371/journal.pone.0052817)
Supplement: Figure S6 — Distances between geometric centers of the C -terminal and N -terminal domains during 2 ns MD simulations. (DOC) [file pone.0052817.s006.doc]

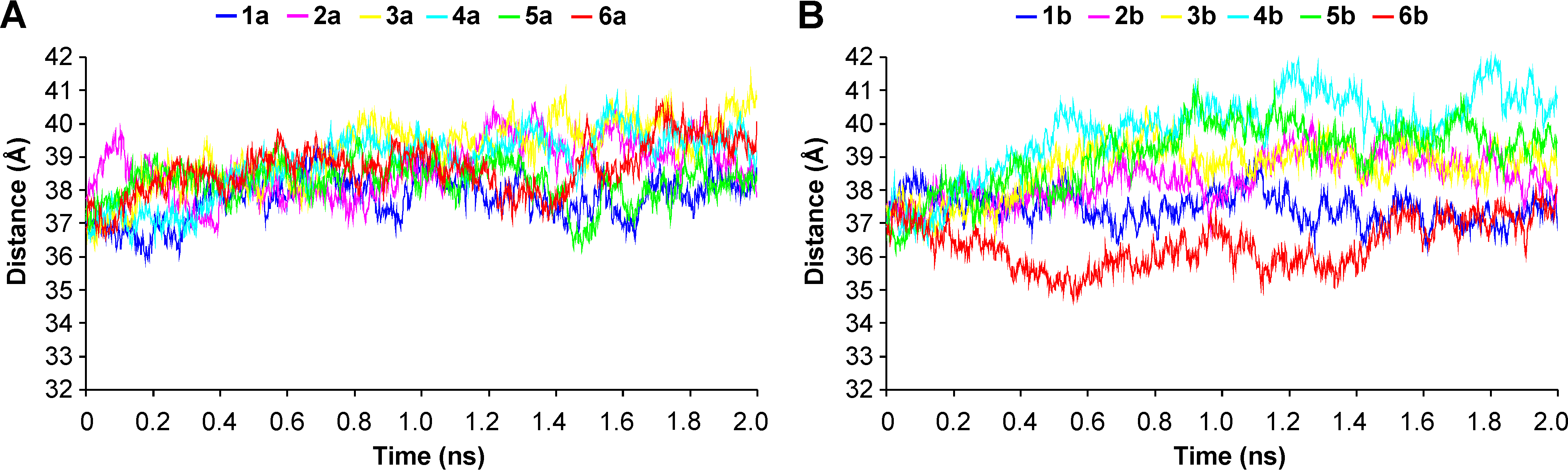


Figure S6. Distances between geometric centers of the *C*-terminal and *N*-terminal domains during 2 ns MD simulations. (A) Distances for alkyloxy substituted compounds, (B) Distances for arylalkyloxy substituted compounds.
