# Supplementary material for: The Binding Mode of Second-Generation Sulfonamide Inhibitors of MurD: Clues for Rational Design of Potent MurD Inhibitors
Source: PLoS One. 2012 Dec 20;7(12):e52817. doi: 10.1371/journal.pone.0052817 (PMC3527612; doi:10.1371/journal.pone.0052817)
Supplement: Figure S8 — Representation of the mimetic ring rotation around the hinges formed by the carboxyl groups. (DOC) [file pone.0052817.s008.doc]

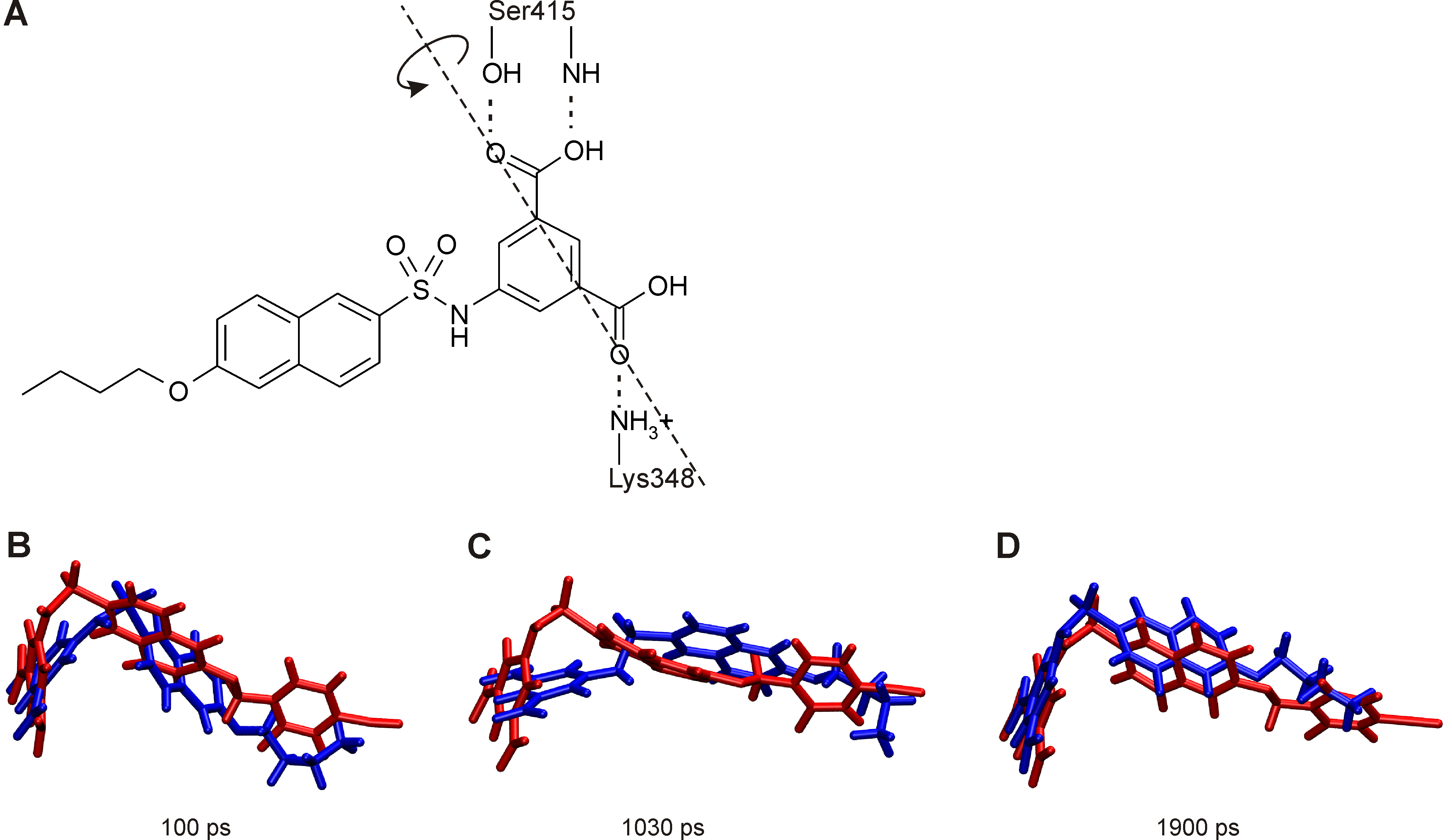


Figure S8. Representation of the mimetic ring rotation around the hinges formed by the carboxyl groups. (A) Schematic representation. (B) The snapshots from 100 ps, (C) the snapshots from 1030 ps, and (D) the snapshots from 1900 ps showing the rotation of the mimetic ring. Compound 5a is blue; compound 5b is red.
