# Supplementary material for: The Binding Mode of Second-Generation Sulfonamide Inhibitors of MurD: Clues for Rational Design of Potent MurD Inhibitors
Source: PLoS One. 2012 Dec 20;7(12):e52817. doi: 10.1371/journal.pone.0052817 (PMC3527612; doi:10.1371/journal.pone.0052817)
Supplement: Table S1 — 1H/13C HSQC chemical shifts of the MurD enzyme in the presence of AMPPCP (protein/AMPPCP ratio 1∶20). (DOC) [file pone.0052817.s009.doc]

**Table S1: ^1^H/^13^C HSQC chemical shifts of the MurD enzyme in the presence of AMPPCP (enzyme:AMPPCP ratio 1:20).**

| **Resonance^a^** | **w1 (ppm)** | **w2 (ppm)** | **Resonance^a^** | **w1 (ppm)** | **w2 (ppm)** | **Resonance^a^** | **w1 (ppm)** | **w2 (ppm)** |
| --- | --- | --- | --- | --- | --- | --- | --- | --- |
| 1 | 8.57 | 0.48 | 43 | 21.19 | 1.03 | 85 | 23.73 | 0.87 |
| 2 | 12.50 | 0.49 | 44 | 21.29 | 0.77 | 86 | 23.91 | 1.03 |
| 3 | 12.98 | 0.85 | 45 | 21.44 | 0.70 | 87 | 23.99 | 0.97 |
| 4 | 13.57 | 0.51 | 46 | 21.47 | 1.17 | 88 | 24.00 | 0.60 |
| 5 | 13.97 | 1.07 | 47 | 21.47 | 0.85 | 89 | 24.07 | 0.83 |
| 6 | 14.28 | 0.95 | 48 | 21.51 | 1.09 | 90 | 24.47 | 0.92 |
| 7 | 14.73 | 0.25 | 49 | 21.53 | 0.39 | 91 | 24.55 | 1.12 |
| 8 | 14.80 | 0.76 | 50 | 21.53 | 0.87 | 92 | 24.72 | 0.20 |
| 9 | 14.93 | 0.88 | 51 | 21.56 | 0.51 | 93 | 24.76 | 0.73 |
| 10 | 14.96 | 0.66 | 52 | 21.70 | 0.32 | 94 | 24.80 | 0.75 |
| 11 | 15.41 | 0.97 | 53 | 21.90 | 0.96 | 95 | 24.87 | 0.37 |
| 12 | 15.76 | 0.81 | 54 | 21.98 | 0.74 | 96 | 24.99 | 0.50 |
| 13 | 15.78 | 0.83 | 55 | 22.10 | 0.43 | 97 | 25.04 | 0.90 |
| 14 | 16.65 | 0.71 | 56 | 22.12 | 0.83 | 98 | 25.04 | 0.49 |
| 15 | 17.83 | 0.74 | 57 | 22.12 | 0.70 | 99 | 25.05 | 0.86 |
| 16 | 18.15 | 0.61 | 58 | 22.13 | 0.92 | 100 | 25.11 | 0.79 |
| 17 | 18.25 | 1.00 | 59 | 22.13 | 1.09 | 101 | 25.28 | 1.10 |
| 18 | 18.29 | -0.59 | 60 | 22.17 | 0.24 | 102 | 25.33 | 0.90 |
| 19 | 18.59 | 0.82 | 61 | 22.19 | 0.60 | 103 | 25.38 | 0.94 |
| 20 | 18.66 | 0.53 | 62 | 22.19 | 0.78 | 104 | 25.40 | 0.80 |
| 21 | 19.51 | 0.70 | 63 | 22.28 | -0.16 | 105 | 25.57 | -0.12 |
| 22 | 19.54 | 0.97 | 64 | 22.28 | 1.05 | 106 | 25.61 | 0.96 |
| 23 | 19.73 | 0.85 | 65 | 22.46 | 0.93 | 107 | 25.63 | 0.83 |
| 24 | 20.01 | 0.18 | 66 | 22.46 | 0.68 | 108 | 25.71 | 0.21 |
| 25 | 20.10 | 0.70 | 67 | 22.55 | 0.58 | 109 | 25.79 | 0.58 |
| 26 | 20.12 | 0.13 | 68 | 22.66 | 0.79 | 110 | 25.88 | 0.71 |
| 27 | 20.13 | -0.14 | 69 | 22.66 | 0.79 | 111 | 25.90 | 1.07 |
| 28 | 20.24 | 0.73 | 70 | 22.70 | 0.69 | 112 | 25.92 | 0.94 |
| 29 | 20.25 | 0.09 | 71 | 22.77 | 0.49 | 113 | 26.02 | 0.48 |
| 30 | 20.59 | 0.58 | 72 | 22.79 | 1.04 | 114 | 26.06 | 0.76 |
| 31 | 20.65 | 0.80 | 73 | 22.85 | 0.85 | 115 | 26.14 | 0.63 |
| 32 | 20.66 | 0.67 | 74 | 22.87 | 1.05 | 116 | 26.34 | 0.93 |
| 33 | 20.77 | 0.92 | 75 | 23.00 | 0.99 | 117 | 26.49 | 0.82 |
| 34 | 20.78 | 0.98 | 76 | 23.06 | 0.96 | 118 | 26.66 | 0.91 |
| 35 | 20.81 | 0.59 | 77 | 23.19 | 0.74 | 119 | 26.68 | 0.55 |
| 36 | 20.81 | 0.59 | 78 | 23.24 | 0.97 | 120 | 26.76 | 0.65 |
| 37 | 20.81 | 0.40 | 79 | 23.55 | 0.71 | 121 | 27.03 | 0.66 |
| 38 | 20.87 | 1.04 | 80 | 23.61 | 0.96 | 122 | 27.05 | 0.86 |
| 39 | 21.00 | 1.09 | 81 | 23.62 | 0.65 | 123 | 27.37 | 0.83 |
| 40 | 21.03 | 0.81 | 82 | 23.64 | 0.79 | 124 | 27.45 | 0.06 |
| 41 | 21.06 | 0.42 | 83 | 23.66 | 1.02 | 125 | 27.49 | 0.73 |
| 42 | 21.12 | 0.93 | 84 | 23.67 | 0.80 | 126 | 27.75 | 0.82 |

^a^ Note that the numbering of the resonances does not correspond to the MurD residue numbers. The resonances are numbered according to the positions of the signals in the ^13^C dimension of the ^1^H/^13^C HSQC spectrum, starting from the most up-field position.
