# Supplementary material for: The Binding Mode of Second-Generation Sulfonamide Inhibitors of MurD: Clues for Rational Design of Potent MurD Inhibitors
Source: PLoS One. 2012 Dec 20;7(12):e52817. doi: 10.1371/journal.pone.0052817 (PMC3527612; doi:10.1371/journal.pone.0052817)
Supplement: Table S2 — Hydrogen bonds formed on the D-Glu mimetic ring during MD simulations. (DOC) [file pone.0052817.s010.doc]

**Table S2: Hydrogen bonds formed on the d-Glu mimetic ring during MD simulations.**

|  |  | **Average number of hydrogen bonds per trajectory** | | | | | | |
| --- | --- | --- | --- | --- | --- | --- | --- | --- |
| **Label** | **Group** | Lys348  (NH_3_) | Thr321  (OH) | Ser415  (OH) | Ser415  (NH) | Phe422  (NH) | Arg425  (guanidine) | total |
| **1a** | α-COOH | 1.4 | 1.0 | 0.0 | 0.0 | 0.0 | 0.0 | 2.4 |
|  | γ-COOH | 0.0 | 0.0 | 1.1 | 1.0 | 0.2 | 0.0 | 2.3 |
| **1b** | α-COOH | 1.6 | 0.9 | 0.0 | 0.0 | 0.0 | 0.0 | 2.5 |
|  | γ-COOH | 0.0 | 0.0 | 1.2 | 1.0 | 0.2 | 0.5 | 2.9 |
| **2a** | *o*-COOH | 0.0 | 0.0 | 0.0 | 0.0 | 0.0 | 0.0 | 0.0 |
|  | *p*-COOH | 0.0 | 0.0 | 0.9 | 0.3 | 0.7 | 0.0 | 1.9 |
| **2b** | *o*-COOH | 1.5 | 0.5 | 0.0 | 0.0 | 0.0 | 0.0 | 2.1 |
|  | *p*-COOH | 0.0 | 0.0 | 1.2 | 1.1 | 0.2 | 0.0 | 2.5 |
| **3a** | *o*-COOH | 1.4 | 0.9 | 0.0 | 0.0 | 0.0 | 0.0 | 2.3 |
|  | *p*-OH | 0.0 | 0.0 | 0.0 | 0.0 | 0.0 | 0.0 | 0.0 |
| **3b** | *o*-COOH | 1.3 | 0.0 | 0.0 | 0.0 | 0.0 | 0.0 | 1.3 |
|  | *p*-OH | 0.0 | 0.0 | 0.0 | 0.0 | 0.0 | 0.0 | 0.0 |
| **4a** | *o*-COOH | 0.5 | 0.0 | 0.0 | 0.0 | 0.0 | 0.0 | 0.5 |
|  | *m*-COOH | 0.0 | 0.0 | 1.3 | 1.0 | 0.2 | 0.0 | 2.4 |
| **4b** | *o*-COOH | 0.6 | 0.0 | 0.0 | 0.0 | 0.0 | 0.0 | 0.6 |
|  | *m*-COOH | 0.0 | 0.0 | 1.0 | 0.8 | 0.6 | 0.0 | 2.4 |
| **5a** | *m*-COOH | 1.1 | 0.6 | 0.0 | 0.0 | 0.0 | 0.0 | 1.8 |
|  | *m*-COOH | 0.0 | 0.0 | 1.5 | 1.0 | 0.3 | 0.0 | 2.8 |
| **5b** | *m*-COOH | 1.1 | 0.0 | 0.0 | 0.0 | 0.0 | 0.0 | 1.1 |
|  | *m*-COOH | 0.0 | 0.0 | 1.5 | 1.0 | 0.5 | 0.0 | 2.9 |
| **6a** | *o*-COOH | 1.2 | 0.0 | 0.0 | 0.0 | 0.0 | 0.0 | 1.3 |
|  | *p*-COOH | 0.0 | 0.0 | 1.7 | 1.0 | 0.6 | 0.0 | 3.2 |
| **6b** | *o*-COOH | 1.6 | 0.0 | 0.0 | 0.0 | 0.0 | 0.0 | 1.6 |
|  | *p*-COOH | 0.0 | 0.0 | 1.4 | 1.0 | 0.4 | 0.0 | 2.9 |
