# Supplementary material for: The Binding Mode of Second-Generation Sulfonamide Inhibitors of MurD: Clues for Rational Design of Potent MurD Inhibitors
Source: PLoS One. 2012 Dec 20;7(12):e52817. doi: 10.1371/journal.pone.0052817 (PMC3527612; doi:10.1371/journal.pone.0052817)
Supplement: Dataset S1 — Overlays of 1H/13C HSQC NMR spectra in absence and presence of the ligands. (DOC) [file pone.0052817.s011.doc]

**Dataset S1. Overlays of ^1^H/^13^C HSQC NMR spectra in absence and presence of the ligands.**


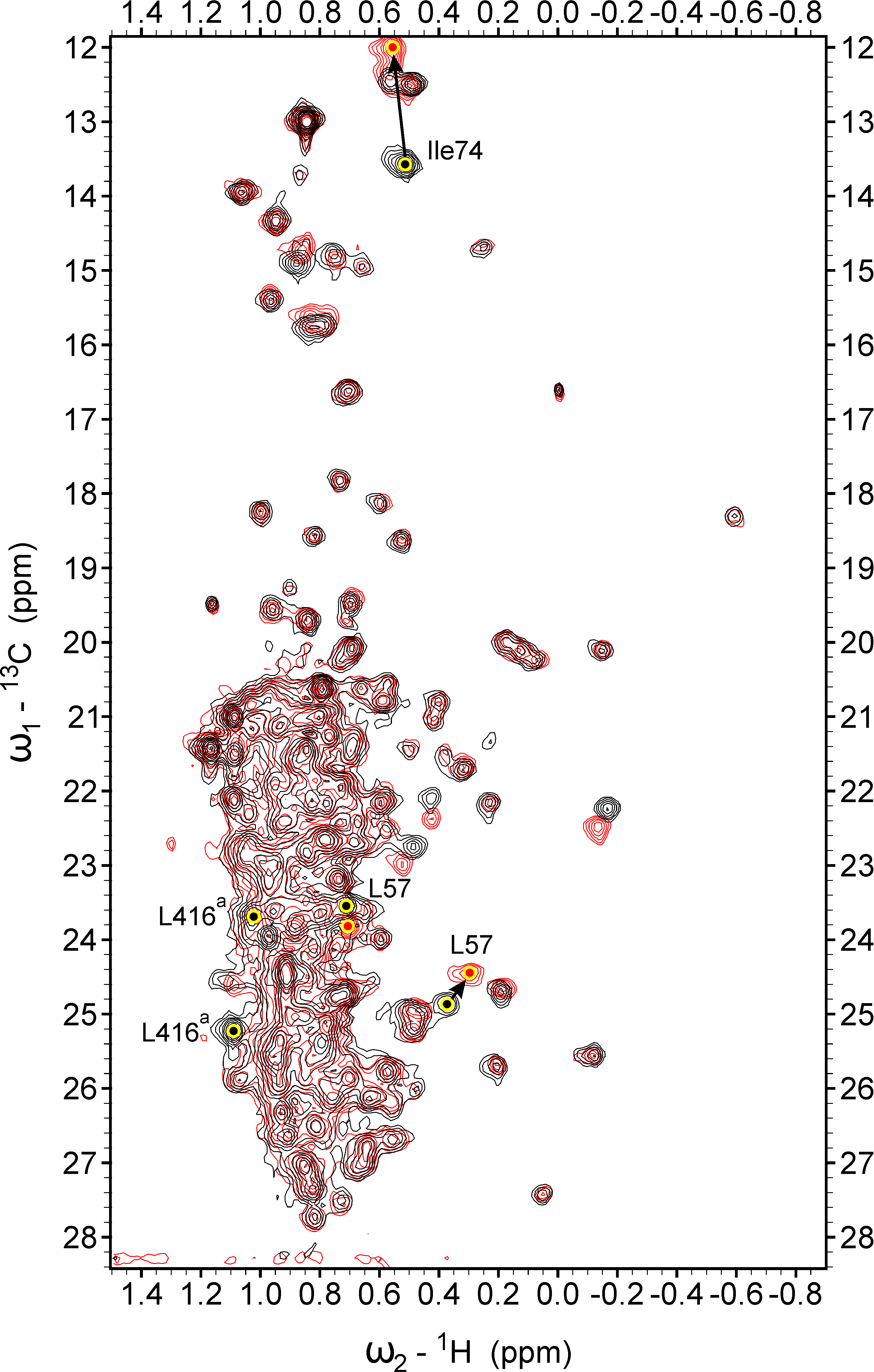


**Overlay of ^1^H/^13^C HSQC NMR spectra in absence (black) and presence (red) of compound 1b.** Ligand/protein ratio is 10:1. ^a^ Signals of Leu416 methyl groups disappear at 0.5:1 ligand/protein ratio. The new position of these signals cannot be identified because of the signal overlap. In such cases, the minimum possible CSPs are calculated.


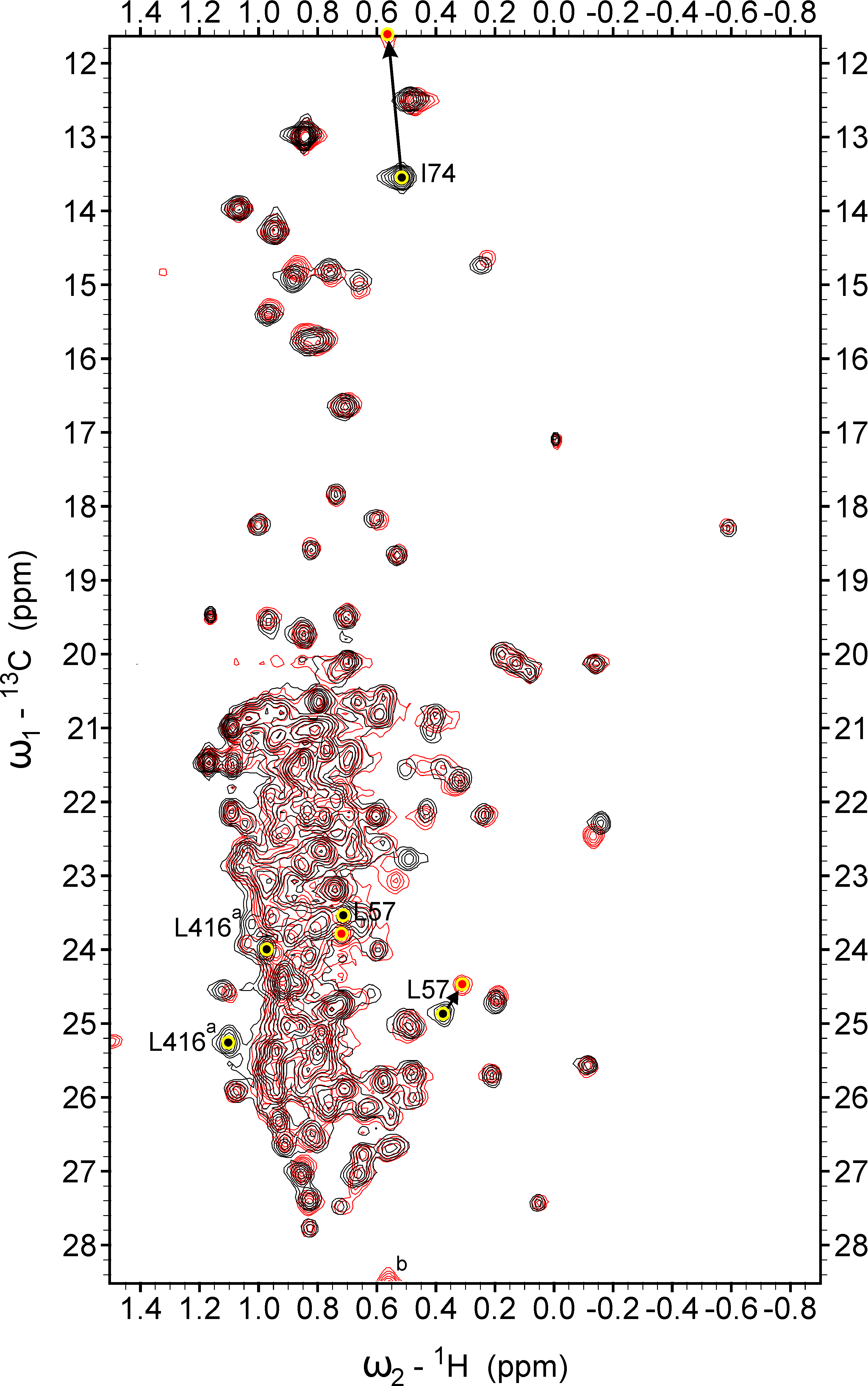


**Overlay of ^1^H/^13^C HSQC NMR spectra in absence (black) and presence (red) of compound 2b.** Ligand/protein ratio is 10:1. ^a^ The new position of these signals cannot be identified because of the signal overlap. In such cases, the minimum possible CSPs are calculated. ^b^ Folded signal of Ile74 methyl group.


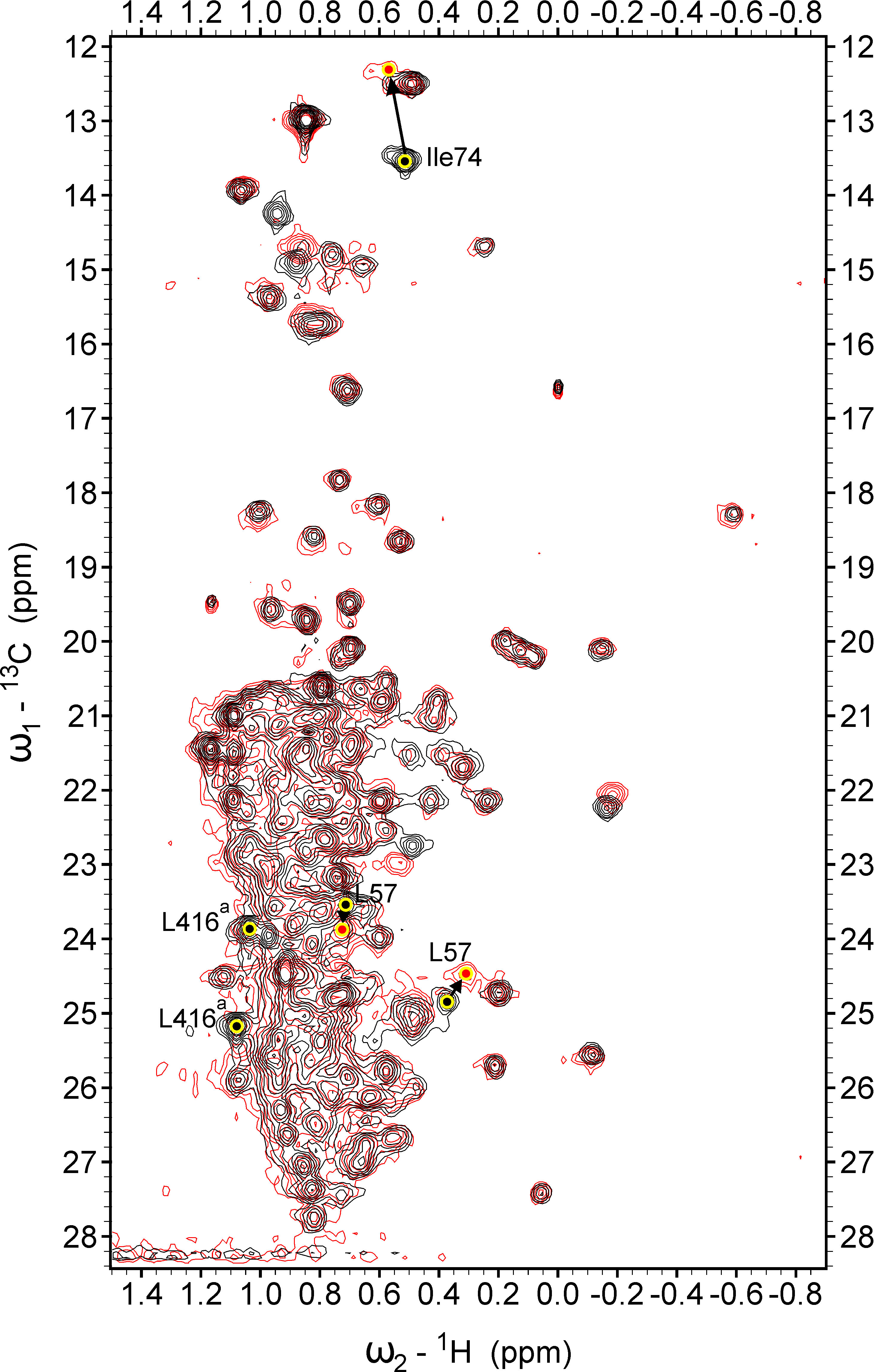


**Overlay of ^1^H/^13^C HSQC NMR spectra in absence (black) and presence (red) of compound 3b.** Ligand/protein ratio is 10:1. ^a^ Signals of Leu416 methyl groups disappear at 0.5:1 ligand/protein ratio. The new position of these signals cannot be identified because of the signal overlap. In such cases, the minimum possible CSPs are calculated.


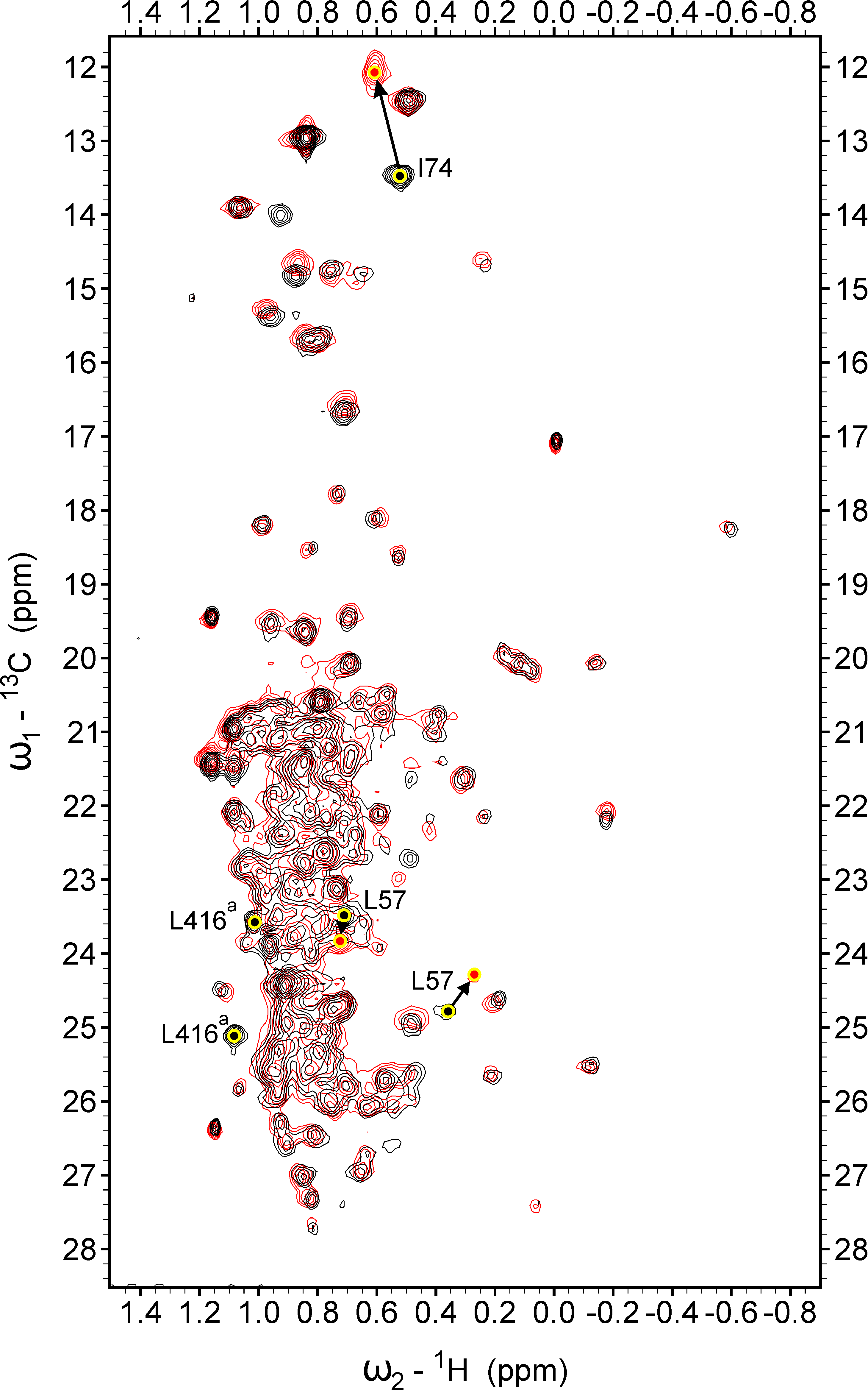


**Overlay of ^1^H/^13^C HSQC NMR spectra in absence (black) and presence (red) of compound 4b.** Ligand/protein ratio is 10:1. ^a^ Signals of Leu416 methyl groups disappear at 0.5:1 ligand/protein ratio. The new position of these signals cannot be identified because of the signal overlap. In such cases, the minimum possible CSPs are calculated.


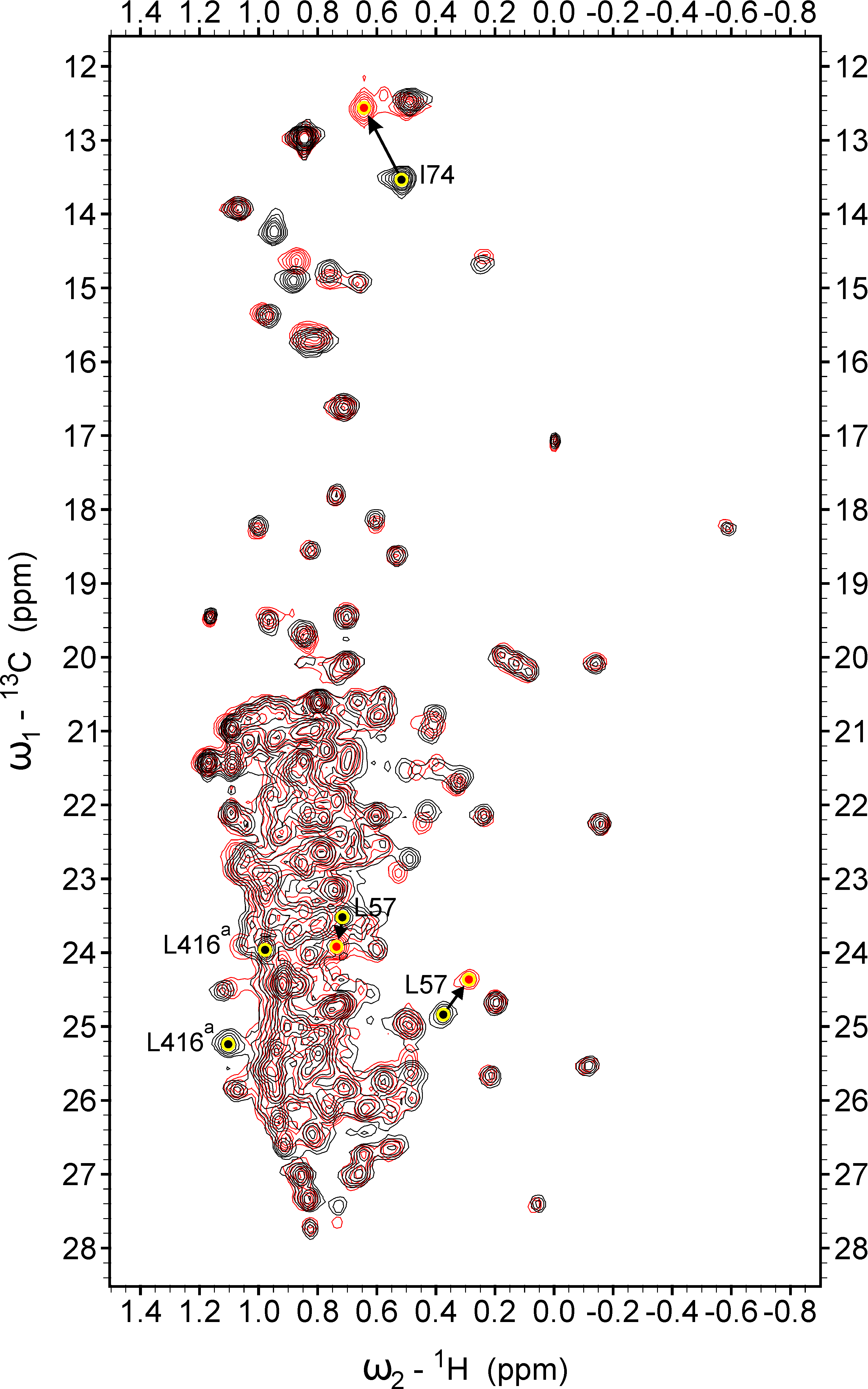


**Overlay of ^1^H/^13^C HSQC NMR spectra in absence (black) and presence (red) of compound 5b.** Ligand/protein ratio is 10:1. ^a^ Signals of Leu416 methyl groups disappear at 0.5:1 ligand/protein ratio. The new position of these signals cannot be identified because of the signal overlap. In such cases, the minimum possible CSPs are calculated.


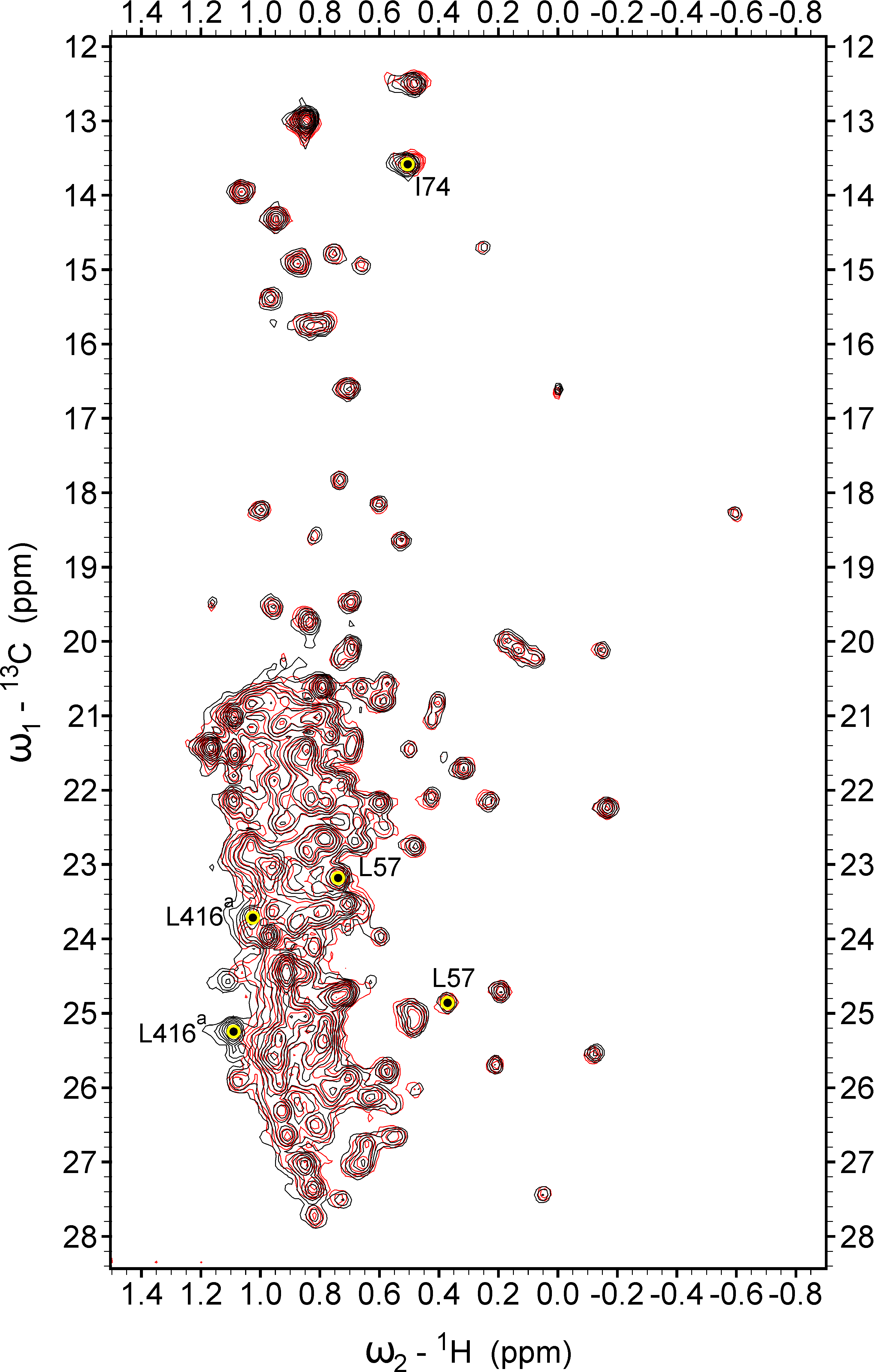


**Overlay of ^1^H/^13^C HSQC NMR spectra in absence (black) and presence (red) of compound 1.**  Ligand/protein ratio is 10:1. ^a^ Signals of Leu416 methyl groups disappear at 2:1 ligand/protein ratio. The new position of these signals cannot be identified because of the signal overlap. In such cases, the minimum possible CSPs are calculated.


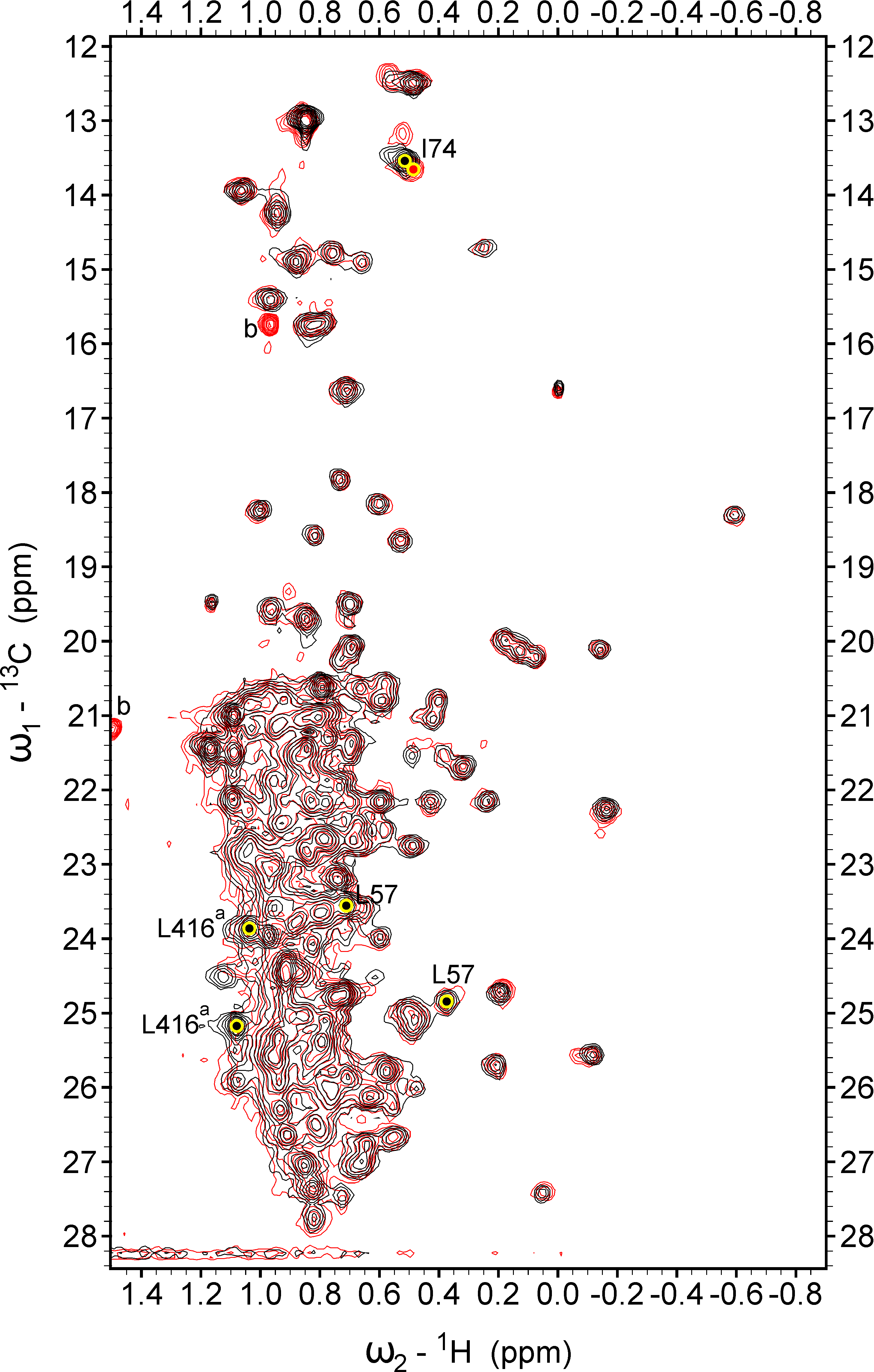


**Overlay of ^1^H/^13^C HSQC NMR spectra in absence (black) and presence (red) of compound 1a.**  ^a^ Signals of Leu416 methyl groups disappear at 0.5:1 ligand/protein ratio. The new position of these signals cannot be identified because of the signal overlap. In such cases, the minimum possible CSPs are calculated. ^b^ Two signals from the ligand aliphatic group.


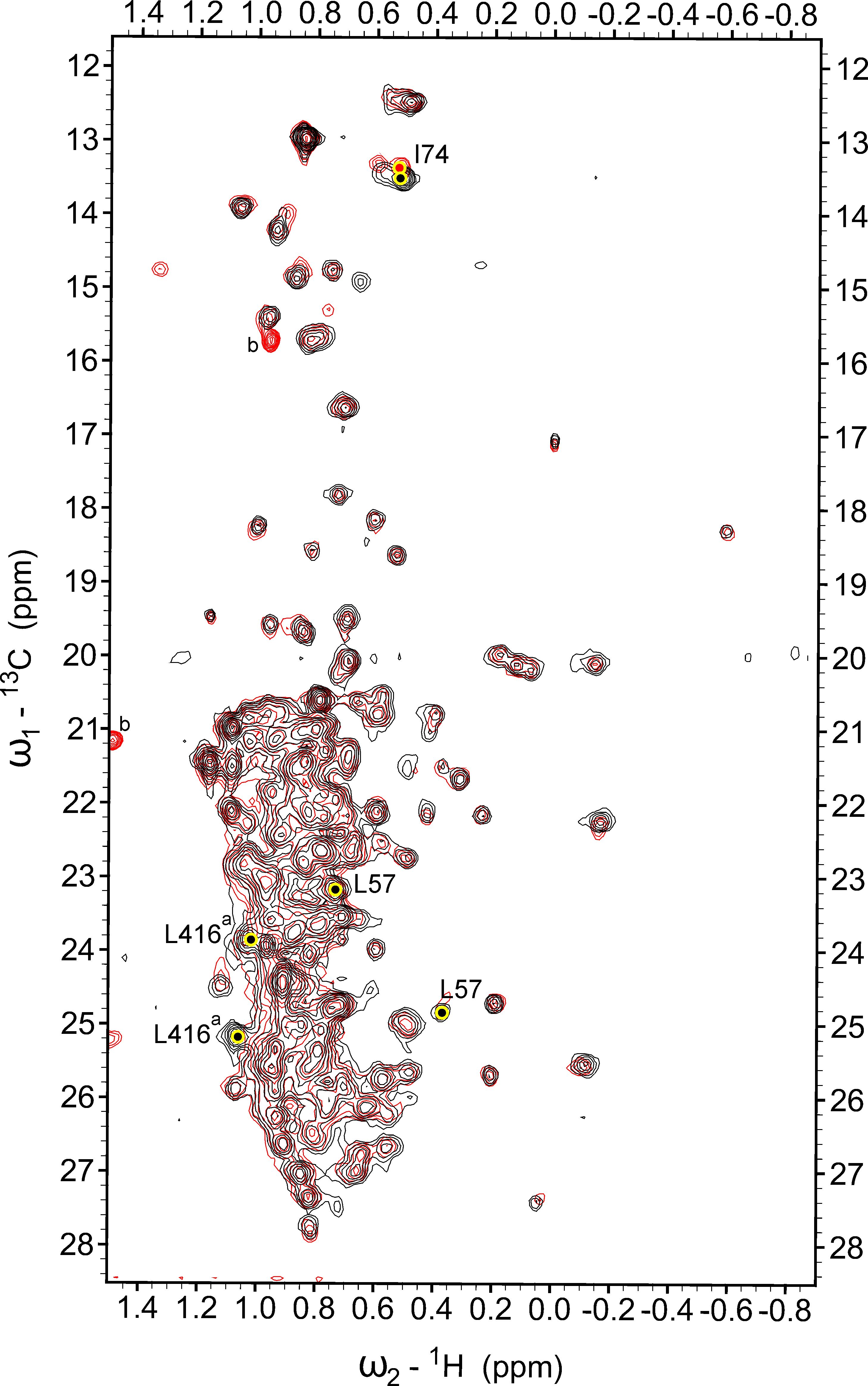


**Overlay of ^1^H/^13^C HSQC NMR spectra in absence (black) and presence (red) of compound 2a.**  ^a^ Signals of Leu416 methyl groups disappear at 0.5:1 ligand/protein ratio. The new position of these signals cannot be identified because of the signal overlap. In such cases, the minimum possible CSPs are calculated. ^b^ Two signals from the ligand aliphatic group.


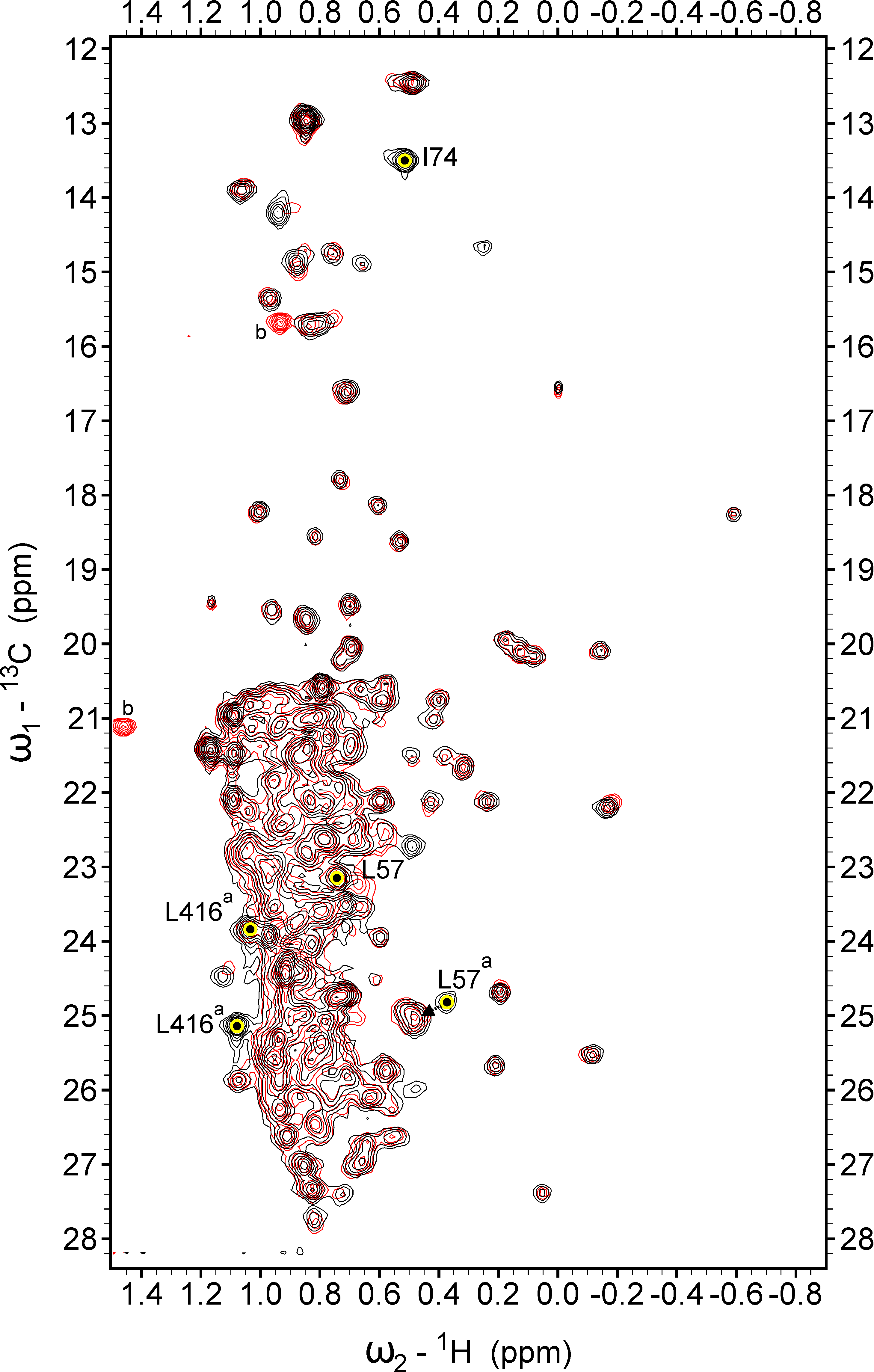


**Overlay of ^1^H/^13^C HSQC NMR spectra in absence (black) and presence (red) of compound 5a.** ^a^ Signals of Leu416 methyl groups disappear at 0.5:1 ligand/protein ratio. The new position of these signals cannot be identified because of the signal overlap. In such cases, the minimum possible CSPs are calculated. ^b^ Two signals from the ligand aliphatic group.


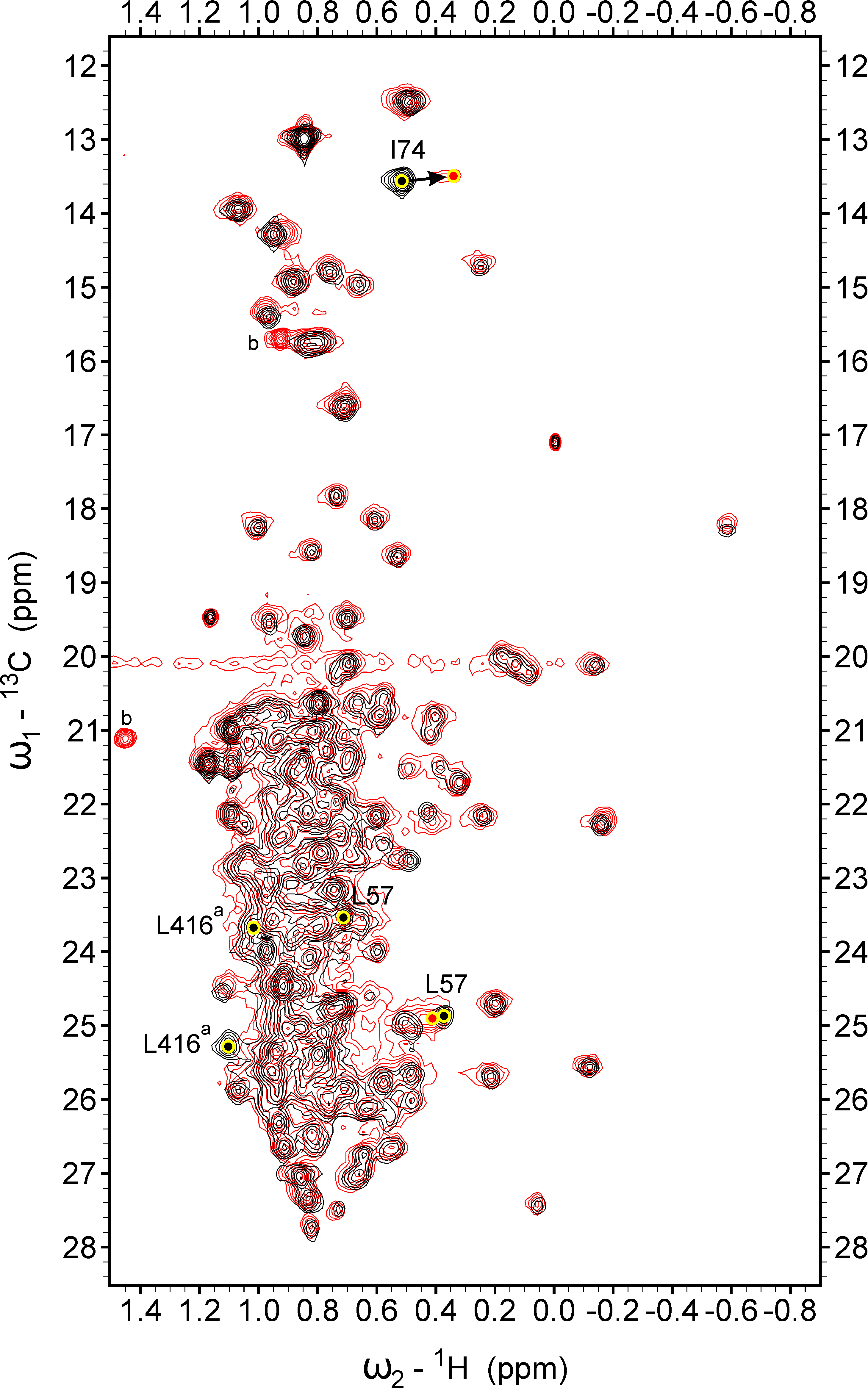


**Overlay of ^1^H/^13^C HSQC NMR spectra in absence (black) and presence (red) of compound 6a.** ^a^ Signals of Leu416 methyl groups disappear at 0.5:1 ligand/protein ratio. The new position of these signals cannot be identified because of the signal overlap. In such cases, the minimum possible CSPs are calculated. ^b^ Two signals from the ligand aliphatic group.


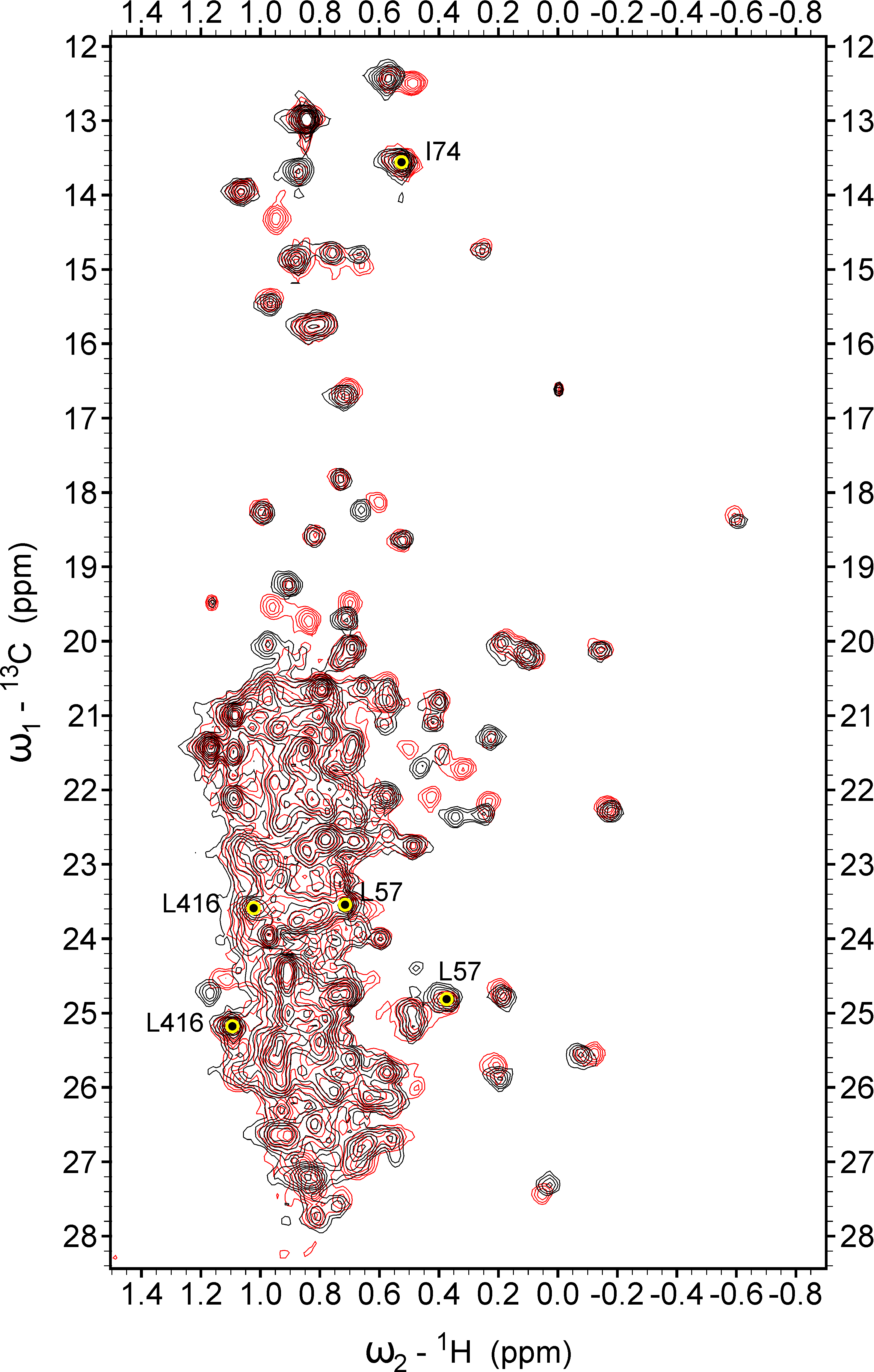


**Overlay of ^1^H/^13^C HSQC NMR spectra in absence (black) and presence (red) of AMPPCP.** Ligand/protein ratio is 20:1.
