# Supplementary material for: The Binding Mode of Second-Generation Sulfonamide Inhibitors of MurD: Clues for Rational Design of Potent MurD Inhibitors
Source: PLoS One. 2012 Dec 20;7(12):e52817. doi: 10.1371/journal.pone.0052817 (PMC3527612; doi:10.1371/journal.pone.0052817)
Supplement: Dataset S2 — Chemical shifts predicted in program SHIFTS 4.1.1. (DOC) [file pone.0052817.s012.doc]

**Dataset S2. Chemical shifts predicted in program SHIFTS 4.1.1.** Structures 2VTD [8], 2JFF [7], and 2XPC [11] are used for shifts prediction. Only the residues that are in the 12 Å range from the ligand are considered (based on distances from crystal structure 2VTD ).

|  | **Predicted** | | | | | |  | |
| --- | --- | --- | --- | --- | --- | --- | --- | --- |
|  | **PDB entry 2VTD** | | **PDB entry 2JFF** | | **PDB entry 2XPC** | | **Standard deviation** | |
| **residue** | **δ1 (ppm)** |  | **δ1 (ppm)** |  | **δ1 (ppm)** |  | **δ1 (ppm)** |  |
| Ile10 | 0.78 |  | 0.77 |  | 0.77 |  | 0.01 |  |
| Ile11 | 0.67 |  | 0.68 |  | 0.69 |  | 0.01 |  |
| Ile68 | 0.76 |  | 0.77 |  | 0.78 |  | 0.01 |  |
| Ile74 | 0.50 |  | 0.51 |  | 0.61 |  | 0.06 |  |
| Ile95 | 0.67 |  | 0.68 |  | 0.70 |  | 0.02 |  |
| Ile139 | 0.83 |  | 0.78 |  | 0.78 |  | 0.03 |  |

|  | **Predicted** | | | | | |  | |
| --- | --- | --- | --- | --- | --- | --- | --- | --- |
|  | **PDB entry 2VTD** | | **PDB entry 2JFF** | | **PDB entry 2XPC** | | **Standard deviation** | |
| **residue** | **γ1 (ppm)** | **γ2 (ppm)** | **γ1 (ppm)** | **γ2 (ppm)** | **γ1 (ppm)** | **γ2 (ppm)** | **γ1 (ppm)** | **γ2 (ppm)** |
| Val9 | 0.67 | 0.60 | 0.68 | 0.62 | 0.66 | 0.65 | 0.01 | 0.03 |
| Val33 | 0.77 | 0.75 | 0.74 | 0.78 | 0.72 | 0.77 | 0.03 | 0.02 |
| Val69 | 0.72 | 0.73 | 0.68 | 0.75 | 0.70 | 0.78 | 0.02 | 0.03 |
| Val323 | 0.90 | 1.14 | 0.90 | 1.13 | 0.90 | 1.14 | 0.00 | 0.01 |

|  | **Predicted** | | | | | |  | |
| --- | --- | --- | --- | --- | --- | --- | --- | --- |
|  | **PDB entry 2VTD** | | **PDB entry 2JFF** | | **PDB entry 2XPC** | | **Standard deviation** | |
| **residue** | **δ1 (ppm)** | **δ2 (ppm)** | **δ1 (ppm)** | **δ2 (ppm)** | **δ1 (ppm)** | **δ2 (ppm)** | **δ1 (ppm)** | **δ2 (ppm)** |
| Leu13 | 0.74 | 0.46 | 0.76 | 0.69 | 0.74 | 0.62 | 0.01 | 0.12 |
| Leu15 | 0.95 | 0.88 | 0.95 | 0.89 | 0.94 | 0.89 | 0.01 | 0.01 |
| Leu43 | 0.91 | 0.87 | 0.92 | 0.86 | 0.91 | 0.86 | 0.01 | 0.01 |
| Leu57 | 0.27 | 0.54 | 0.26 | 0.58 | 0.27 | 0.62 | 0.01 | 0.04 |
| Leu62 | 0.60 | 0.74 | 0.6 | 0.74 | 0.58 | 0.75 | 0.01 | 0.01 |
| Leu76 | 0.86 | 0.83 | 0.87 | 0.83 | 0.88 | 0.84 | 0.01 | 0.01 |
| Leu81 | 0.56 | 0.73 | 0.82 | 0.69 | 0.75 | 0.79 | 0.13 | 0.05 |
| Leu158 | 0.77 | 0.66 | 0.78 | 0.69 | 0.78 | 0.67 | 0.01 | 0.02 |
| Leu163 | 0.60 | 0.68 | 0.57 | 0.69 | 0.58 | 0.69 | 0.02 | 0.01 |
| Leu409 | 0.96 | 0.56 | 0.94 | 0.55 | 0.94 | 0.54 | 0.01 | 0.01 |
| Leu416 | 1.09 | 0.90 | 1.08 | 0.89 | 1.06 | 0.87 | 0.02 | 0.02 |

|  | **Experimental** | |
| --- | --- | --- |
| **Proposed residue** | **δ1** | **δ2** |
| Ile74 | 0.51 |  |
| Leu57 | 0.37 | 0.71 |
| Leu416 | 1.10 | 1.02 |


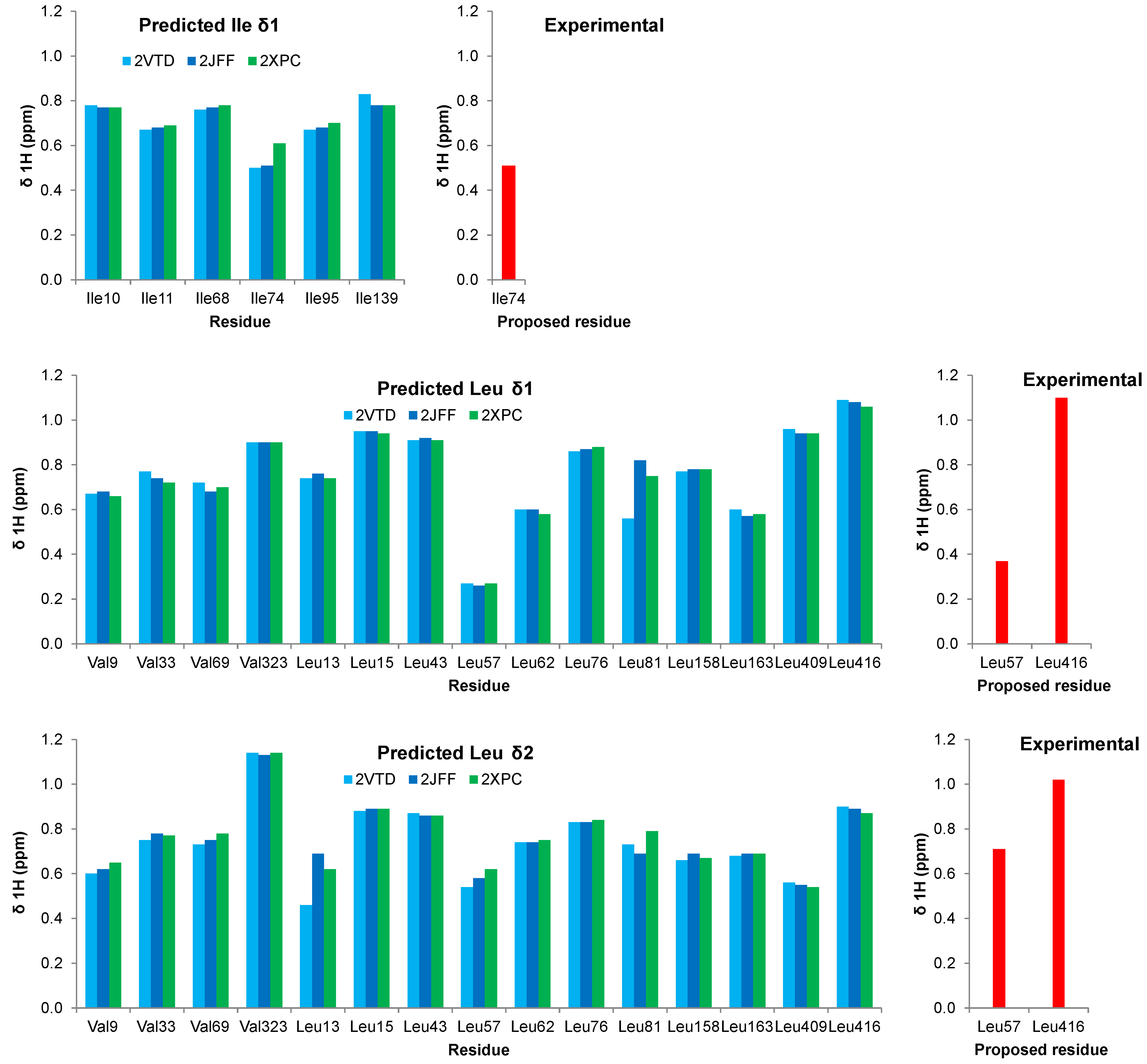


Charts representing predicted shifts for crystal structures 2VTD [8], 2JFF [7], and 2XPC [11] compared to experimental chemical shifts of the signals proposed as Ile74, Leu57, and Leu416.
