# Supplementary material for: The Binding Mode of Second-Generation Sulfonamide Inhibitors of MurD: Clues for Rational Design of Potent MurD Inhibitors
Source: PLoS One. 2012 Dec 20;7(12):e52817. doi: 10.1371/journal.pone.0052817 (PMC3527612; doi:10.1371/journal.pone.0052817)
Supplement: Dataset S3 — MD analysis data. (DOC) [file pone.0052817.s013.doc]

**Dataset S3: MD analysis data**


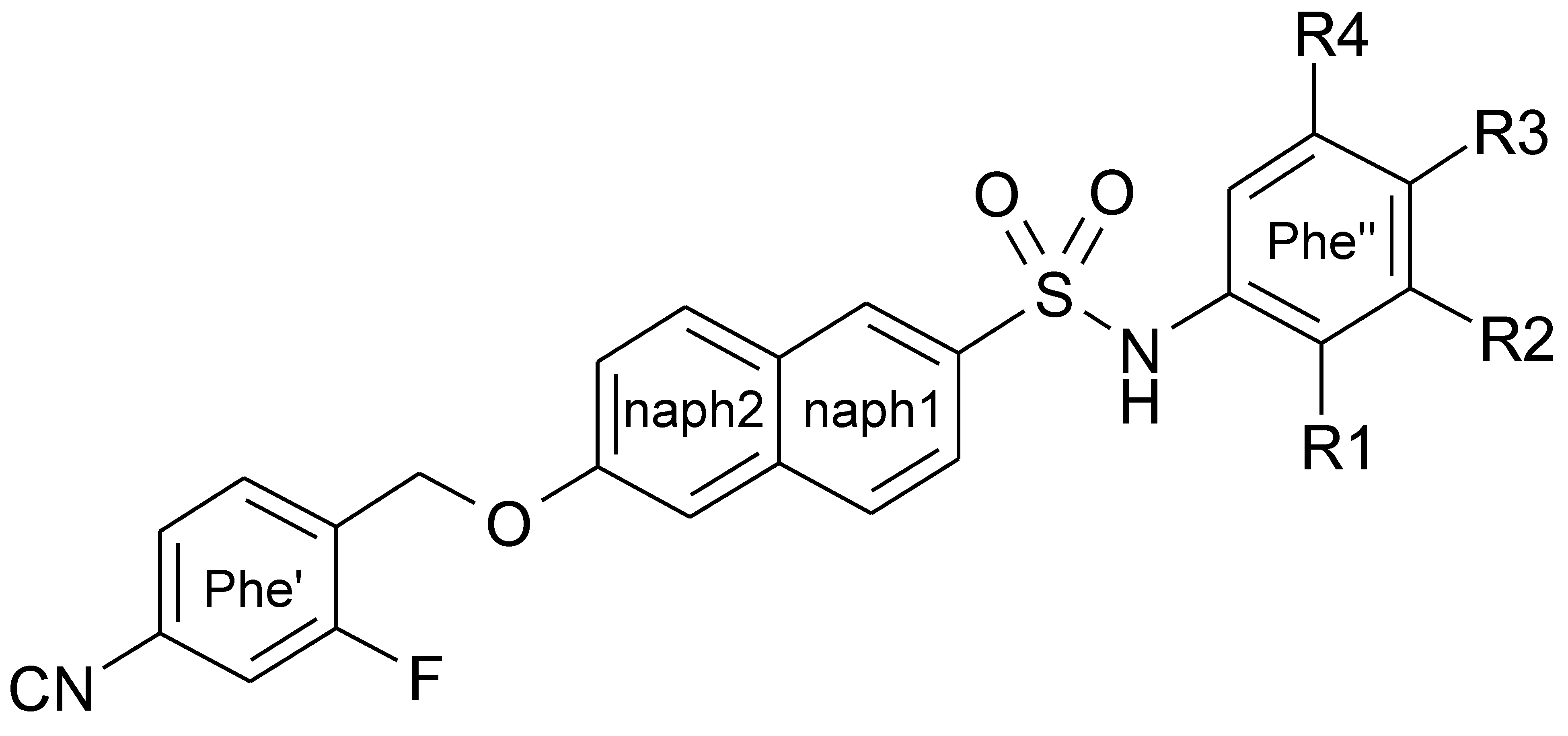


Ring labels used in MD analysis.

Compound **1a**

**
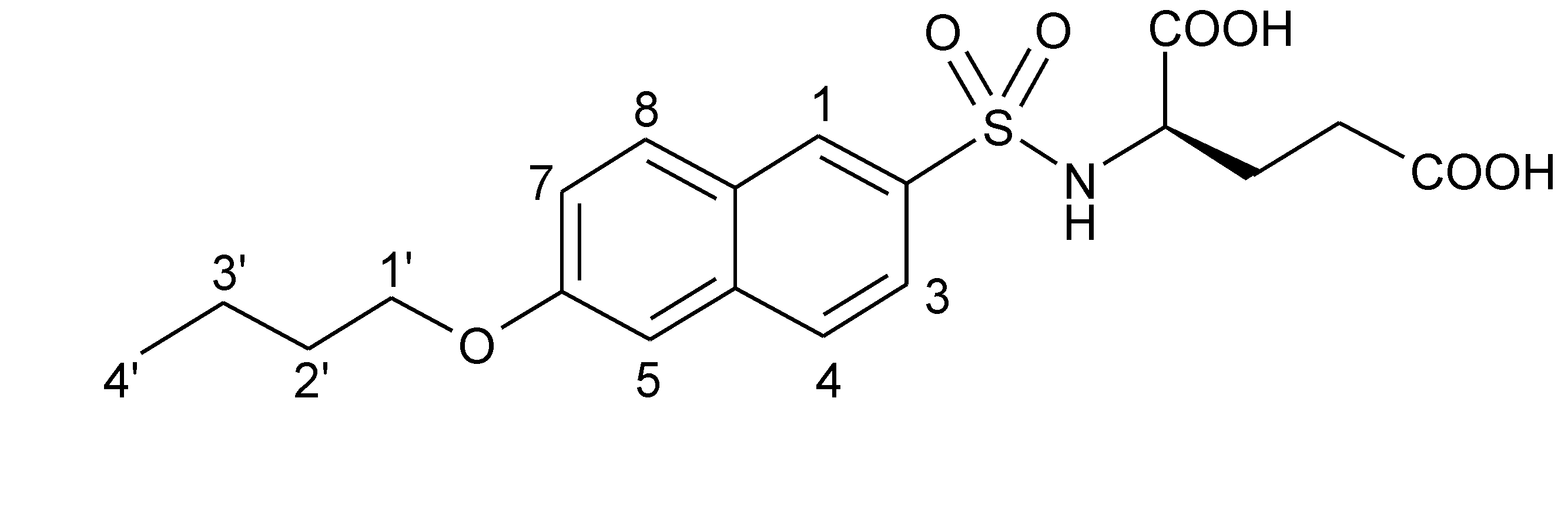
**

|  |  |
| --- | --- |
|  |  |
|  |  |
|  |  |

Selected ligand distances and dihedral angles during the MD simulation of the **1a**–MurD complex.

|  |  |
| --- | --- |
|  |  |

Selected ligand–protein distances indicating the interactions during the MD simulation of the **1a**–MurD complex.

|  |  |
| --- | --- |
|  |  |
|  |  |

Selected ligand–protein distances indicating the hydrogen bonds during the MD simulation of the **1a**–MurD complex

Compound **1b**


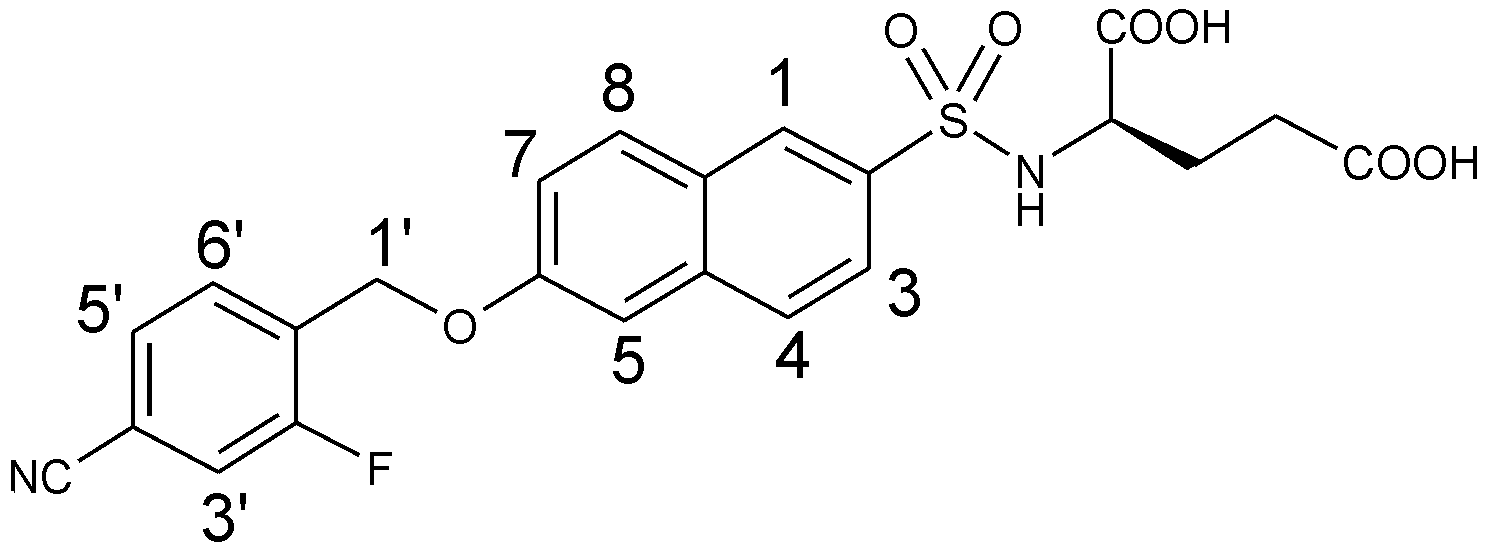


|  |  |
| --- | --- |
|  |  |
|  |  |
|  |  |

Selected ligand distances and dihedral angles during the MD simulation of the **1b**–MurD complex.

|  |  |
| --- | --- |
|  |  |
|  |  |

Selected ligand–protein distances indicating the interactions during the MD simulation of the **1b**–MurD complex.

|  |  |
| --- | --- |
|  |  |
|  |  |
|  |  |

Selected ligand–protein distances indicating the hydrogen bonds during the MD simulation of the **1b**–MurD complex.

Compound **2a**

**
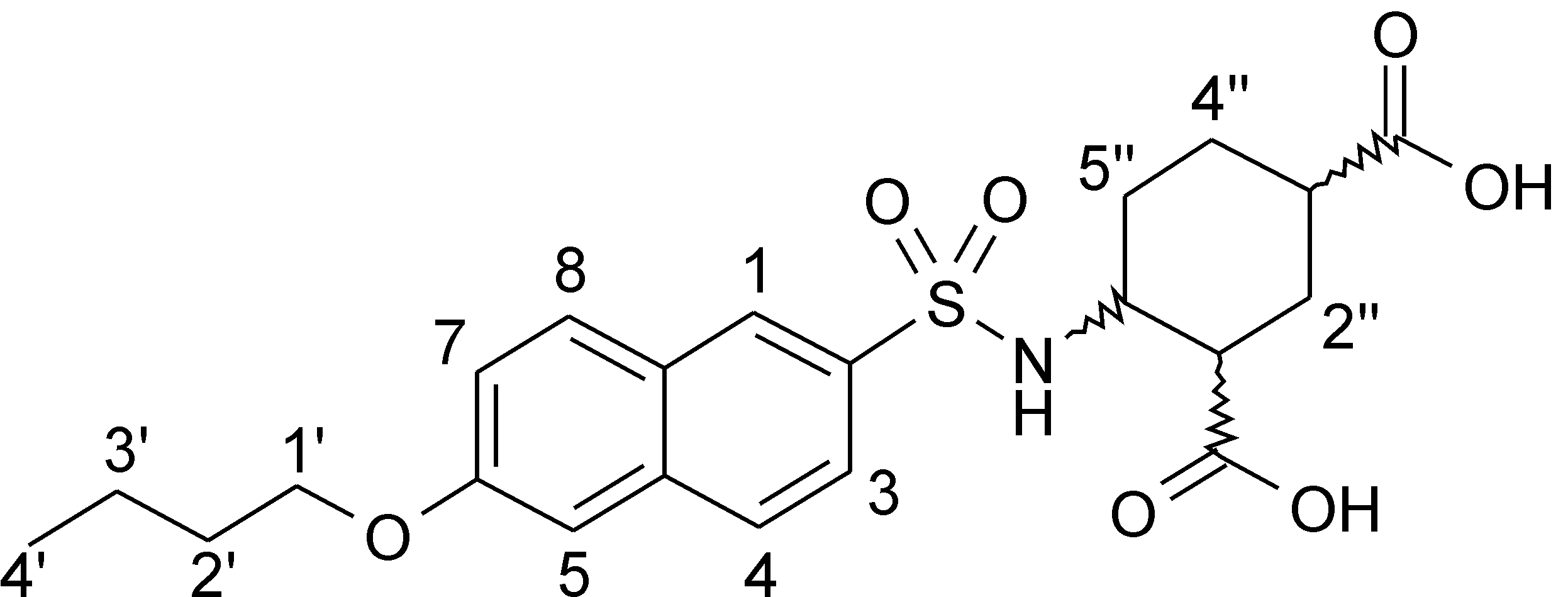
**

|  |  |
| --- | --- |
|  |  |
|  |  |
|  |  |

Selected ligand distances and dihedral angles during the MD simulation of the **2a**–MurD complex.

|  |  |
| --- | --- |
|  |  |
|  |  |

Selected ligand–protein distances indicating the interactions during the MD simulation of the **2a**–MurD complex.

|  |  |
| --- | --- |
|  |  |
|  |  |

Selected ligand–protein distances indicating the hydrogen bonds during the MD simulation of the **2a**–MurD complex.

Compound **2b**


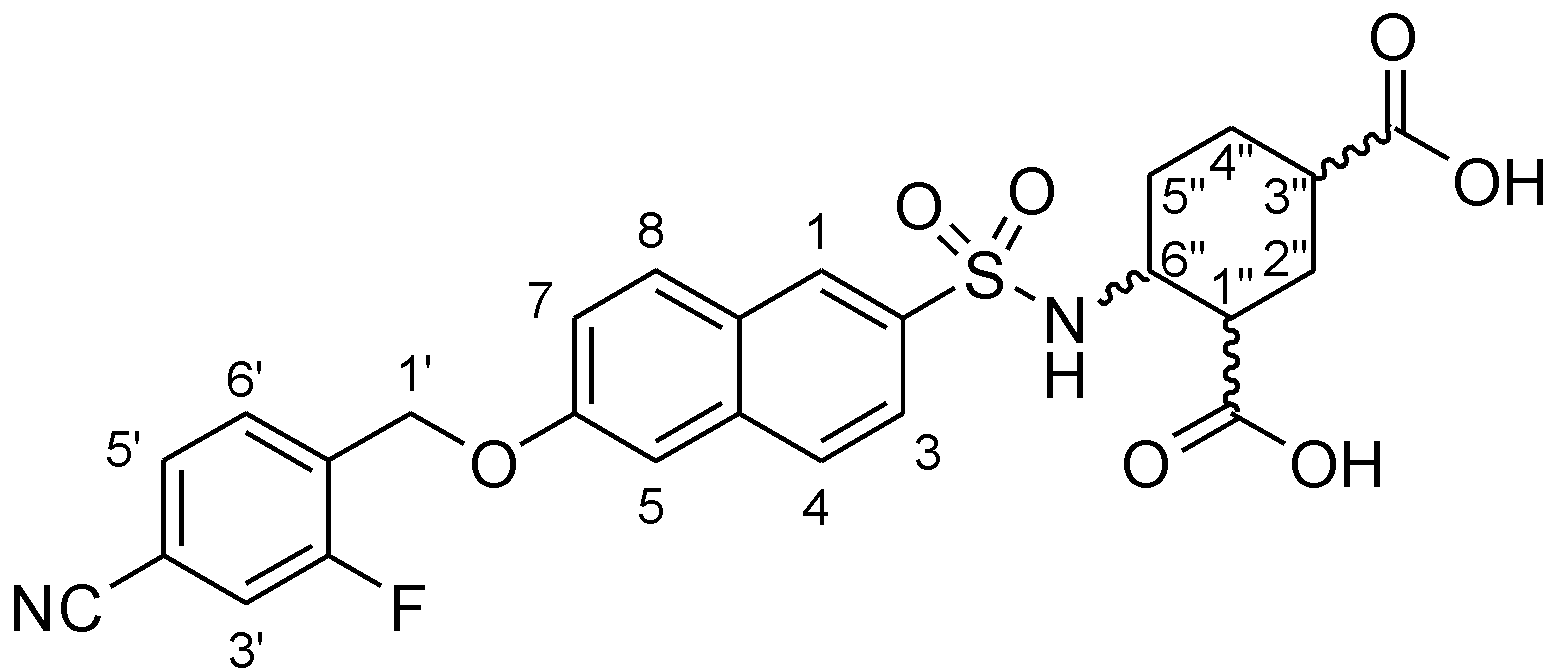


|  |  |
| --- | --- |
|  |  |
|  |  |
|  |  |

Selected ligand distances and dihedral angles during the MD simulation of the **2b**–MurD complex.

|  |  |
| --- | --- |
|  |  |
|  |  |

Selected ligand–protein distances indicating the interactions during the MD simulation of the **2b**–MurD complex.

|  |  |
| --- | --- |
|  |  |
|  |  |
|  |  |

Selected ligand–protein distances indicating the hydrogen bonds during the MD simulation of the **2b**–MurD complex.

Compound **3a**

**
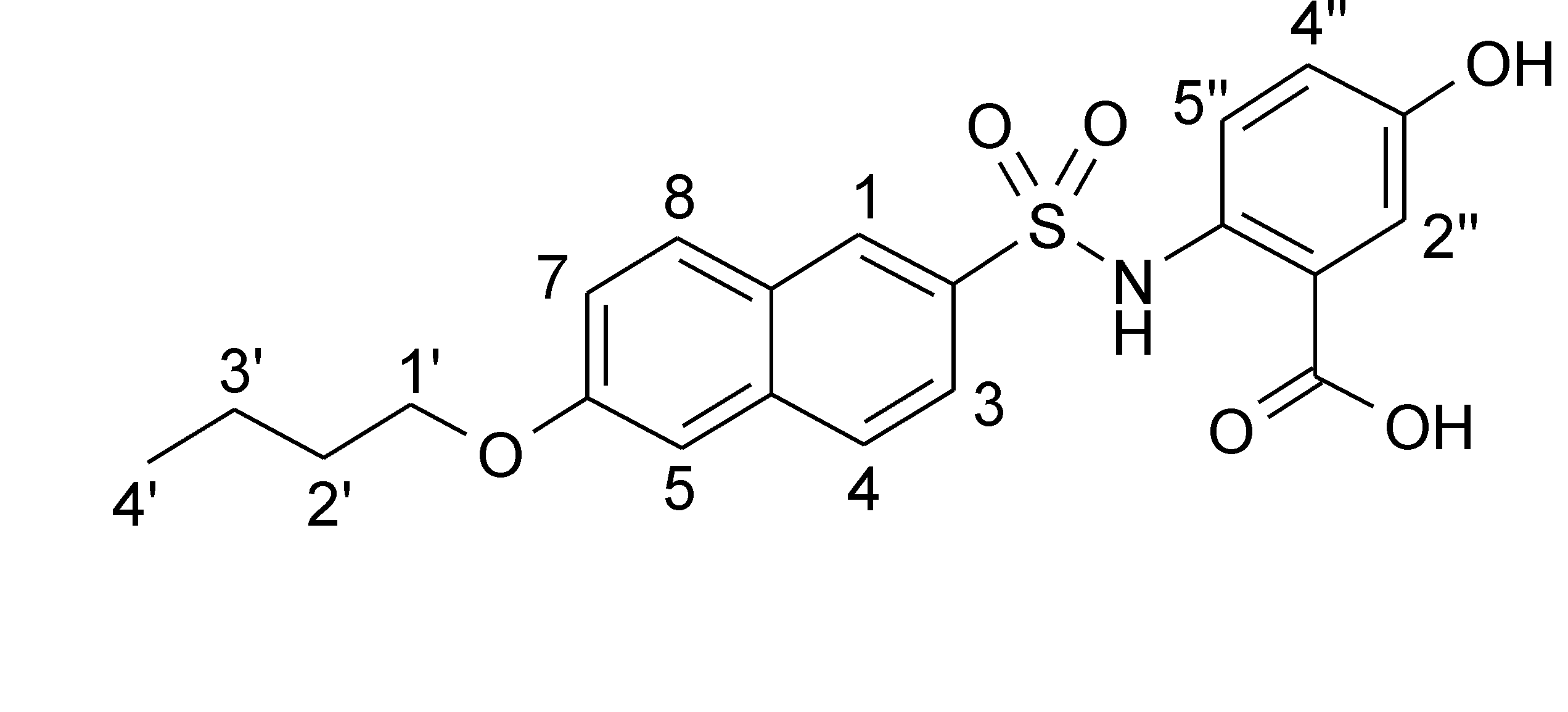
**

|  |  |
| --- | --- |
|  |  |
|  |  |
|  |  |

Selected ligand distances and dihedral angles during the MD simulation of the **3a**–MurD complex.

|  |  |
| --- | --- |
|  |  |
|  |  |

Selected ligand–protein distances indicating the interactions during the MD simulation of the **3a**–MurD complex.

|  |  |
| --- | --- |
|  |  |

Selected ligand–protein distances indicating the hydrogen bonds during the MD simulation of the **3a**–MurD complex.

Compound **3b**


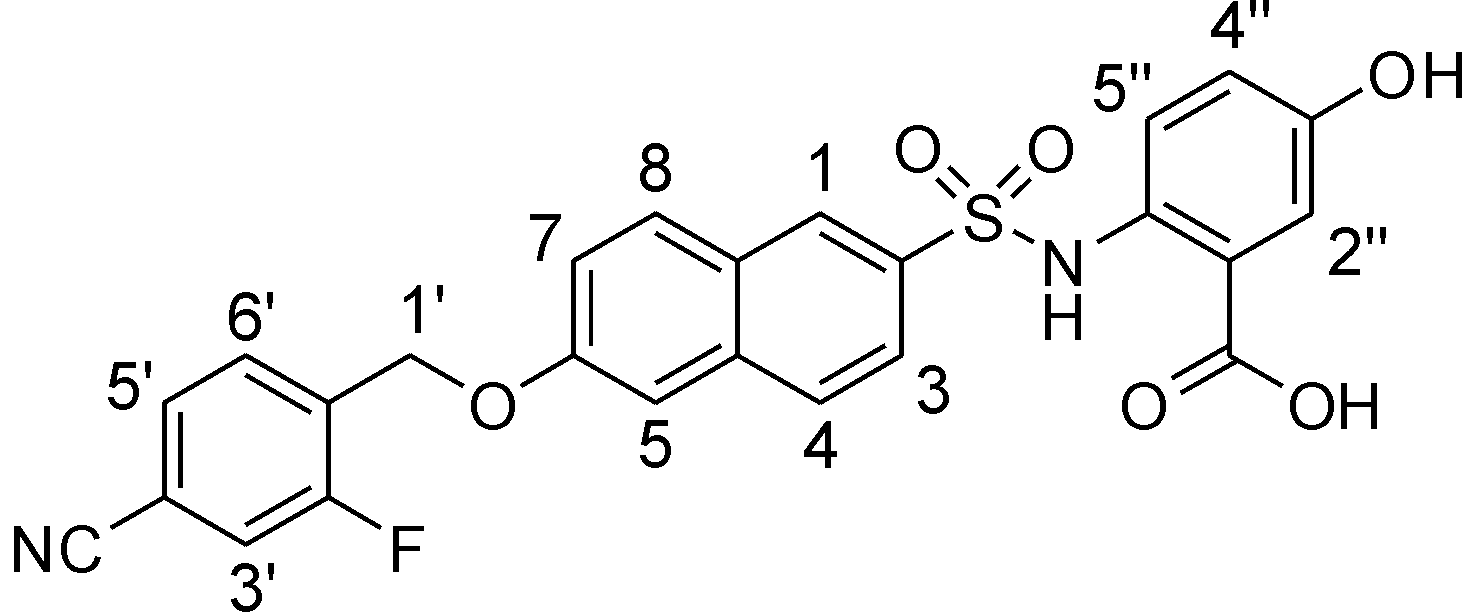


|  |  |
| --- | --- |
|  |  |
|  |  |
|  |  |

Selected ligand distances and dihedral angles during the MD simulation of the **3b**–MurD complex.

|  |  |
| --- | --- |
|  |  |
|  |  |
|  |  |

Selected ligand–protein distances indicating the interactions during the MD simulation of the **3b**–MurD complex.

|  |  |
| --- | --- |
|  |  |
|  |  |

Selected ligand–protein distances indicating the hydrogen bonds during the MD simulation of the  **3b**–MurD complex.

Compound **4a**

**
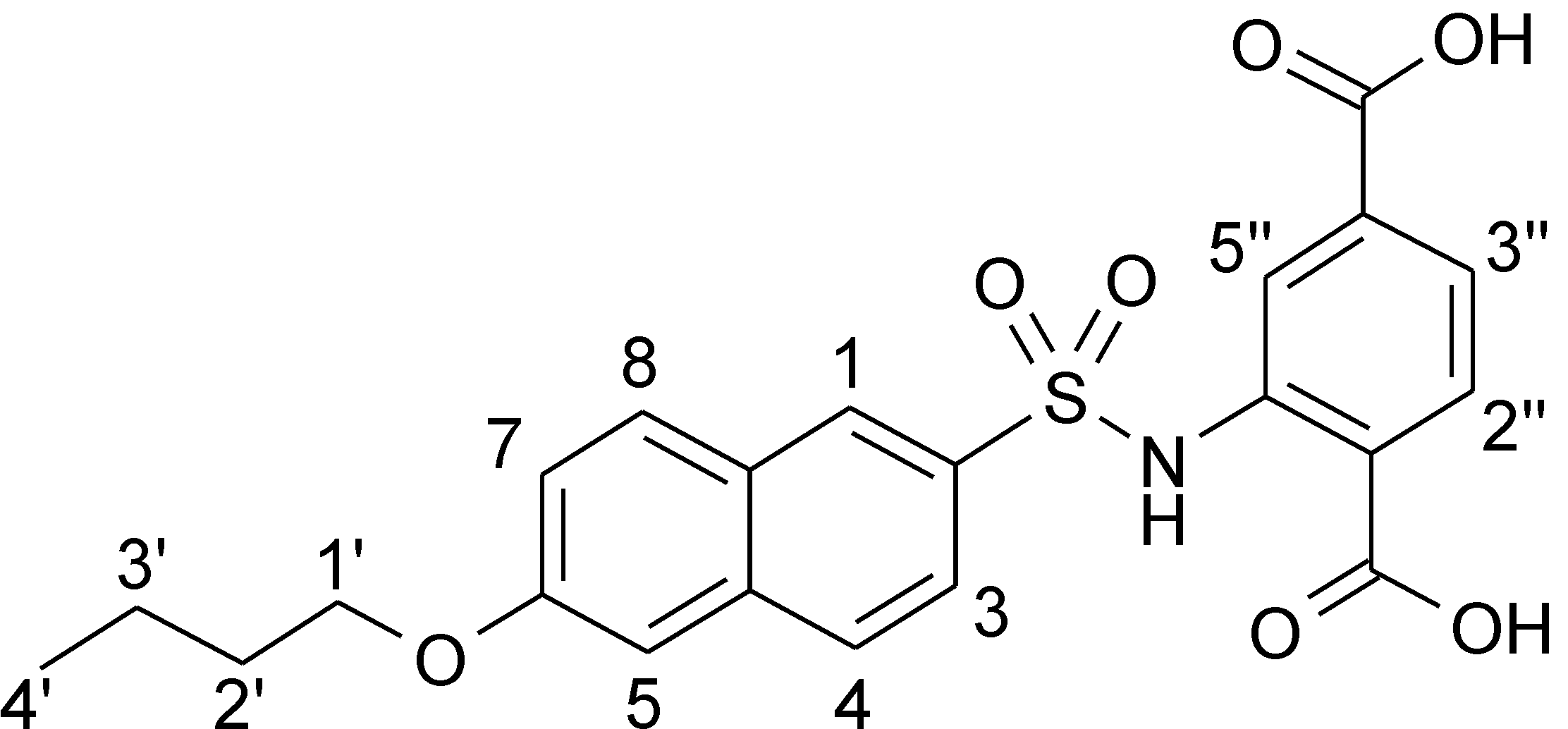
**

|  |  |
| --- | --- |
|  |  |
|  |  |
|  |  |

Selected ligand distances and dihedral angles during the MD simulation of the **4a**–MurD complex.

|  |  |
| --- | --- |
|  |  |
|  |  |

Selected ligand–protein distances indicating the interactions during the MD simulation of the **4a**–MurD complex.

|  |  |
| --- | --- |
|  |  |
|  |  |

Selected ligand–protein distances indicating the hydrogen bonds during the MD simulation of the **4a**–MurD complex.

Compound **4b**

**
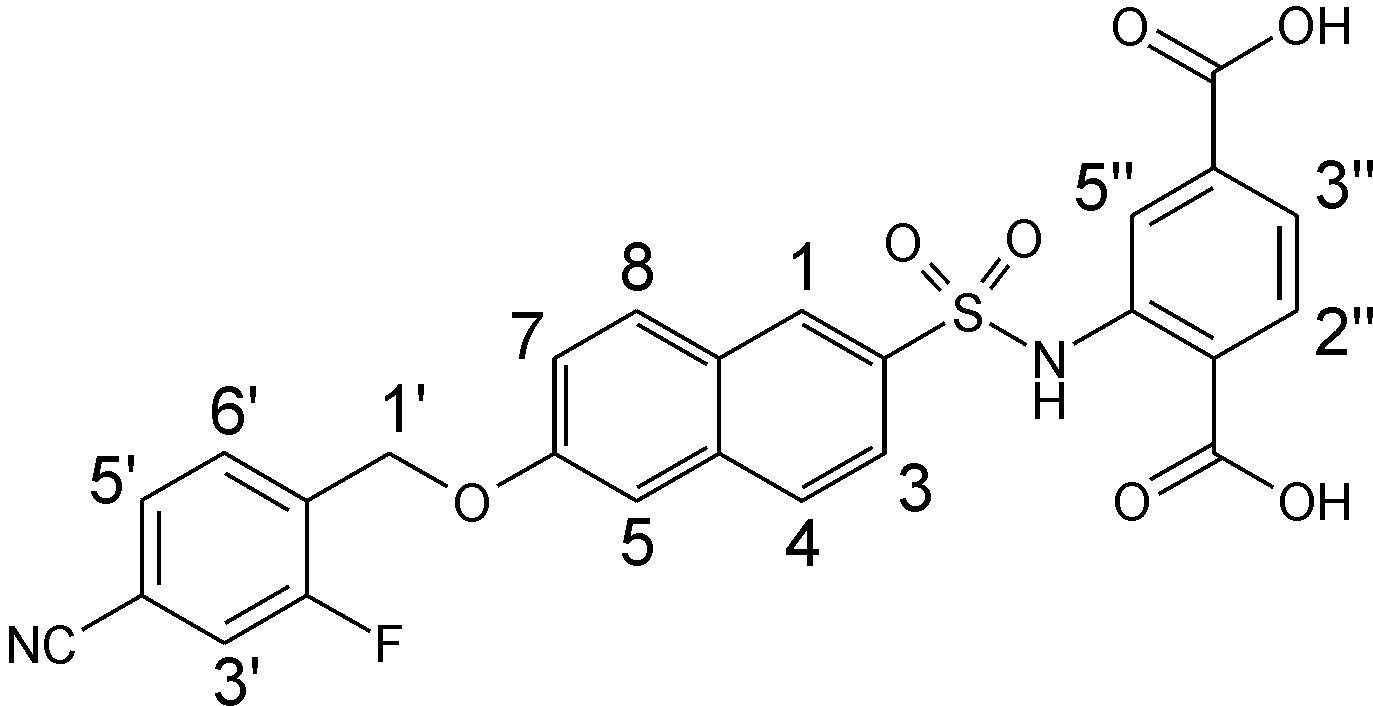
**

|  |  |
| --- | --- |
|  |  |
|  |  |
|  |  |

Selected ligand distances and dihedral angles during the MD simulation of the **4b**–MurD complex.

|  |  |
| --- | --- |
|  |  |
|  |  |
|  |  |

Selected ligand–protein distances indicating the interactions during the MD simulation of the **4b**–MurD complex.

|  |  |
| --- | --- |
|  |  |
|  |  |
|  |  |

Selected ligand–protein distances indicating the hydrogen bonds during the MD simulation of the **4b**–MurD complex.

Compound **5a**

**
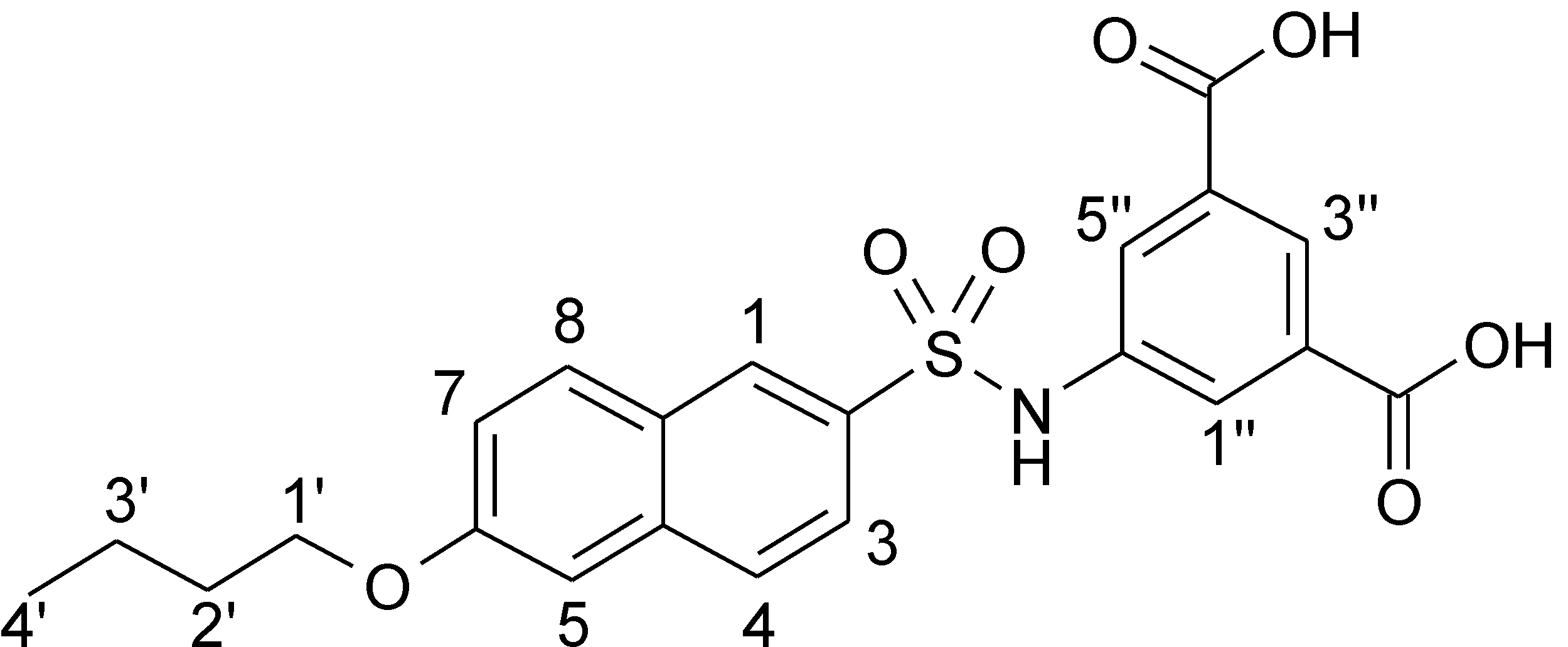
**

|  |  |
| --- | --- |
|  |  |
|  |  |
|  |  |

Selected ligand distances and dihedral angles during the MD simulation of the **5a**–MurD complex.

|  |  |
| --- | --- |
|  |  |
|  |  |

Selected ligand–protein distances indicating the interactions during the MD simulation of the **5a**–MurD complex.

|  |  |
| --- | --- |
|  |  |
|  |  |

Selected ligand–protein distances indicating the hydrogen bonds during the MD simulation of the **5a**–MurD complex.

Compound **5b**

**
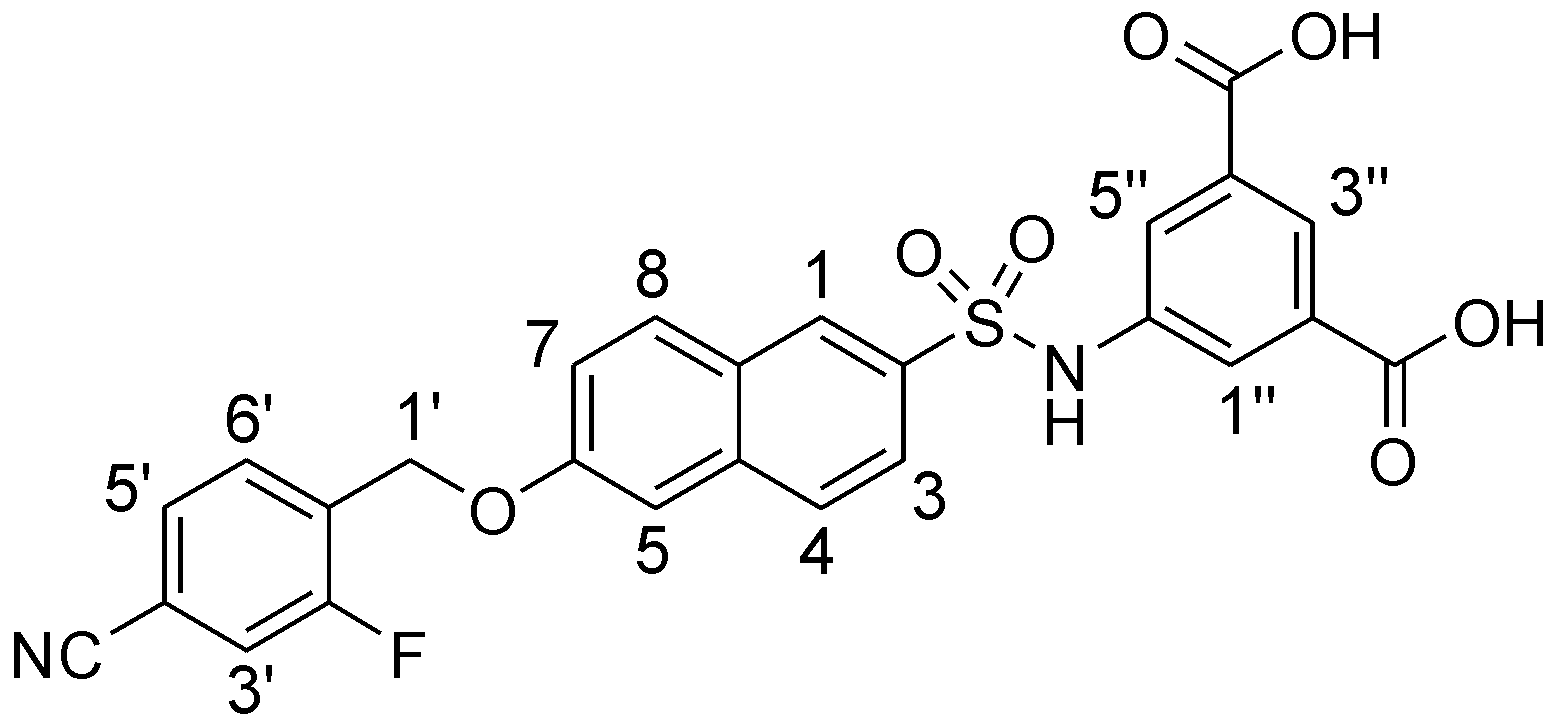
**

|  |  |
| --- | --- |
|  |  |
|  |  |
|  |  |

Selected ligand distances and dihedral angles during the MD simulation of the **5b**–MurD complex.

|  |  |
| --- | --- |
|  |  |
|  |  |
|  |  |

Selected ligand–protein distances indicating the interactions during the MD simulation of the **5b**–MurD complex.

|  |  |
| --- | --- |
|  |  |
|  |  |
|  |  |

Selected ligand–protein distances indicating the hydrogen bonds during the MD simulation of the **5b**–MurD complex.

Compound **6a**


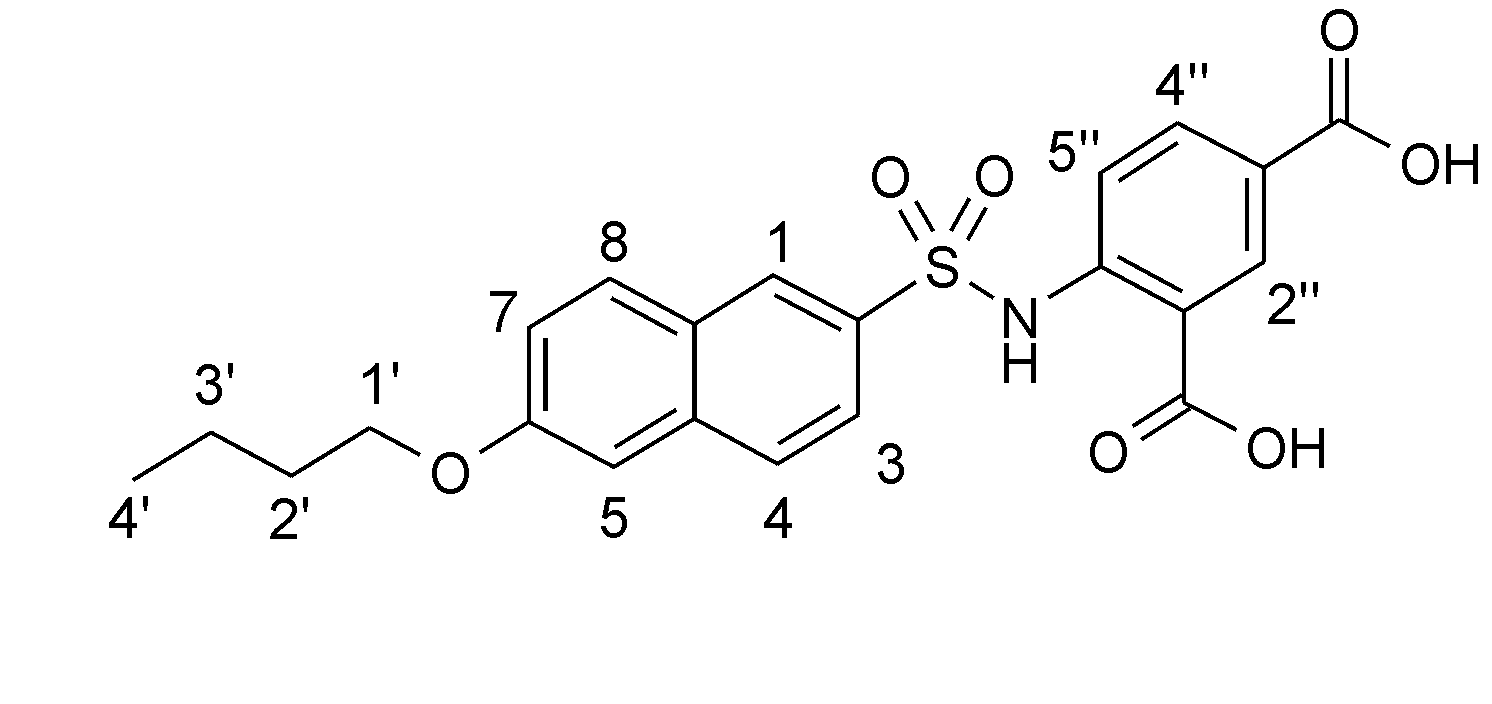


|  |  |
| --- | --- |
|  |  |
|  |  |
|  |  |

Selected ligand distances and dihedral angles during the MD simulation of the **6a**–MurD complex.

|  |  |
| --- | --- |
|  |  |
|  |  |

Selected ligand–protein distances indicating the interactions during the MD simulation of **6a**–MurD complex.

|  |  |
| --- | --- |
|  |  |
|  |  |
|  |  |

Selected ligand–protein distances indicating the hydrogen bonds during the MD simulation of the **6a**–MurD complex.

Compound **6b**

**
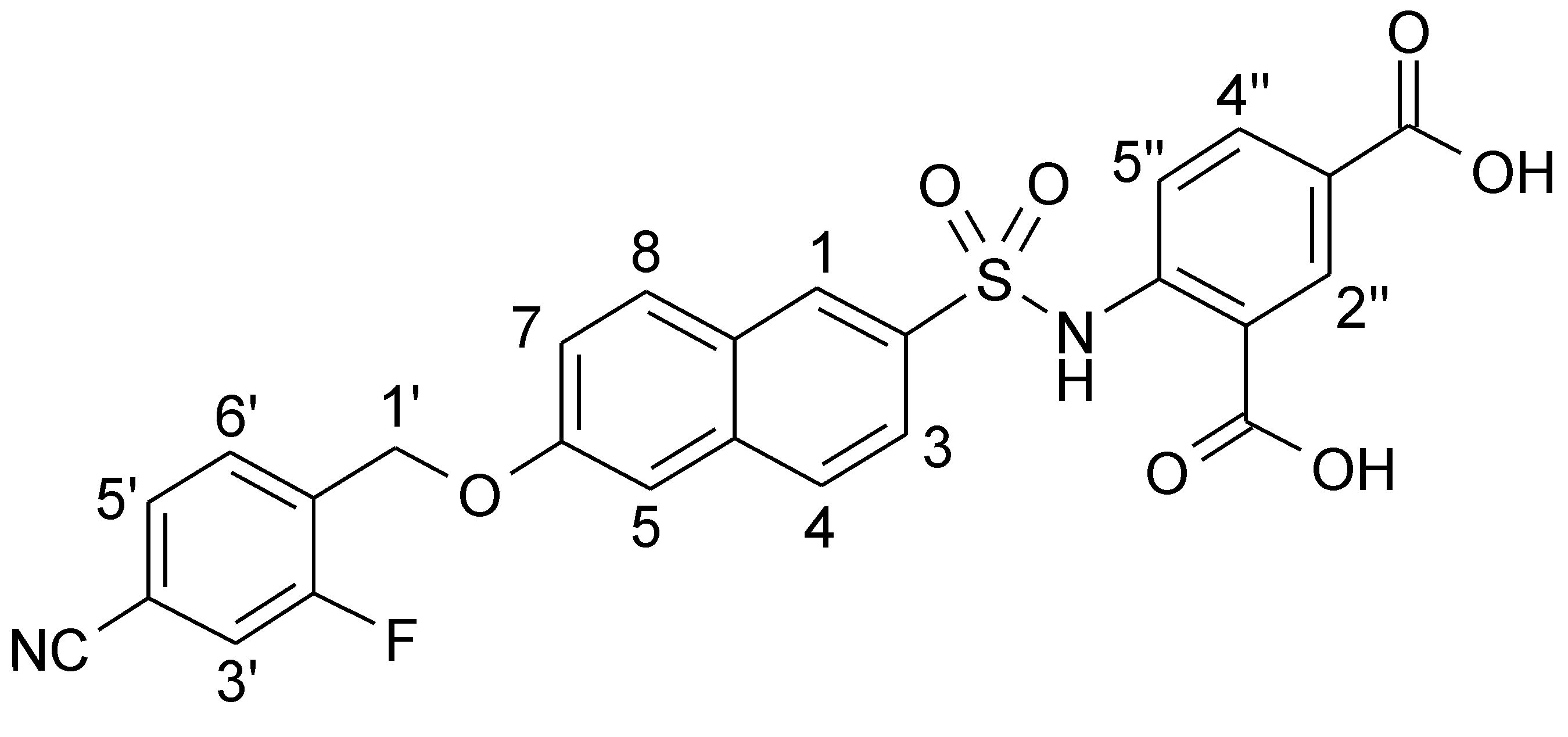
**

|  |  |
| --- | --- |
|  |  |
|  |  |
|  |  |

Selected ligand distances and dihedral angles during the MD simulation of the **6b**–MurD complex.

|  |  |
| --- | --- |
|  |  |
|  |  |
|  |  |

Selected ligand–protein distances indicating the interactions during the MD simulation of the **6b**–MurD complex.

|  |  |
| --- | --- |
|  |  |
|  |  |
|  |  |

Selected ligand–protein distances indicating the hydrogen bonds during the MD simulation of the **6b**–MurD complex.
