# Supplementary material for: The Binding Mode of Second-Generation Sulfonamide Inhibitors of MurD: Clues for Rational Design of Potent MurD Inhibitors
Source: PLoS One. 2012 Dec 20;7(12):e52817. doi: 10.1371/journal.pone.0052817 (PMC3527612; doi:10.1371/journal.pone.0052817)
Supplement: Dataset S4 — DMSO- d 6 effect on the protein. (DOC) [file pone.0052817.s014.doc]

**Dataset S4. The effect of DMSO-*d_6_* on the protein.** Overlays of ^1^H/^13^C HSQC NMR spectra in absence and presence of various amounts of DMSO-*d_6_*.

**
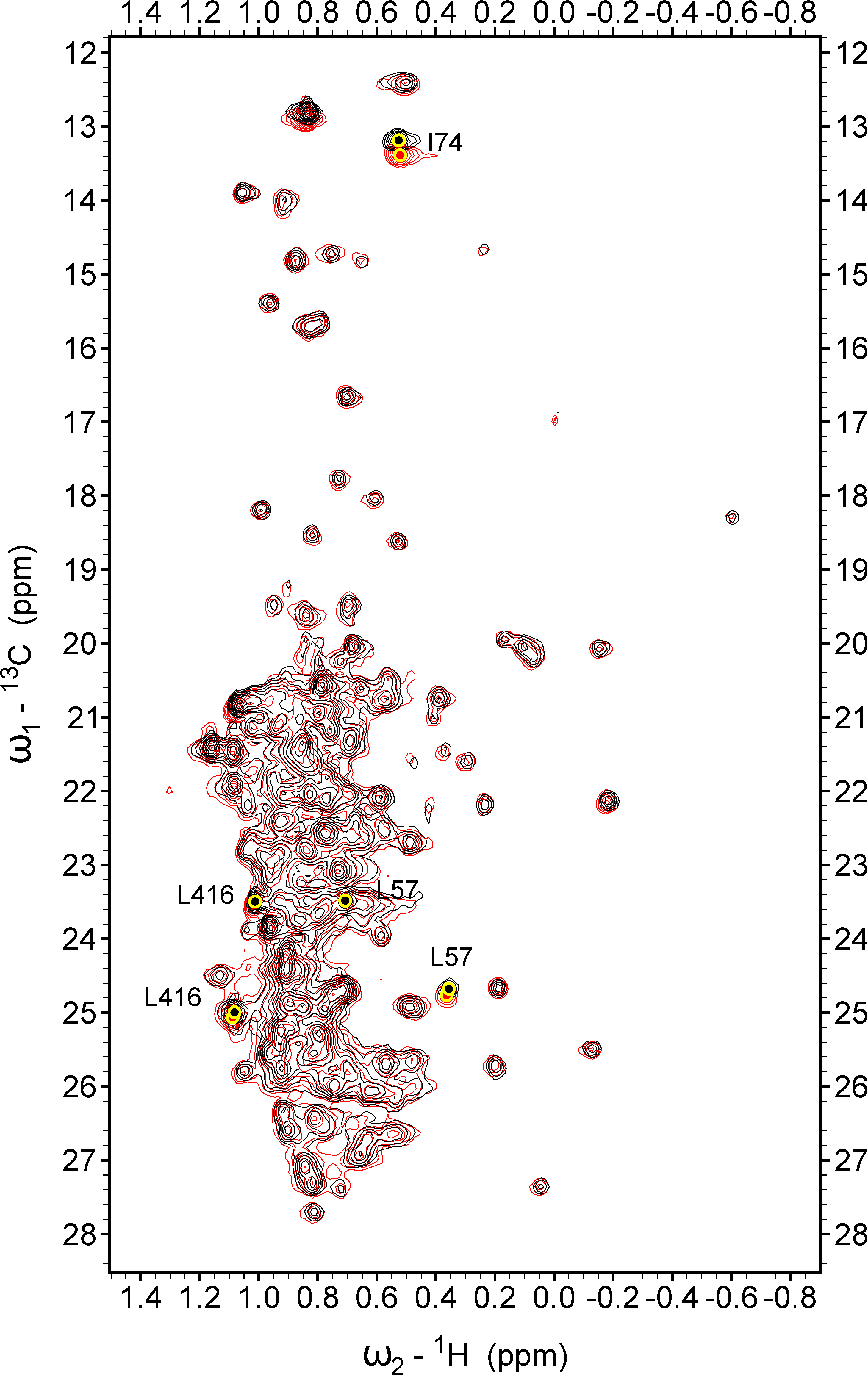
**

**Overlay of ^1^H/^13^C HSQC NMR spectra in absence (black) and presence (red) of 5% (v/v) DMSO-*d_6_* .**


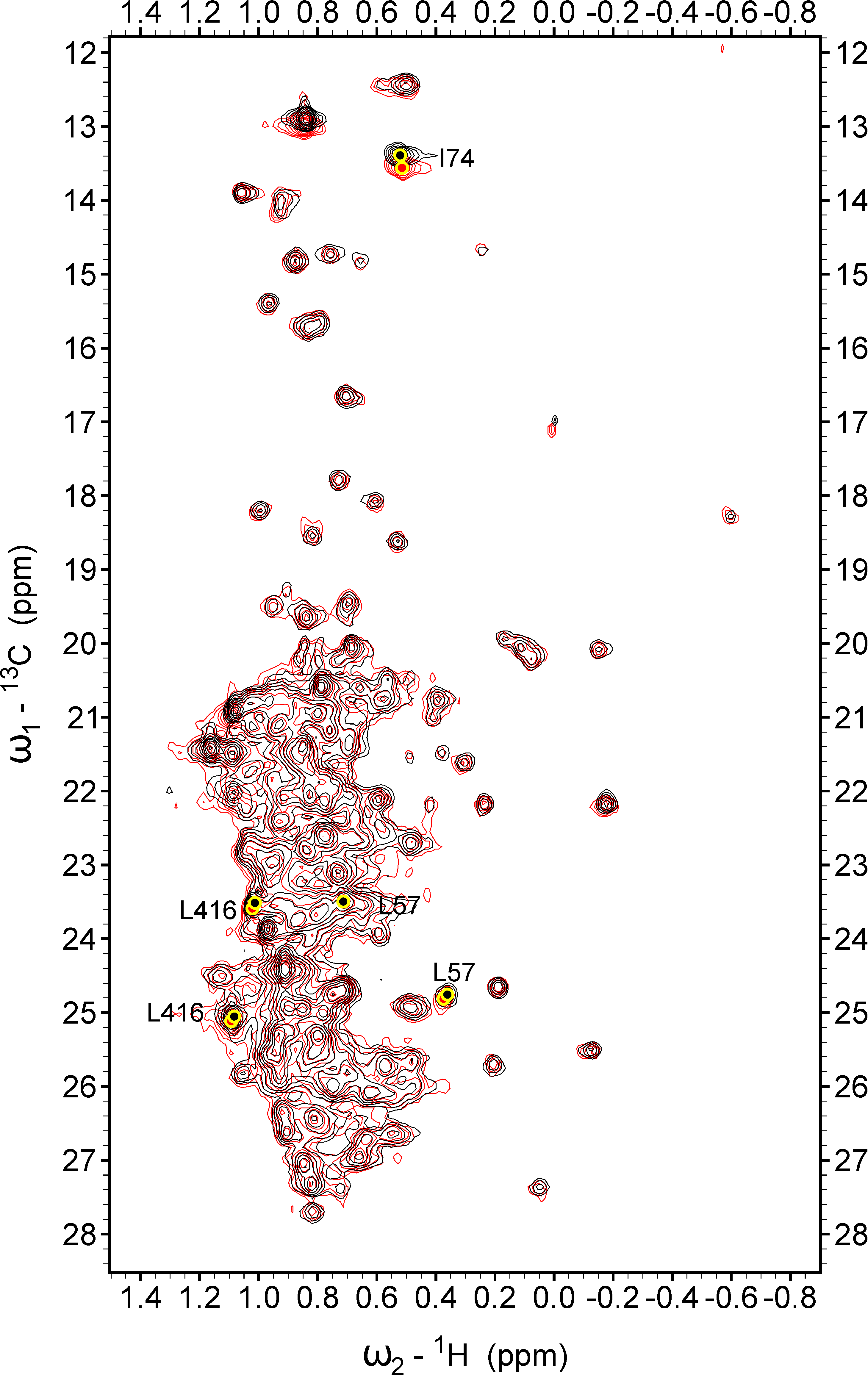


**Overlay of ^1^H/^13^C HSQC NMR spectra in presence of 5% (v/v) DMSO-*d_6_* (black) and 10% (v/v) DMSO-*d_6_* (red).**

**A)**


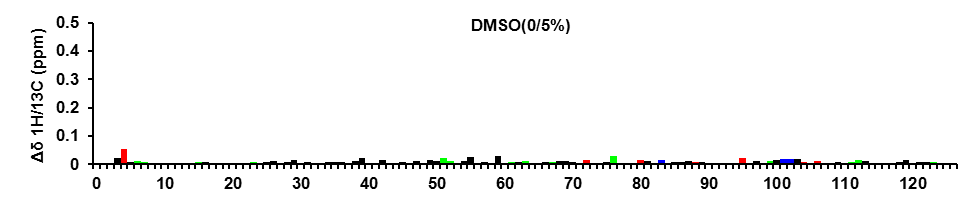


**B)**


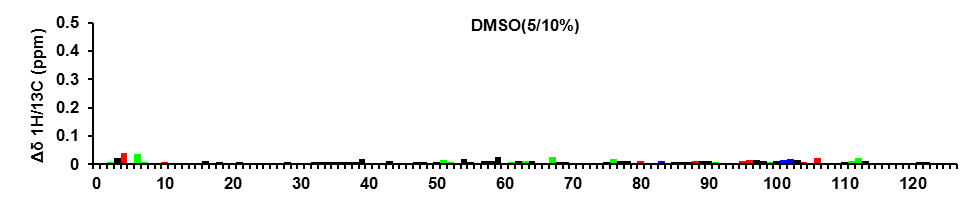


**C)**


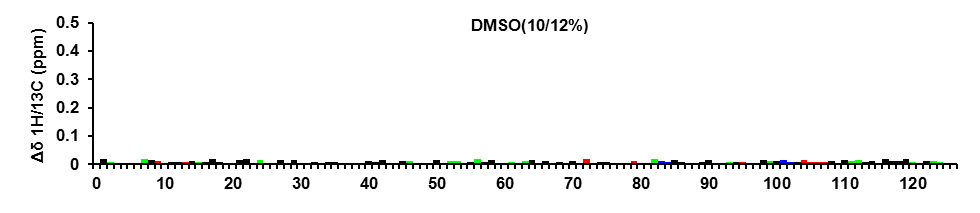


The CSP patterns of the ^13^C labeled methyl groups upon addition of DMSO-*d_6_*.

A) CSPs after addition of 5% (v/v) DMSO-*d_6_*; B) CSPs between 5% (v/v) and 10% (v/v) of DMSO-*d_6_*, C) CSPs between 10% (v/v) and 12% (v/v) DMSO-*d_6_* are shown. The CSPs in red, blue, and green correspond to the methyl groups near to the uracil binding site, the d-Glu binding site, and the cleft-forming region of the central domain respectively. No threshold is used. Note that the numbering of resonances does not correspond to the MurD residue numbers. The resonances are numbered according to the positions of the signals in the ^13^C dimension of the ^1^H/^13^C HSQC spectrum, starting from the most up-field position.
